# Novel anti-inflammatory and analgesic agents: synthesis, molecular docking and *in vivo* studies

**DOI:** 10.1080/14756366.2018.1426573

**Published:** 2018-01-26

**Authors:** David Izuchukwu Ugwu, Uchechukwu Christopher Okoro, Pius Onyeoziri Ukoha, Astha Gupta, Sunday N. Okafor

**Affiliations:** a Department of Pure and Industrial Chemistry, University of Nigeria, Nsukka, Nigeria;; b Department of Chemistry, Indian Institute of Technology, Kanpur, India;; c Department of Pharmaceutical and Medicinal Chemistry, University of Nigeria, Nsukka, Nigeria

**Keywords:** Analgesic, anti-inflammatory, benzothiazole, carboxamide, oral bioavailability, sulphonamide, ulcerogenic index

## Abstract

Twelve new derivatives of benzothiazole bearing benzenesulphonamide and carboxamide were synthesised and investigated for their *in vivo* anti-inflammatory, analgesic and ulcerogenic activities. Molecular docking showed an excellent binding interaction of the synthesised compounds with the receptors, with **17c** showing the highest binding energy (–12.50 kcal/mol). Compounds **17c** and **17i** inhibited carrageenan-induced rat paw oedema at 72, 76, and 80% and 64, 73, and 78% at 1 h, 2 h, and 3 h, respectively. In the analgesic activity experiment, compounds **17c**, **17 g**, and **17i** had ED_50_ (µM/kg) of 96, 127, and 84 after 0.5 h; 102, 134, and 72 after 1 h and 89, 156, and 69 µM/kg after 2 h, respectively, which were comparable with 156, 72, and 70 µM/kg for celecoxib. The ulcerogenic index of the most active derivatives **17c** and **17i** were 0.82 and 0.89, respectively, comparable to 0.92 for celecoxib. The physicochemical studies of the new derivatives showed that they will not have oral bioavailability problems.

## Introduction

Heterocycles bearing nitrogen, sulphur, and thiazole moieties constitute the core structure of a number of biologically active compounds. Benzothiazole (**1**) and their derivatives represent an important class of compounds possessing a wide spectrum of biological activities ranging from antitumour, antidiabetic, anti-inflammatory, anticonvulsant, antimalarial, etc^1^. Although the parent compound, benzothiazole is not widely used, many of its derivatives are found in commercial products. Most of the known derivatives of benzothiazole results from substitution at the methyne centre in the thiazole ring (**2**). Riluzole (**3**) used in treatment of amyotrophic lateral sclerosis^2^ and pramipexole (**4**), a dopamine agonist used in the treatment of Parkinson’s disease^3^, sexual dysfunction^4^, bipolar disorder^5^, clinical depression^6^ are good examples of benzothiazoles that aroused interest in further research into the pharmacological applications of benzothiazole derivatives.

Inflammation is part of the body’s immune response to stimuli. At first, it is beneficial because it initiates healing processes. However, it is of concern because inflammation can be self-perpetuating creating more inflammation in response to existing inflammation^7^. Inflammatory diseases are widely prevalent world over and inflammation remains a common and poorly controlled disease which can be life threatening in extreme form of allergy, autoimmune diseases, and rejection of organs transplanted^1^. Chronic inflammation has been linked to a variety of diseases including cardiovascular diseases, cancer, diabetes, arthritis, Alzheimer’s disease, pulmonary disease, etc.^1^.

Non-steroidal anti-inflammatory drugs (NSAIDs) are a drug class that include drugs that provide analgesic, antipyretic, and anti-inflammatory effects. The most prominent members of this group are aspirin (**5**), ibuprofen (**6**), naproxen (**7**), diclofenac (**8**), and indomethacin (**9**). Most NSAIDs inhibit the activity of cyclooxygenase-1 and cyclooxygenase-2 and thereby the synthesis of prostaglandins and thromboxanes. Inhibiting COX-2 leads to the desirable anti-inflammatory, analgesic and antipyretic activities whereas the inhibition of COX-1 leads to undesirable side effect like gastrointestinal bleeding^8^, kidney problems^9^, and central nervous system (CNS) effects^10^. The work of Caughey et al.^11^ and Varas-Lorenzo et al.^12^ has associated NSAIDs use with increased risk of stroke.

**Scheme 1.**
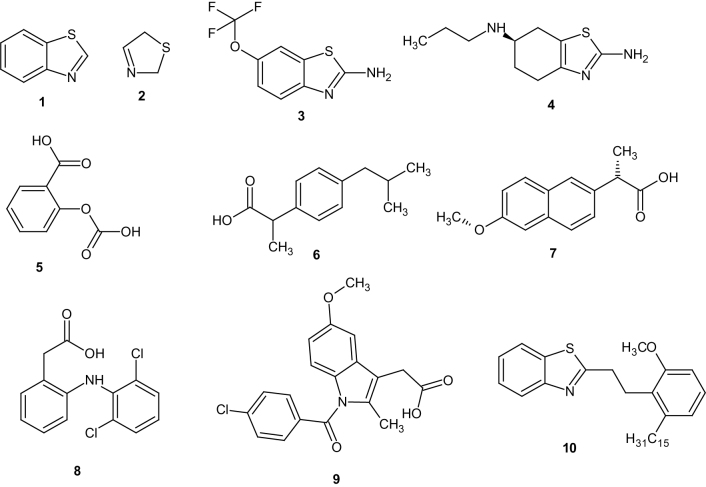
Examples of benzothiazoles and NSAIDS.

There is urgent need for the development of new anti-inflammatory agent given the associated risks with current anti-inflammatory drugs (Scheme 1).

There are wide reports on anti-inflammatory activities of benzothiazole derivatives. Paramashivappa et al.^13^ evaluated a series of benzothiazoles from anarcadic acid and found that compound **10** was 470-fold selective towards COX-2 compared to COX-1. Kumar et al.^14^ reported some oxadiazoles clubbed with benzothiazole nucleus that possessed comparable activity at micromolar concentration. Doma et al.^15^ reported the synthesis of some benzothiazole derivatives that had comparable percentage inhibition of carrageen induced rat paw oedema with diclofenac sodium. The work of Sadhasivano and Kulanthai^16^ revealed that amidation of 2-amino group of benzothiazole and incorporation of a phenylsulphonamido group gave a compound that inhibited inflammation at 94.45% in nanomolar concentration comparable with diclofenac (95.54%). Venkatesh and Pandeya^17^ reported some 2-aminobenzothiazoles that showed anti-inflammatory activities comparable to diclofenac. Gurupadyya et al.^18^, Srivastava et al.^19^, Geronikaki et al.^20^, Shafi et al.^21^ and many other researchers have reported various benzothiazole derivatives that possessed anti-inflammatory activities comparable with conventional drugs.

There have been wide reports on biological activities of benzothiazoles bearing sulphonamide moieties. Ibrahim et al.^22^ reported series of benzothiazole-6-sulphonamides that possessed *in silico* activities against human carbonic anhydrase isoforms in the nanomolar concentration. Kalina et al.^23^ reported 6-amino-2-benzothiazole sulphonamides as a topical carbonic anhydrase inhibitor. Jagtap et al.^24^ reported the anti-mycobacterial activity of some sulphonamide benzothiazoles. Argyropoulou et al.^25^ reported some sulphonamide thiazole and benzothiazoles that possessed antimicrobial activities in nanomolar concentration.

From the literature, it is abundantly clear that inflammatory diseases still cause untold hardship because of the risk associated with its current chemotherapeutic agents. Since benzothiazole derivatives have been reported to possess comparable anti-inflammatory activity, it is worthwhile that the search for new anti-inflammatory drugs should possess benzothiazole nucleus. Again, since sulphonamides have been reported to possess anti-inflammatory activities^26^, it will be expected that there will be synergistic anti-inflammatory effect in compounds that possessed both sulphonamide and benzothiazole moieties. Furthermore, the report of some biological activities on benzothiazoles possessing sulphonamides strengthened the desire to incorporate these functionalities in the design for novel anti-inflammatory agents.

We report herein the synthesis of some new carboxamides bearing benzothiazole and benzenesulphonamide. The new carboxamides possessed good anti-inflammatory, analgesic, and ulcerogenic activities.

## Experimental

### Synthesis of substituted benzenesulphonamoyl alkanamides (13a–l)

Sodium carbonate (Na_2_CO_3_, 1.590 g, 15 mmol) was added to a solution of amino acids (**12a–h**, 12.5 mmol) in water (15 ml) with continuous stirring until all the solutes dissolved. The solution was cooled to –5 °C and the appropriate benzenesulphonyl chloride (**11a**–**c**, 15 mmol) was added in four portions over a period of 1 h. The slurry was further stirred at room temperature for 4 h. The progress of the reaction was monitored using TLC (MeOH/DCM, 1:9). Upon completion, the mixture was acidified using 20% aqueous hydrochloric acid to pH 2. The crystals was filtered via suction and washed with pH 2.2 buffer. The pure products (**13a**–**l**) were dried over self-indicating fused silica gel in a desiccator^27^.

### Synthesis of N-benzoyl derivatives of benzenesulphonamides (15a–f)

Appropriate benzenesulphonamide (**13a**–**f**, 1.0 mmol) was dissolved in NaOH (10%, 10 ml) in a 50 ml round bottom flask, benzoyl chloride (**14**, 1.1 mmol, 0.2 ml) was added into the solution and stirred at room temperature. The reaction progress was monitored by TLC (3% MeOH/CH_2_Cl_2_) to the disappearance of the benzenesulphonamide spot. Upon completion, the solution was transferred into a beaker containing crushed ice and then acidified to pH 3 with concentrated hydrochloric acid. The solid was collected via suction filtration and transferred into a beaker containing CCl_4_ (10 ml) and covered with watch glass and boiled for 10 min. The mixture was allowed to cool slightly and then filtered. The products **(15a**–**f)** obtained were washed with 10–20 ml of CCl_4_ and dried over fused self-indicating silica gel in a desiccator^27^.

### Boric acid catalysed amidation of un-activated carboxylic acid and 2-aminobenzothiazole

To a suspension of substituted benzenesulphonamides (**15a–f**, **13g**–**l**, 1.0 mmol) in dry toluene (40 ml) equipped with Dean-Stark apparatus for azeotropic removal of water, was added 2-aminobenzothiazole (**16**, 1.0 mmol) and boric acid (0.1 mmol) at room temperature and then refluxed for 6 h. On completion (as monitored by TLC), the amide products were precipitated out from the reaction mixture by adding 40 ml *n*-hexane. The carboxamides (**17a**–**l**) were obtained via suction filtration, washed with *n*-hexane and dried over fused silica gel or concentrated using rotary evaporator and dried over vacuum in the case of oily products.

### N-(1,3-Benzothiazol-2-yl)-2-[N-(4-nitrobenzenesulfonyl)-1-phenylformamido] acetamide (17a)

Yield (0.3888 g, 78.37%), mp, 146.20–146.80 °C, FTIR (KBr, cm^−1^): 3347 (NH), 3106 (C–H aromatic), 1727, 1698 (C=O), 1640 (C=N), 1606, 1585, 1469, 1444 (C=C), 1525 (NO_2_), 1353, 1304 (2S=O), 1199, 1164 (SO_2_N), 1092, 1072, 1013 (C–N). ^1^H NMR (DMSO-d_6_, 400 MHz) *δ*: 8.36–8.30 (m, 3H, ArH), 8.02–7.98 (m, 3H, ArH), 7.91–7.89 (m, 1H, ArH), 7.64 (d, *J* = 7.32 Hz, 1H, ArH), 7.45 (t, *J* = 7.56 Hz, 1H, ArH), 7.31 (d, *J* = 7.80 Hz, 1H, ArH), 7.21 (t, *J* = 7.1 Hz, 2H, ArH), 7.02 (t, *J* = 7.10 Hz, 2H, ArH), 3.66 (s, 2H, CH_2_). ^13^C NMR (DMSO-d_6_, 400 MHz) *δ*: 170.66, 167.52 (C=O), 149.86, 146.99, 146.17, 133.39, 129.94, 129.78, 129.09, 128.91, 128.64, 126.38, 124.89, 124.73, 122.06, 121.84, 117.49 (15 aromatic carbons), 48.82 (aliphatic carbon). HRMS (*m*/*z*): 497.0612 (M + H), calculated: 497.0618.

### N-(1,3-Benzothiazol-2-yl)-2-[N-(4-nitrobenzenesulfonyl)-1-phenylformamido]-3-phenyl propanamide (17b)

Yield (0.4922 g, 83.98%), mp, 111.50–111.70 °C, FTIR (KBr, cm^−1^): 3414 (NH), 3087 (C–H aromatic), 2926 (C–H aliphatic), 1700, 1687 (C=O), 1639 (C=N), 1607, 1583, 1470, 1455 (C=C), 1530, 1496 (NO_2_), 1349, 1310 (2S=O), 1160 (SO_2_N), 1092, 1013 (C–N). ^1^H NMR (DMSO-d_6_, 400 MHz) *δ*: 8.14 (d, *J* = 9.16 Hz, 2H, ArH), 7.91 (d, *J* = 7.32 Hz, 1H, ArH), 7.70 (d, *J* = 8.72 Hz, 2H, ArH), 7.45 (d, *J* = 9.16 Hz, 2H, ArH), 7.29 (d, *J* = 7.76 Hz, 1H, ArH), 7.21–7.15 (m, 3H, ArH), 7.12–7.07 (m, 5H, ArH), 6.95 (t, *J* = 7.56 Hz, 1H, ArH), 3.96 (dd, *J* = 3.64, 6.40 Hz, 1H, CH–C=O), 2.96 (dd, *J* = 4.56, 5.04 Hz, 1H, CH_a_ of CH_2_), 2.68 (dd, *J* = 10.56, 10.52 Hz, 1H, CH_b_ of CH_2_). ^13^C NMR (DMSO-d_6_, 400 MHz) *δ*: 172.72, 167.00 (C=O), 153.30 (C=N), 149.58, 147.16, 137.86, 137.22, 131.43, 129.79, 129.72, 129.41, 129.07, 128.71, 128.61, 128.44, 128.16, 126.87, 125.92, 125.82, 124.62, 121.33, 118.23 (19 aromatic carbons), 58.23, 38.09 (two aliphatic carbons). HRMS (*m*/*z*): 586.0991 (M^+^), calculated, 586.0981.

### N-(1,3-Benzothiazol-2-yl)-3-(1H-indol-2-yl)-2-[N-(4-nitrobenzenesulfonyl)-1-phenyl formamido]propanamide (17c)

Yield (0.6209 g, 99.33%), mp, 140.00–140.40 °C, FTIR (KBr, cm^−1^): 3413, 3362 (2NH), 3004 (C–H aromatic), 2984 (C–H aliphatic), 1693, 1640 (C=O), 1619 (C=N), 1601, 1458, 1403 (C=C), 1528 (NO_2_), 1349, 1312 (2S=O), 1163 (SO_2_N), 1091, 1012 (C–N). ^1^H NMR (DMSO-d_6_, 400 MHz) *δ*: 10.80 (NH of indole), 8.66 (s, 1H, NH of amide), 7.93 (d, *J* = 7.36 Hz, 1H, ArH), 7.83 (d, *J* = 8.68 Hz, 2H, ArH), 7.58 (d, *J* = 7.80 Hz, 1H, ArH), 7.46 (t, *J* = 8.24 Hz, 2H, ArH), 7.19 (t, *J* = 7.54 Hz, 4H, ArH), 7.12–7.04 (m, 5H, ArH), 6.97–6.80 (m, 3H, ArH), 3.94 (dd, *J* = 4.60, 2.76 Hz, 1H, CH–C=O), 3.08 (dd, *J* = 4.12, 3.68 Hz, 1H, CH_a_ of CH_2_), 2.83 (dd, *J* = 10.52, 10.08 Hz, 1H, CH_b_ of CH_2_). ^13^C NMR (DMSO-d_6_, 400 MHz) *δ*: 173.44, 167.09 (C=O), 153.38 (C=N), 148.83, 146.49, 137.85, 137.55, 136.50, 129.40, 129.03, 128.70, 128.17, 127.34, 126.99, 125.81, 125.48, 124.96, 123.71, 121.32, 121.27, 121.12, 118.71, 118.24, 111.72, 109.21 (23 aromatic carbons), 57.16, 28.19 (two aliphatic carbons). HRMS (*m*/*z*): 625.1110 (M^+^), calculated, 625.1109.

### N-(1,3-Benzothiazol-2-yl)-4-methyl-2-[N-(4-nitrobenzenesulfonyl)-1-phenylformamido] pentanamide (17d)

Yield (0.4824 g, 87.78%), mp, 197.20–198.10 °C, FTIR (KBr, cm^−1^): 3415 (NH), 3101 (C–H aromatic), 2958 (C–H aliphatic), 1692, 1654 (C=O), 1620 (C=N), 1607, 1568, 1437 (C=C), 1531, 1497 (NO_2_), 1349, 1312 (2S=O), 1182, 1166 (SO_2_N), 1092, 1003 (C–N). ^1^H NMR (DMSO-d_6_, 400 MHz) *δ*: 8.34 (d, *J* = 8.72 Hz, 2H, ArH), 7.98 (d, *J* = 8.68 Hz, 2H, ArH), 7.90 (d, *J* = 7.80 Hz, 1H, ArH), 7.58 (t, *J* = 7.76 Hz, 1H, ArH), 7.44 (t, *J* = 7.76 Hz, 1H, ArH), 7.27 (d, *J* = 7.76 Hz, 1H, ArH), 7.19 (t, *J* = 7.32 Hz, 2H, ArH), 7.15–7.07 (m, 2H, ArH), 6.94 (t, *J* = 7.56 Hz, 1H, ArH), 6.52 (s, 1H, NH), 3.72 (t, *J* = 2.76 Hz, 1H, CH–C=O), 1.41–1.34 (m, 1H, CH), 1.22–1.18 (m, 2H, CH_2_), 0.82–0.76 (m, 6H, 2CH_3_). ^13^C NMR (DMSO-d_6_, 400 MHz) *δ*: 173.37, 167.85 (C=O), 153.31 (C=N), 149.90, 147.29, 137.86, 133.37, 131.30, 129.79, 129.41, 129.08, 128.72, 128.65, 125.91, 124.82, 121.35, 118.24 (15 aromatic carbons), 54.71, 31.48, 23.13, 14.48 (four aliphatic carbons). HRMS (*m*/*z*): 552.1138 (M^+^), calculated, 552.1137.

### N-(1,3-Benzothiazol-2-yl)-3-methyl-2-[N-(4-nitrobenzenesulfonyl)-1-phenylformamido] pentanamide (17e)

Yield (0.5514 g, 99.87%), mp, 158.30–158.90 °C, FTIR (KBr, cm^−1^): 3391 (NH), 3065 (C–H aromatic), 2957 (C–H aliphatic), 1693, 1640 (C=O), 1613 (C=N), 1602, 1583, 1469, 1455 (C=C), 1529 (NO_2_), 1349, 1308 (2S=O), 1164, 1143 (SO_2_N), 1092, 1067, 1020 (C–N). ^1^H NMR (DMSO-d_6_, 400 MHz) *δ*: 8.34 (d, *J* = 8.68 Hz, 2H, ArH), 8.98 (d, *J* = 8.24 Hz, 2H, ArH), 7.91 (d, *J* = 7.32 Hz, 1H, ArH), 7.59 (t, *J* = 7.80 Hz, 1H, ArH), 7.45 (t, *J* = 7.76 Hz, 1H, ArH), 7.28 (d, *J* = 8.28 Hz, 1H, ArH), 7.20 (d, *J* = 7.34 Hz, 2H, ArH), 7.13–7.08 (m, 2H, ArH), 6.95 (t, *J* = 7.32 Hz,1H, ArH), 6.44 (s, 1H, NH), 3.73 (d, *J* = 5.15 Hz, 1H, CH–C=O), 1.42–1.34 (m, 1H, CH), 1.22–1.18 (m, 2H, CH_2_), 0.80–0.70 (m, 6H, 2CH_3_). ^13^C NMR (DMSO-d_6_, 400 MHz) *δ*: 173.38, 167.86 (C=O), 166.98 (C=N), 153.33 (C–NO_2_), 149.89, 147.30, 137.86, 133.35, 131.45, 129.79, 129.41, 129.07, 128.72, 125.91, 125.83, 124.82, 121.36, 121.29, 118.22 (17 aromatic carbons), 54.74, 31.49, 24.49, 23.13, 14.47 (five aliphatic carbons). HRMS (*m*/*z*): 553.1242 (M + H), calculated, 553.1249.

### N-(1,3-Benzothiazol-2-yl)-3-methyl-2-[N-(4-nitrobenzenesulfonyl)-1-phenylformamido] butanamide (17f)

Yield (0.5309 g, 98.66%), mp, 162.90–163.30 °C, FTIR (KBr, cm^−1^): 3398 (NH), 3110 (C–H aromatic), 2987, 2899 (C–H aliphatic), 1696, 1659 (C=O), 1618 (C=N), 1601, 1592, 1487 (C=C), 1531, 1515 (NO_2_), 1356, 1334 (2S=O), 1168, 1139 (SO_2_N), 1094, 1071 (C–N). ^1^H NMR (DMSO-d_6_, 400 MHz) *δ*: 8.34 (d, *J* = 7.80 Hz, 2H, ArH), 8.00 (d, *J* = 8.72 Hz, 1H, ArH), 7.61 (d, *J* = 7.80 Hz, 1H, ArH), 7.29 (t, *J* = 8.70 Hz, 2H, ArH), 7.20 (t, *J* = 7.56 Hz, 2H, ArH), 7.13–7.08 (m, 4H, ArH), 7.98 (t, *J* = 7.34 Hz, 1H, ArH), 6.54 (s, 1H, NH), 3.60 (d, *J* = 5.96 Hz, 1H, CH-C=O), 1.99–1.94 (m, 1H, CH), 0.81–0.76 (m, 6H, 2CH_3_). ^13^C NMR (DMSO-d_6_, 400 MHz) *δ*: 172.42, 167.22 (C=O), 160.11 (C=N), 150.26 (C–NO_2_), 137.87, 133.87, 131.09, 130.83, 129.42, 129.09, 128.72, 128.45, 126.11, 125.83, 124.76, 121.56, 117.92 (15 aromatic carbons), 61.97, 31.51, 19.56, 18.24 (four aliphatic carbons). HRMS (*m*/*z*): 538.0980 (M^+^), calculated, 538.0981.

### N-(1,3-Benzothiazol-2-yl)-4-hydroxy-1-(4-nitrobenzenesulfonyl)pyrrolidine-2-carboxamide (17g)

Yield (0.4479 g, 99.96%), mp, 127.40–127.90 °C, FTIR (KBr, cm^−1^): 3492 (NH), 3389 (OH), 3118 (C–H aromatic), 2987, 2899 (C–H aliphatic), 1750 (C=O), 1646 (C=N), 1608, 1451, 1403 (C=C), 1528 (NO_2_), 1354, 1331 (2S=O), 1184, 1164 (SO_2_N), 1090, 1069, 1016, 1005 (C–N, C–O). ^1^H NMR (DMSO-d_6_, 400 MHz) *δ*: 8.35 (d, *J* = 8.72 Hz, 2H, ArH), 8.02 (d, *J* = 8.72 Hz, 2H, ArH), 7.59 (d, *J* = 7.80 Hz, 1H, ArH), 7.27 (d, *J* = 7.76 Hz, 1H, ArH), 7.20–7.14 (m, 2H, ArH), 6.50 (s, 1H, NH), 4.16 (s, 1H, OH), 4.09 (t, *J* = 5.32 Hz, 1H, CH–C=O), 3.46–3.42 (m, 1H, CH-OH), 3.19 (d, *J* = 5.95 Hz, 2H, CH_2_N), 2.03–1.98 (m, 1H, CH of CH_2_), 1.92–1.86 (m, 1H, CH of CH_2_). ^13^C NMR (DMSO-d_6_, 400 MHz) *δ*: 175.76 (C=O), 173.53, 166.95, 163.69, 150.32, 143.47, 129.53, 125.95, 124.85, 121.39, 121.34, 118.20 (11 aromatic carbons), 68.99, 60.38, 57.14, 30.77 (four aliphatic carbons). HRMS (*m*/*z*): 449.0586 (M + H), calculated, 449.0588.

### N-(1,3-Benzothiazol-2-yl)-1-(4-nitrobenzenesulfonyl)pyrrolidine-2-carboxamide (17h)

Yield (0.4320 g, 99.98%), mp, 155.10 °C, FTIR (KBr, cm^−1^): 3419 (NH), 3106 (C–H aromatic), 2981 (C–H aliphatic), 1703 (C=O), 1623 (C=N), 1604, 1454, 1401 (C=C), 1531, 1495 (NO_2_), 1350, 1313 (2S=O), 1200, 1164 (SO_2_N), 1093, 1065, 1011 (C–N). ^1^H NMR (DMSO-d_6_, 400 MHz) *δ*: 8.4193–8.3574 (m, 2H, ArH), 8.1146–8.0447 (m, 2H, ArH), 7.9725–7.9531 (d, *J* = 7.76 Hz, 1H, ArH), 7.7469–7.7274 (d, *J* = 7.80 Hz, 1H, ArH), 7.2211–7.0779 (m, 2H, ArH), 6.5029 (s, 1H, NH), 4.4399–4.4089 (dd, *J* = 4.16, 4.12 Hz, 1H, CH–C=O), 4.1890–4.1581 (m, 1H, CH_a_ of CH_2_N), 3.5555–3.5006 (m, 1H, CH_b_ of CH_2_N), 3.2279–3.1672 (m, 1H, CH_a_ of CH_2_), 2.0103–1.7457 (m, 2H, CH_2_), 1.6368–1.5899 (m, 1H, CH_b_ of CH_2_). ^13^C NMR (DMSO-d_6_, 400 MHz) *δ*: 173.4140 (C=O), 166.9951 (C=N), 150.4116 (C–NO_2_), 143.7725, 129.3732, 128.7314, 125.8381, 125.1387, 122.3317, 121.3833, 121.1725, 118.1739 (11 aromatic carbons), 61.0656, 48.9465, 31.5774, 24.9670 (four aliphatic carbons). HRMS (*m*/*z*): 433.0649 (M + H), calculated, 433.0652.

### N-(1,3-Benzothiazol-2-yl)-4-hydroxy-1-(4-methylbenzenesulfonyl)pyrrolidine-2-carboxamide (17i)

Yield (0.4171 g, 100%), mp, 177.00 °C, FTIR (KBr, cm^−1^): 3412 (NH), 3376 (OH), 3004 (C–H aromatic), 2949 (C–H aliphatic), 1725 (C=O), 1647 (C=N), 1598, 1495, 1470, 1404 (C=C), 1333, 1312 (2S=O), 1198, 1155 (SO_2_N), 1088, 1011 (C–N). ^1^H NMR (DMSO-d_6_, 400 MHz) *δ*: 7.6530–7.6323 (d, *J* = 8.28 Hz, 2H, ArH), 7.6117–7.5945 (d, *J* = 6.88 Hz, 1H, ArH), 7.4788 (s, 1H, NH), 7.3666–7.3460 (d, *J* = 8.24 Hz, 2H, ArH), 7.3001–7.2807 (d, *J* = 7.76 Hz, 1H, ArH), 7.2016–7.1146 (m, 1H, ArH), 6.9863–6.9485 (t, *J* = 7.56 Hz, 1H, ArH), 4.1718 (s, 1H, OH), 4.0172–3.9782 (t, *J* = 7.80 Hz, 1H, CH–C=O), 3.4318–3.3940 (m, 1H, CH-OH), 3.0618–3.0309 (d, *J* = 6.82 Hz, 2H, CH_2_N), 2.3413 (s, 3H, CH_3_-Ar), 1.9175–1.8866 (m, 2H, CH_2_). ^13^C NMR (DMSO-d_6_, 400 MHz) *δ*: 173.8163 (C=O), 167.0622 (C=N), 152.8354, 143.7629, 134.9298, 131.1935, 130.1013, 129.4307, 128.7314, 127.9745, 126.0297, 121.4599, 118.1355 (11 aromatic carbons), 68.9119, 60.1650, 56.7736, 21.5276 (four aliphatic carbons). HRMS (*m*/*z*): 416.0736 (M–H), calculated, 416.0739.

### N-(1,3-Benzothiazol-2-yl)-1-(4-methylbenzenesulfonyl)pyrrolidine-2-carboxamide (17j)

Yield (0.4010 g, 99.98%), pale yellowish oil, FTIR (KBr, cm^−1^): 3356 (NH), 3021 (C–H aromatic), 2994 (C–H aliphatic), 1701 (C=O), 1623 (C=N), 1608, 1495, 1470 (C=C), 1339, 1322 (2S=O), 1198, 1155 (SO_2_N), 1088, 1071 (C–N). ^1^H NMR (CDOD_3_, 400 MHz) *δ*: 7.6530–7.6323 (d, *J* = 8.28 Hz, 2H, ArH), 7.6117–7.5945 (d, *J* = 6.88 Hz, 1H, ArH), 7.3011–7.2817 (d, *J* = 7.76 Hz, 1H, ArH), 7.2211–7.0779 (m, 2H, ArH), 6.5007 (s, 1H, NH), 4.4399–4.4089 (dd, *J* = 4.16, 4.12 Hz, 1H, CH–C=O), 4.1890–4.1581 (m, 1H, CH_a_ of CH_2_N), 3.5555–3.5006 (m, 1H, CH_b_ of CH_2_N), 3.2279–3.1672 (m, 1H, CH_a_ of CH_2_), 2.0103–1.7457 (m, 2H, CH_2_), 1.6368–1.5899 (m, 1H, CH_b_ of CH_2_). ^13^C NMR (CDOD_3_, 400 MHz) *δ*: 174.7552 (C=O), 168.7388 (C=N), 152.1176 (C–NO_2_), 144.0216, 137.5740, 134.7574, 129.7565, 128.6164, 125.6369, 124.9855, 123.8646, 116.8326 (11 aromatic carbons), 60.7303, 30.7151, 24.2963, 22.2803, 20.1864 (five aliphatic carbons). HRMS (*m*/*z*): 400.0796 (M–H), calculated, 400.0801.

### 1-(Benzenesulfonyl)-N-(1,3-benzothiazol-2-yl)-4-hydroxypyrrolidine-2-carboxamide (17k)

Yield (0.4031 g, 100%), pale yellowish oil, FTIR (KBr, cm^−1^): 3432 (NH), 3316 (OH), 3098 (C–H aromatic), 2988, 2879 (C–H aliphatic), 1692 (C=O), 1619 (C=N), 1605, 1592, 1454 (C=C), 1361, 1319 (2S=O), 1179, 1136 (SO_2_N), 1095, 1073, 1027 (C–N, C–O). ^1^H NMR (CDOD_3_, 400 MHz) *δ*: 7.8587–7.8404 (d, *J* = 7.32 Hz, 2H, ArH), 7.6312–7.5945 (t, *J* = 7.34 Hz, 1H, ArH), 7.5579–7.5197 (t, *J* = 7.64 Hz, 3H, ArH), 7.3685–7.3487 (d, *J* = 7.92 Hz, 1H, ArH), 7.2540–7.2143 (t, *J* = 7.94 Hz, 1H, ArH), 7.1791–7.0325 (m, 1H, ArH), 6.5405 (s, 1H, NH), 4.3021 (s, 1H, OH), 4.2410–4.2013 (t, *J* = 7.94 Hz, 1H, CH–C=O), 3.5783–3.5416 (m, 1H, CH-OH), 3.2805–3.2515 (d, *J* = 11.60 Hz, 2H, CH_2_N), 2.0665–2.0008 (m, 2H, CH_2_). ^13^C NMR (CDOD_3_, 400 MHz) *δ*: 174.9756 (C=O), 150.5553 (C=N), 137.5548, 132.7743, 129.9864, 128.7888, 128.6068, 127.8979, 127.5338, 125.7232, 124.9855, 121.7569, 120.6935, 117.1775 (13 aromatic carbons), 69.1993, 60.0501, 56.2850, 39.0117 (four aliphatic carbons). HRMS (*m*/*z*): 422.0832 (M + H_3_O), calculated, 422.0837.

### 1-(Benzenesulfonyl)-N-(1,3-benzothiazol-2-yl)pyrrolidine-2-carboxamide (17l)

Yield (0.3864 g, 99.82%), pale yellowish oil, FTIR (KBr, cm^−1^): 3418 (NH), 3020 (C–H aromatic), 2987, 2895 (C–H aliphatic), 1693 (C=O), 1638 (C=N), 1601, 1447 (C=C), 1385, 1339 (2S=O), 1161 (SO_2_N), 1094, 1049, 1025 (C–N). ^1^H NMR (DMSO-d_6_, 400 MHz) *δ*: 7.7755–7.7572 (d, *J* = 7.32 Hz, 2H, ArH), 7.6747 (s, 1H, NH), 7.6381–7.6026 (t, *J* = 7.10 Hz, 1H, ArH), 7.5716–7.5522 (d, *J* = 7.76 Hz, 2H, ArH), 7.2990–7.2784 (d, *J* = 8.24 Hz, 1H, ArH), 7.1787–7.1455 (t, *J* = 6.64 Hz, 1H, ArH), 7.0962–7.0424 (m, 1H, ArH), 6.9886–6.9508 (t, *J* = 7.56 Hz, 1H, ArH), 4.0630–4.0321 (t, *J* = 6.18 Hz, 1H, CH–C=O), 3.3505–3.3104 (m, 1H, CH_a_ of CH_2_N), 3.1855–3.0973 (m, 1H, CH_b_ of CH_2_N), 1.8911–1.7411 (m, 3H), 1.5406–1.4982 (m, 1H, CH of CH_2_). ^1^H NMR (DMSO-d_6_, 400 MHz) *δ*: 173.6165 (C=O), 167.3809 (C=N), 151.2674, 138.0430, 133.4187, 129.7574, 129.2998, 128.5847, 127.5168, 126.0771, 125.6957, 121.7293, 121.4528, 117.6866 (13 aromatic carbons), 60.8415, 48.8660, 30.9410, 24.7245 (four aliphatic carbons). HRMS (*m*/*z*): 388.0784 (M + H), calculated, 388.0781.

## Molecular studies

The default parameters of MOE program were used for the molecular docking of the compounds. To find the correct conformations of the ligands and to obtain minimum energy structures, ligands were allowed to be flexible whereas the proteins were rigid. At the end of docking, the best conformations of the ligands were analysed for their binding interactions.

## Biological studies

### *In vivo* anti-inflammatory activities determination

Male albino rats weighing 300 g where purchased from the Department of Biochemistry, University of Nigeria, Nsukka and kept at room temperature in a light controlled animal house. They were fasted with free access to water at least 12 h prior to the experiments. The tested compounds were prepared as suspension in vehicle (0.5% methylcellulose) and celecoxib was used as a standard drug. The positive control received celecoxib while the negative control received only the vehicle. Oedema was produced by injecting 0.2 ml of a solution of 1% carrageenan in the hind paw. The rats were injected intraperitoneally with 1 ml suspension in 0.5% methylcellulose of the tested compounds and reference drug. Paw volume was measured by water displacement with a plethysmometer (UGO BASILE) before, 0.5 h, 1 h, 2 h, and 3 h after treatment. The percentage was calculated by the following equation^28^:

Anti-inflammatory activity (%)=(1−D/C)×100

where *D* represents the difference in paw volume before and after drug administration to the rats and *C* represents the difference of volume in the control groups. The approval for the use of animal was obtained from the University of Nigeria committee on experimental animal use.

### Ulcerogenic activity

Male albino rats weighing 200–250 g were fasted for 12 h prior to drug administration. Water was supplied ad libitum. The animals were divided into seven equal groups (each of four). The first group received 7% gum acacia (suspending vehicle) orally once a day and was left as a control, whereas the other groups received the reference drug and test compounds with a dose of 100 mmol/kg/day orally. The test compounds were administered once a day for three successive days. The animals were killed by an overdose of ether 6 h after the last dose. The stomachs were removed, opened along the greater curvature, and examined for ulceration. The number and diameter of discrete areas of damage in the glandular mucosa were scored (Table 2). The ulcer score was calculated according to the method of Vijaya Kumar and Mishra^29^: 0.0 – normal (no injury); 0.5 – latent injury; 1.0 – slight injury (two to three dotted lines); 2.0 – severe injury (continuous lined injury or five to six dotted injuries); 3.0 – very severe injury (several continuous lined injuries); 4.0 – widespread lined injury.

### Analgesic activity

Male albino swiss mice (25 g body weight) were divided into various groups (*N* = 4). Each mouse was initially placed on a hot plate thermostatically maintained at 58 °C (Colombus Co., Shanghai, China)^30^. The mouse was watched carefully for the time in seconds in which it displays nociceptive responses exhibited as licking or blowing (fanning) its front paws. This time was considered as the control reaction time. A cutoff time of 60 s was used to avoid damage to the paws. To test the analgesic activity of the compounds each group of mice was treated with one dose of the test compounds (5–200 mg/kg i.p.). The reaction time was then retested at 15, 30, 60, and 120 min after injection (each animal acted as its own control). The percentage changes in the reaction were then calculated. The ED_50_ for each compound was then calculated by linear regression.

### *In vitro* cyclooxygenase inhibitory assay

The *in vitro* ability of the most active compounds and celecoxib to inhibit the COX-1 and COX-2 isozymes was carried out using Cayman colorimetric COX (ovine) inhibitor screening assay kit supplied by Cayman Chemicals (Ann Arbor, MI). The calculations were performed as per the kit guidelines^31^.

## Results and discussion

### Chemistry

**Scheme 2.**
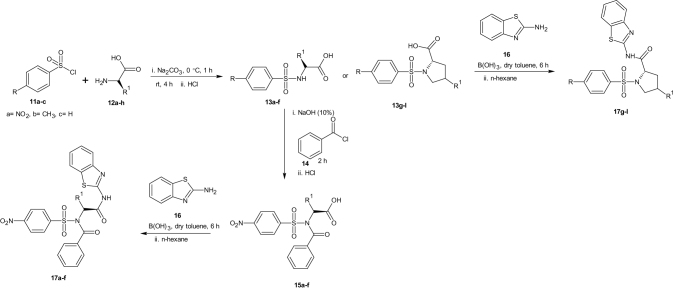
Synthetic route to the new benzothiazoles.

**Scheme 3.**
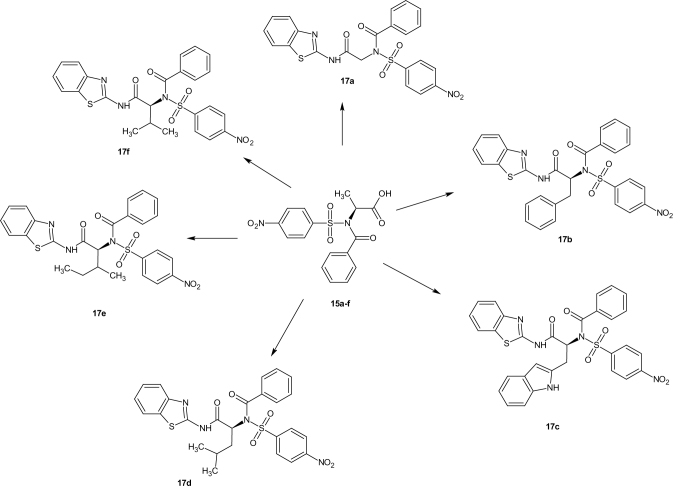
N-benzoylated benzenesulphonamide derivatives (**17a**–**f**).

**Scheme 4.**
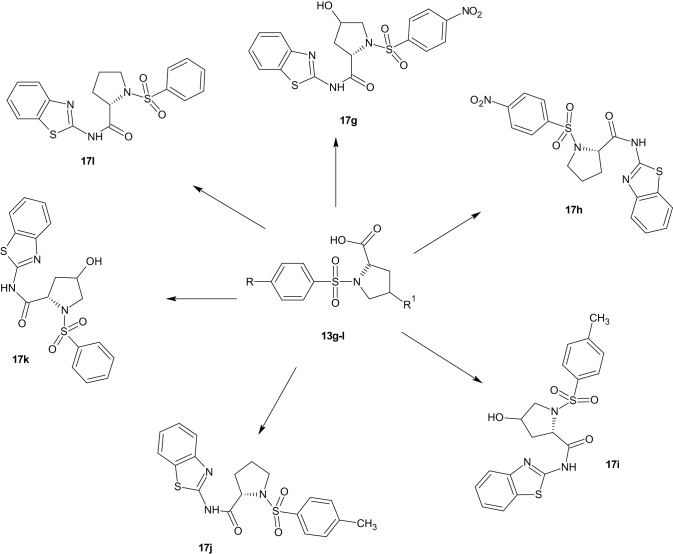
Proline derived benzenesulphonamides (**17g**–**l**).

Substituted benzenesulphonamides were synthesised from the reaction of various l-amino acids and substituted benzenesulphonyl chlorides in aqueous medium. Base mediated reactions of the benzenesulphonamides (**13a**–**f**) with benzoyl chloride afforded the N-benzoylated benzenesulphonamides (**15a**–**f**). Further reactions of compounds **15a**–**f** and **13g**–**l** with 2-amino benzothiazole in the presence of catalytic amount of boric acid afforded the target compounds (**17a**–**l**, Scheme 2) which were characterised using FTIR, NMR, and HRMS (Schemes 3 and 4).

### Spectral characterisation

The FTIR spectra of the carboxamides derived from N*-*benzoylated benzenesulphonamides (**17a**–**f**) showed one N–H band between 3415 and 3347 cm^−1^; two bands between 1727 and 1654 cm^−1^ assigned to the C=O of the new carboxamides and benzoyl amide. The C=N band appeared at 1640–1613 cm^−1^. The NO_2_ bands appeared at 1525–1496 cm^−1^. These bands were diagnostic of successful coupling of the benzothiazoles with the N-benzoylbenzenesulphonamides.

In the proline derivatives (**17g**–**l**), the OH bands in compounds **17g**, **17i**, and **17k** appeared between 3389 and 3316 cm^−1^. The NH band of **17g**–**l** appeared at 3432–3412 cm^−1^. Only one carbonyl band appeared at 1750–1692 cm^−1^ for each of the derivatives; the C=N appeared at 1647–1619 cm^−1^. The NO_2_ bands appeared at 1528–1495 cm^−1^. The FTIR bands assigned showed successful coupling of the proline derived benzenesulphonamides with 2-aminobenzothiazole.

In the proton NMR, the appearance of the four aromatic protons of the benzothiazole ring between 7.91 and 7.03 ppm is very supportive of the targeted products formation.

The carbon-13 NMR showed all the peaks expected of successful coupled products. The C=N peak appeared between 168.74 and 150.56 ppm. All the carbonyl, aromatic and aliphatic peaks were accounted for in the carbon-13 NMR.

The high resolution mass spectrometer (HRMS) peak of the derivatives appeared either as molecular ions (M^+^) or M + H^+^, M–H^–^, M + H_3_O^+^, or M + Na^+^ adduct. The results corresponded to three decimals with the calculated values. The spectra used for the characterisation of the new compounds are available as supporting materials.

### Molecular docking

#### Validation of the docking procedure

**Figure 1.**
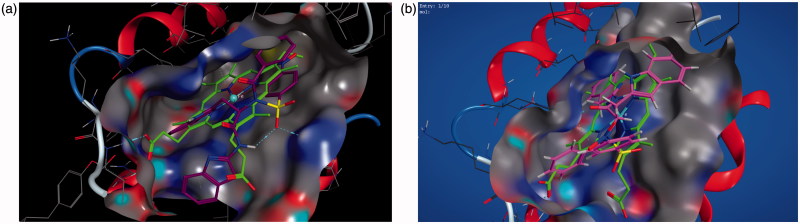
(a, b) Validation of the binding sites of the targets: 1EQG and 1CX2, respectively. The compound in green colour is the co-crystallised ligand of the target, the purple colour is compound 17 fitting into the binding cavity of the target as the native ligand.

**Figure 2.**
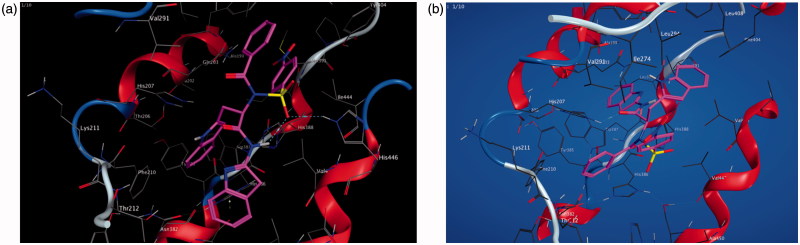
(a, b) Binding interactions of the amino acid residues of the proteins – 1EQG and 1CX2, respectively, with compound **17c**.

**Figure 3.**
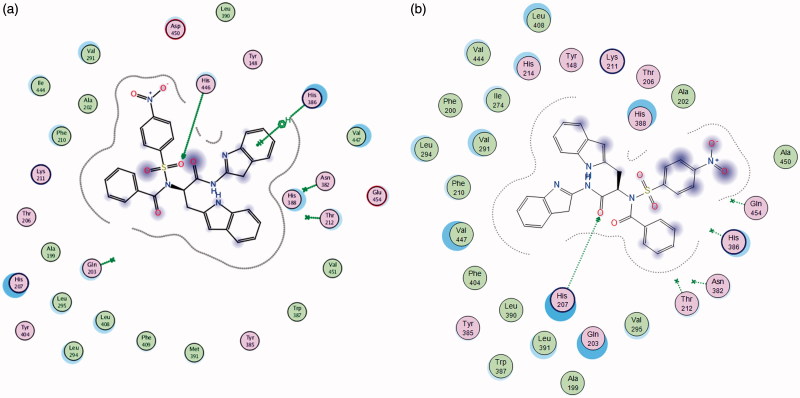
(a, b) The 2D ligand interactions of the amino acid residues of the proteins – 1EQG and 1CX2, respectively, with compound **17c**.

Molecular operating environment (MOE) was used for docking studies. In order to validate the accuracy of MOE-Dock program, the co-crystallised ligand and best performing compound **17c** (in terms of binding affinity, Table 1) were docked simultaneously to the active sites of 1CX and 1EQG. The compounds fitted very well in the binding cavity as the co-crystallised ligand (Figure 1(a,b)). In this study, RMSD value was found as 1.8032 Å and 0.3482 for 1CX2 and 1EQG, respectively, showing that our docking method is valid for the studied inhibitors which make the MOE-Dock method reliable for docking of these compounds. In Figure 1(a,b), the green structure represents the co-crystallised ligand while the purple structures represent the docked ligand. The 12 synthesised compounds were docked into the active binding sites of 1CX2 and 1EQG with their respective binding energy shown in Table 1. Compound **17c** gave the highest binding energy with the two targets used. Therefore, we further analysed the binding modes of **17c** with the targets (Figure 2(a,b)) with a view to understanding the nature and type of interaction involved in the protein–ligand complexes. Figure 3(a,b) depicts the 2D ligand interaction with receptors. There are 28 active amino acid residues (Figure 3(a)) found in the active binding site of 1EQG-17c complex namely: Tyr 148, Tyr 385, Tyr 404, Val 291, Val 447, Val 451, His 207, His 386, His 388, His 446, Lys 211, Thr 206, Thr 212, Gln 203, Glu 454, Ala 199, Ala 202, Met 391, Ile 444, Phe 210, Phe 409, Asn 382, Asp 450, Leu 294, Leu 295, Leu 390, Leu 408, and Trp 387. 1CX2–17c complex contains 28 active amino acid residues: Tyr 148, Tyr 385, Val 291, Val 295, Val 444, Val 447, His 207, His 214, His 386, His 388, Lys 211, Thr 206, Thr 212, Gln 203, Gln 454, Ala 199, Ala 202, Ala 450, Ile 274, Phe 200, Phe 210, Phe 404, Asn 382, Leu 294, Leu 390, Leu 391, Leu 408, and Trp 387 (Figure 3(b)).

**Table 1.** Binding energy of different compounds with the receptor, 1CX2.

Comp	**17a**	**17b**	**17c**	**17d**	**17e**	**17f**	**17g**	**17h**	**17i**	**17j**	**17k**	**17l**
Δ*G* (kcal/mol)	11.30	9.20	12.50	8.27	9.07	9.53	8.80	8.30	9.60	7.97	8.30	8.07

**Table 2.** Anti-inflammatory activities.

	Percentage inhibition of oedema formation			
Compd. no.	0.5 h	1 h	2 h	3 h
**17a**	32.31	7.06	7.03	12.96
**17b**	49.23	42.35	43.75	38.89
**17c**	52.31	72.01	76.36	80.09
**17d**	26.15	5.88	9.38	16.05
**17e**	27.62	17.65	14.84	1.85
**17f**	30.77	18.82	20.31	21.60
**17g**	27.69	7.06	8.59	16.05
**17h**	24.62	23.53	0.78	2.47
**17i**	41.54	64.04	73.02	78.12
**17j**	27.69	16.47	14.84	13.58
**17k**	26.15	5.88	8.28	16.67
**17l**	13.85	2.35	8.59	13.58
Indomethacin	56.92	63.53	64.84	63.58

### Biological studies

#### Anti-inflammatory activities

The results of the anti-inflammatory activity (Table 2) show that all compounds except **17c** and **17i** caused less than 50% reduction of oedema at 1 h, 2 h, and 3 h. The most pronounced anti-inflammatory activity among the compounds studied was **17c**. The percentage reduction of **17c** and **17i** was higher than that of indomethacin at 1 h, 2 h, and 3 h.

The structure–activity relationship (SAR) showed that the indole ring of compound **17c** was more effective in reducing oedema than benzene ring of **17b**. Among the prolines (**17g**–**l**), compound **17i** was the most active derivative possessing anti-inflammatory activity better than indomethacin. Substitution at the four-position of the proline was shown to enhance anti-inflammatory activity. The trend of the anti-inflammatory activity showed that compound **17g**>**17h**, **17i**>**17j**, and **17k**>**17n**. Compound **17a** was the most active derivative among the aliphatic amino acid derivatives (**17a**, **17d**–**f**), indicating that the higher the alkyl group β to the carboxamide, the lower the activity. The trend observed was **17a**>** 17f**>** 17e**>**17d**. The presence of an electron withdrawing group at the para position of the benzenesulphonamide decreased the anti-inflammatory activity as evident with compounds **17g** and **17h** being lower than that of **17i** and **17j**. However, electron withdrawing groups still showed better activity than unsubstituted ring, **17g**–**h**>**17k**–**l** showing that the presence of a substituent at the four-position of the benzene ring enhanced anti-inflammatory activity.

All the compounds evaluated showed analgesic activities (Table 3) but only compounds **17c**, **17g**, **17i**, and **17k** where comparable with celecoxib. The highest analgesic activity was recorded for compound **17i** with ED_50_=72 and 69 µM/kg when compared with celecoxib (ED_50_ 72 and 70 µM/kg) after 1 h and 2 h, respectively. The SAR revealed that all the derivatives that showed good analgesic activities possessed either an additional OH (4-hydroxyprolines) or NH (tryptophan) group. Among the 4-hydroxyproline derivatives, compound **17i** possessed the highest analgesic activity indicating the importance of electron donating CH_3_ para to the benzenesulphonamide. The analgesic activity of compound **17g** was more than that of compound **17k**, indicating the importance of substitution at the para position of the benzenesulphonamide ring in enhancing analgesic activities.

**Table 3.** Analgesic activity (µM/kg), ulcerogenic index and cyclooxygenase inhibition (%).

	Mean number of writhes ± SEM				Cyclooxygenase inhibition (%)		
Compd. no.	0.5 h	1 h	2 h	Ulcer index	COX-1 (%)	COX-2 (%)	S I
**17a**	260 ± 1.6	172 ± 2.1	153 ± 1.8	–	–	–	–
**17b**	172 ± 3.5	421 ± 4.2	245 ± 4.6	–	–	–	–
**17c**	96 ± 1.8	102 ± 1.2	89 ± 2.3	0.82	3.50 ± 0.81	89.20 ± 0.68	25.49
**17d**	304 ± 1.2	275 ± 6.5	201 ± 2.3	–	–	–	–
**17e**	167 ± 0.9	183 ± 1.8	174 ± 2.1	–	–	–	–
**17f**	203 ± 3.2	189 ± 2.9	192 ± 2.3	–	–	–	–
**17g**	127 ± 0.9	84 ± 1.2	76 ± 6.8	–	–	–	–
**17h**	210 ± 1.3	187 ± 2.2	129 ± 6.3	–	–	–	–
**17i**	84 ± 4.3	72 ± 5.3	69 ± 7.2	0.89	2.60 ± 0.72	78.50 ± 0.90	30.19
**17j**	166 ± 2.9	182 ± 3.2	178 ± 2.3	–	–	–	–
**17k**	108 ± 1.1	97 ± 1.2	80 ± 8.2	–	–	–	–
**17l**	182 ± 2.2	195 ± 1.5	186 ± 3.6	–	–	–	–
Celecoxib^29^	156 ± 4.8	72 ± 1.2	70 ± 3.9	0.92	2.20 ± 0.89	61.10 ± 0.88	27.77

### Ulcerogenic activity

Compounds **17c** and **17i** that showed anti-inflammatory activity comparable to indomethacin were subjected to ulcerogenic activity against celecoxib as reference drug (Table 3). The two derivatives (**17c** and **17i**) showed good ulcer index (0.82 and 0.89) respectively when compared with that of celecoxib (0.92).

### *In vitro* COX inhibitory activity

Percentage inhibition of COX-1 and COX-2 and selectivity (COX-2/COX-1) of the most active derivatives at concentration of 2.0 µM are shown in Table 3. Compound **7c** and **7i** showed good selectivity towards COX-2. This indicates that the mechanism of action of test compounds could be COX-2 inhibition.

Physicochemical properties of compounds have been used for over a century to predict or estimate pharmacokinetic properties^32^. Drug-likeness has also been used as a parameter for predicting the balance among the molecular properties of a compound that influences its pharmacodynamics and pharmacokinetic properties. The absorption, distribution, metabolism, and excretion of drug molecules in human body could be optimised using the results of the physicochemical properties.

Lipophilicity is a property that has a major effect on solubility, absorption, distribution, metabolism, and excretion properties as well as pharmacological activity. Highly lipophilic molecules will partition into the lipid interior of membranes and retain there. When log *P* is higher than the upper limit, the drug molecule will have low solubility whereas in lower log *P*, the drug has difficulty to penetrate the lipid membranes^33^. The pharmacokinetics results (Table 3) showed that all the compounds reported have good balance between compound solubility and its penetration of the lipid bilayers.

The empirical conditions to satisfy Lipinski’s rule and manifest a good oral bioavailability involve a balance between the aqueous solubility of a compound and its ability to diffuse passively through the different biological barriers^34^. Reckitt reported the modified Lipinski’s rule of 5 (ro5), stating that a likely drug molecule should have an octanol-water partition coefficient (log *P*) between –0.4 and 5.6, molar refractivity (AMR) between 40 and 130, number of atoms (nA) between 20 and 70, hydrogen bond donor (HBD) ≤ 5 taken as equivalent to the number of –OH and –NH groups, hydrogen bond acceptor (HBA) ≤ 10 taken as equivalent to the number of oxygen and nitrogen atoms and molecular weight (MW) not more than 500. A violation of more than one of these physicochemical parameter disqualifies a compound from being a likely drug. However, compounds that will serve as substrate for biological transporters do not obey this rule. They can have violations up to 4^35^. This then imply that violations of more than one rule do not totally rule out a compound as a likely drug candidate. A check on the results (Table 4) showed that all the reported derivatives are likely drugs with respect to Lipinski’s rule of five. The modified Lipinski’s ro5 reported by Reckitt showed that compounds **17b**–**f** had two violations and are not likely drug candidate. Verber et al.^36^ reported that the number of rotatable bond (nRB) influences bioavailability in rats and recommended NRB ≤10 for good oral bioavailability. Of all the compounds reported herein, only compounds **17b**–**e** had NRB of 11 suggesting that they may have oral bioavailability problems. Total polar surface area (TPSA) has often been used as a surrogate property for cell permeability. A molecule with TPSA ≤140 Å^2^ would be able to permeate the cell. Only compounds **17g**–**l** had TPSA less than 140 and as such can permeate the cell membranes. The percentage solubility calculated from % ABS = 109–0.345 × TPSA^37^, showed that only compounds **17g**–**l** had good solubility at 74% which is a designation of good bioavailability upon oral administration.

**Table 4.** Pharmacokinetics of the new derivatives.

S/N	MW	log *P*	HBA	HBD	TPSA	AMR	nRB	nA	nAc	ABS %	LNV	RNV
**17a**	497	0.648	7	0	160.7	134.93	9	34	0	53.8	0	1
**17b**	586	2.423	7	0	160.7	168.29	11	41	0	53.8	1	2
**17c**	625	2.03	8	0	160.7	180.15	11	44	0	53.8	1	2
**17d**	552	2.609	7	0	160.7	152.41	11	38	0	53.8	1	2
**17e**	552	2.398	7	0	160.7	151.03	11	38	0	53.8	1	2
**17f**	538	1.829	7	0	160.7	147.96	10	37	0	53.8	1	2
**17g**	449	–0.62	7	0	143.63	112.6	6	30	0	59.45	0	0
**17h**	432	0.406	6	0	143.63	109.55	6	29	0	59.45	0	0
**17i**	417	–0.24	7	0	100.49	113.66	5	28	0	74.33	0	0
**17j**	401	0.792	6	0	100.49	110.61	5	27	0	74.33	0	0
**17k**	403	–0.63	7	0	100.49	109.38	5	27	0	74.33	0	0
**17l**	387	0.40	6	0	100.49	106.33	5	26	0	74.33	0	0

MW: molecular weight; HBA: hydrogen bond acceptor; HBD: hydrogen bond donor; TPSA: total polar surface area; nRB: number of rotatable bond; nAc: number of acid; AMR: molar refractivity; nA: number of atoms; LNV: Lipinski’s number of violations; RNV: Reckitt’s number of violation.

The BBB likeness states that a CNS drug must have HBA value in the range of 8–10, the MW must be in the range of 400–500 and the number of acid must be zero. All the synthesised compounds reported herein fulfilled the conditions and as such can cross the blood brain barrier and might be useful as to treat brain inflammations.

## Conclusions

In this paper, we have described an efficient, ecofriendly, and versatile approach to obtain substituted benzenesulphonamides bearing benzothiazole and carboxamide. All the compounds were evaluated for their anti-inflammatory and analgesic activities. Two of the derivatives **7c** and **7i** were found to possess anti-inflammatory activities comparable with indomethacin. Five of the reported new derivatives were also found to possess analgesic activities comparable with celecoxib. The ulcerogenic activity showed the two most potent derivatives **7c** and **7i** to be comparable with celecoxib. The SAR revealed that substitution at the para position of the benzenesulphonamide and presence of electron rich NH or OH group enhanced the anti-inflammatory and analgesic properties. The pharmacokinetics calculations showed that the reported derivatives would not have oral bioavailability, transport and permeability problems.

## Supplementary Material

IENZ_1426573_Supplementary_Material.pdf
