# Supplementary material for: Novel anti-inflammatory and analgesic agents: synthesis, molecular docking and in vivo studies
Source: J Enzyme Inhib Med Chem. 2018 Jan 26;33(1):405–15. doi: 10.1080/14756366.2018.1426573 (PMC7011796; doi:10.1080/14756366.2018.1426573)

Supporting document

2-(4-methylphenylsulphonamido) acetic acid (3a)

The amino acid was glycine, yield (2.8410 g, 99.34%), mp, 88.4-88.6 ^{o}C, FTIR (KBr, cm^{-1}): 3448 (OH of COOH), 3277 (NH), 2957 (C-H aliphatic), 1730 (C=O), 1598, 1440 (C=C), 1354, 1321 (2S=O), 1185 (SO_2-NH), 1111, 1094 (C-N, C-O). ^{1}H NMR (500 MHz, DMSO-d_6)δ: 7.89 (t, J=6.3 Hz, 1H, NH), 7.64 (d, J=8.6 Hz, 2H, ArH), 7.33 (d, J=8.05 Hz, 2H, ArH), 3.51 (d, J=5.7 Hz, CH_2), 2.34 (s, 3H, CH_3). ^{13}C NMR (500 MHz, DMSO-d_6)δ: 170.7 (C=O), 143.1, 138.4, 130.0, 127.1 (aromatic carbons), 44.3 (CH_2), 21.5 (CH_3). HRMS-ESI (m/z): 228.0410 $(M-H)^-$, calculated, 228.0408.

2-(4-methylphenylsulphonamido)-3-phenylpropanoic acid (3b)

The amino acid was L-phenylalanine, yield (3.9848 g, 99.93%), mp, 135.10 ^{o}C FTIR (KBr, cm^{-1}): 3439 (OH of COOH), 3322 (NH), 3025 (C-H aromatic), 2926 (C-H aliphatic), 1713 (C=O), 1597, 1496, 1456 (C=C), 1344, 1331 (2S=O), 1189, 1157 (SO_2NH), 1136, 1090, 1052 (C-N, C-O). ^{1}H NMR (DMSO-d_6)δ: 8.12 (d, J= 9.15 Hz, 1H, NH), 7.46 (d, J=7.45 Hz, 2H, ArH), 7.41 (d, J= 8.6 Hz, 2H, ArH), 7.21-7.06 (m, 5H, ArH), 3.81 (dd, J= 3.45, 2.85 Hz, 1H, CH), 2.88 (dd, J= 5.75, 5.75 Hz, 1H, CH_a of CH_2), 2.67 (dd, J=8.6, 8.6 Hz, 1H, CH_b of CH_2), 2.31-2.29 (m, 3H, CH_3). ^{13}C NMR (DMSO-d_6, 500 MHz)δ: 172.8 (C=O), 142.8, 138.7, 137.3, 129.7, 128.7, 126.9, 126.8, 126.0 (eight aromatic carbons), 57.9, 38.0, 21.5 (three aliphatic carbons). HRMS-ESI (m/z): 320.0958 $(M+H)^+$, calculated, 320.0951

3-(1*H*-indol-2-yl)-2-{[(4-methylphenyl)sulphonyl]amino}propanoic acid (3c)

The amino acid was L-tryptophan, yield (4.3814 g, 97.880%), FTIR (KBr, cm^{-1}): 3385, 3302 (2NH), 2921, 2858 (C-H aliphatic), 1750 (C=O), 1618, 1598, 1494, 1457, 1429 (C=C), 1321, 1291 (2S=O), 1163, 1131 (SO_2NH), 1082, 1019 (C-N, C-O). ^{1}H NMR (DMSO-d_6) δ: 10.73 (s, 1H, NH of indole), 8.07 (d, J= 8.60 Hz, 1H, NH of SO_2NH), 7.67 (d, J= 8.05 Hz, 1H, ArH), 7.42 (d, J= 8.60 Hz, 2H, ArH), 7.33 (d, J= 8.00 Hz, 1H, ArH), 7.25 (t, J= 8.05 Hz, 1H, ArH), 7.13 (d, J= 8.00 Hz, 2H, ArH), 7.02-6.99 (m, 2H, ArH), 6.90-6.87 (m, 1H, ArH), 3.84 (dd, J= 7.45, 8.00 Hz, 1H, CH), 3.00 (dd, J= 6.3, 6.90 Hz, 1H, CH_a, CH_2), 2.80 (dd, J= 7.45, 7.45 Hz, 1H, CH_b of CH_2), 2.28 (s, 3H, CH_3-Ar). ^{13}C NMR (DMSO-d_6) δ: 173.1 (C=O), 142.7, 138.5, 136.6, 129.6, 127.4, 126.8, 124.4, 121.3, 118.8, 118.3, 111.9, 109.4 (aromatic carbons), 66.9, 57.1, 21.5 (aliphatic carbons). HRMS-ESI (m/z): 359.1060 $(m+H)^+$, calculated 359.1057

4-Methyl-2-{[(4-methylphenyl)sulfonyl]amino}pentanoic acid (3d)

The amino acid was L-leucine, yield (3.502 g, 98.26%), mp, 114.10 ^{o}C. FTIR (KBr, cm^{-1}): 3423 (OH of CO_2H), 3279 (NH), 2949 (C-H aromatic), 2872 (C-H aliphatic), 1706 (C=O), 1598, 1497, 1458, 1420 (C=C), 1384, 1339 (2S=O), 1168, 1152 (SO_2NH), 1122, 1091, 1020 (C-N, C-O). ^{1}H NMR (DMSO-d_6) δ: 7.97 (d, J= 8.60 Hz, 1H, NH), 7.61 (d, J= 8.55 Hz, 2H, Ar-H), 7.31 (d, J= 8.55 Hz, 2H, ArH), 3.60 (m, 1H, CH-CO_2H), 2.33 (s, 3H, CH_3-Ar), 1.55-1.50 (m, 1H, CH), 1.36-1.32 (m, 2H, CH_2), 0.77-0.76 (d, J= 6.85 Hz, 3H, CH_3), 0.65 (d, J= 6.30 Hz, 3H, CH_3). ^{13}C NMR (DMSO-d_6) δ: 173.8 (C=O), 142.9, 138.9, 129.9, 128.6, 127.0, 126.0 (aromatic carbons), 54.5, 41.5, 24.4, 23.1, 21.6 (aliphatic carbons). HRMS-ESI (m/z): 286.1986 $(M+H)^+$, calculated 286.1988.

3-methyl-2-{[(4-methylphenyl)sulfonyl]amino}pentanoic acid (3e)

The amino acid was L-isoleucine, yield (3.412 g, 95.74%), mp, 130 ^{o}C, FTIR (KBr, cm^{-1}): 3280 (NH), 2970, 2934 (C-H Ar-H), 2883 (C-H aliphatic), 1710 (C=O), 1599, 1496, 1460 (C=C), 1385, 1334 (2S=O), 1185, 1161 (SO_2NH), 1092, 1058, 1020 (C-N, C-O). ^{1}H NMR (DMSO-d_6) δ: 7.87 (d, J= 9.2 Hz, 1H, NH), 7.61 (d, J= 8.05 Hz, 2H, ArH), 7.30 (d, J= 8.05 Hz, 2H, ArH), 3.49 (dd, J= 6.30, 6.30 Hz, 1H, CH-CO_2H), 2.33 (s, 3H, CH_3-Ar), 1.62-1.58 (m, 1H, CH), 1.34-1.29 (m, 1H, CH_a of CH_2), 1.09-1.01 (m, 1H, CH_b of CH_2), 0.72 (dt, J= 6.85, 7.45H, 6H, $2CH_3$). ^{13}C NMR (DMSO-d_6) δ: 172.7 (C=O), 142.9, 138.9, 129.8, 127.1 (aromatic carbons), 60.5, 37.4, 24.9, 21.5, 15.9, 11.4 (aliphatic carbons). HRMS-ESI (m/z): 284.0765 $(m-H)^-$, calculated 284.0765.

3-Methyl-2-(4-methylphenylsulphonamido)butanoic acid (3f)

The amino acid was L-valine, yield (3.2849 g, 95.56%), mp, 121.9 ^{o}C, FTIR (KBr, cm^{-1}): 3293 (NH), 2970 (C-H aliphatic), 1708 (C=O), 1598, 1466, 1419 (C=C), 1333, 1289 (2S=O), 1161 (SO_2NH), 1089 (C-N or C-O). ^{1}H NMR (DMSO-d_6) δ: 7.86 (d, J=9.75 Hz, 1H, NH), 7.68-7.61 (m, 2H, ArH), 7.33-7.22 (m, 2H, ArH), 3.45 (dd, J= 5.70, 5.75Hz, 1H, CH-CO_2H), 2.33-2.30 (m, 3H, CH_3-Ar), 1.90-1.86 (m, 1H, CH), 0.78-0.64 (m, 6H, $2CH_3$). ^{13}C NMR (DMSO-d_6) δ: 172.7 (C=O), 142.9, 138.9, 129.8, 127.1, 126.2 (aromatic carbons), 61.7, 30.9, 21.5, 19.5, 18.4 (aliphatic carbons). HRMS-ESI (m/z): 271.0881 (M^+), calculated 271.0882.

4-Hydroxy-1-tosylpyrrolidine-2-carboxylic acid (3g)

The amino acid was L-hydroxyproline, yield (3.564 g, 99.86%), mp, 98.4 °C. FTIR (KBr, cm^{-1}): 3524 (OH), 2931 (C-H aliphatic), 1708 (C=O), 1600, 1444 (C=C), 1347, 1332 (2S=O), 1200, 1158 (SO_2N), 1090, 1075 (C-N or C-O). ^1H NMR (DMSO-d_6) δ: 7.65 (d, J= 8.00 Hz, 2H, ArH), 7.37 (d, J= 8.05 Hz, 2H, ArH), 4.18 (s, 1H, O-H), 4.01 (t, J= 8.05 Hz, 1H, CH-CO_2H), 3.43-3.40 (m, 1H, CH-OH), 3.04 (d, J=10.3 Hz, 2H, CH_2), 2.36 (s, 3H, CH_3), 1.91 (t, 4.6 Hz, 2H, CH_2). ^{13}C NMR (DMSO-d_6) δ: 173.8 (C=O), 143.7, 135.0, 130.1, 127.9 (aromatic carbons), 68.9, 60.2, 56.8, 21.5 (aliphatic carbons). HRMS-ESI (m/z): 286.1097 $(m+H)^+$, calculated 286.1099.

1-Tosylpyrrolidine-2-carboxylic acid (3h)

The amino acid was L-proline, yield (3.2989 g, 98.09%), mp, 50.4°C, FTIR (KBr, cm^{-1}): 3415 (OH of COOH), 2957 (C-H aliphatic), 1737 (C=O), 1619, 1597, 1494, 1449 (C=C), 1345, 1306 (2S=O), 1199 (SO_2N), 1159, 1095, 1013 (C-N, C-O). ^1H NMR (DMSO-d_6) δ: 7.65 (d, J=8.6 Hz, 2H, ArH), 7.35 (d, J= 8.00 Hz, 2H, ArH), 4.04 (dd, J= 4.55, 5.15 Hz, 1H, CH-COOH), 3.29 (dd, J= 9.75, 5.15Hz, 1H, CH_a of CH_2-N), 3.08 (dd, J= 6.85, 7.45 Hz, 1H, CH_b of CH_2-N), 2.31 (s, 3H, CH_3-Ar), 1.81-1.72 (m, 3H), 1.48 (t, J=5.15 Hz, 1H). ^{13}C NMR (DMSO-d_6) δ: 173.7 (C=O), 143.9, 135.1, 130.3, 127.6 (aromatic carbons), 66.9, 48.9, 30.9, 24.7, 21.4 (aliphatic carbons). HRMS-ESI (m/z): 269.0726 $(m+H)^+$, calculated 269.0728.

2-Benzenesulphonamido acetic acid (3i)

The amino acid was glycine, yield (2.6856 g, 99.61%), mp, 170.80 °C. FTIR (KBr, cm^{-1}): 3317 (NH), 3060, 2974 (C-H aromatic), 2946 (C-H aliphatic), 1728 (C=O), 1587, 1451, 1428, 1412 (C=C), 1318, 1247 (S=O), 1158, 1130 (SO_2NH), 1095, 1077, 1012 (C-N, C-O). ^1H NMR (DMSO-d_6) δ: 8.00 (t, J= 12.00 Hz, 1H, NH), 7.76 (d, J= 7.45 Hz, 2H, ArH), 7.59 (t, J= 7.45 Hz, 1H, ArH), 7.54 (t, J= 8.00 Hz, 2H, ArH), 3.35 (d, J= 6.30 Hz, 2H, CH_2). ^{13}C NMR (DMSO-d_6) δ: 170.7 (C=O), 141.2, 132.9, 129.6, 126.9 (aromatic carbons), 44.5 (aliphatic carbon). HRMS-ESI (m/z): 216.1252, calculated 216.1255.

2-Benzenesulphonamido-3-phenylpropanoic acid (3j)

The amino acid was L-phenylalanine, yield (3.8169 g, 100%), mp, 129.10 °C. FTIR (KBr, cm^{-1}): 3342 (NH), 3195, 3029 (C-H aromatic), 2968 (C-H aliphatic), 1735 (C=O), 1697, 1496, 1447 (C=C), 1375, 1347 (2S=O), 1170, 1108 (SO_2NH), 1093, 1028 (C-N, C-O). ^1H NMR (DMSO-d_6)

δ: 8.25 (d, J= 8.85 Hz, 1H, NH), 7.54-7.50 (m, 3H, ArH), 7.40 (t, J= 7.45 Hz, 2H, ArH), 7.18-7.12 (m, 3H, ArH), 7.08 (d, J= 7.15 Hz, 2H, ArH), 3.84 (ddd, J= 6.00, 5.75, 6.30 Hz, 1H, CH-CO_2H), 2.90 (dd, J= 5.75, 5.75 Hz, 1H, CH_a of CH_2), 2.67 (dd, J= 9.00, 9.00 Hz, 1H, CH_b of CH_2). ^{13}C NMR (DMSO-d_6) δ: 172.8 (C=O), 141.6, 137.3, 132.6, 129.7, 129.4, 128.7, 127.1, 126.7 (aromatic carbons), 57.9, 38.3 (aliphatic carbons). HRMS-ESI (m/z): 304.0624 $(M-H)^-$, calculated 304.0627.

2-Benzenesulphonamido-3-(1*H*-indol-3-yl)propanoic acid (3k)

The amino acid was L-tryptophan, yield (4.2805 g, 99.43%), mp, 106.40 ^oC. FTIR (KBr, cm^{-1}): 3366, 3311 (2NH), 3061 (C-H aromatic), 2936 (C-H aliphatic), 1746 (C=O), 1619, 1550, 1451, 1430 (C=C), 1323, 1235 (2S=O), 1214, 1160 (SO_2NH), 1127, 1091, 1012 (C-N, C-O). ^1H NMR (DMSO-d_6) δ: 10.78 (s, 1H, NH of indole), 8.22 (d, J= 8.60 Hz, 1H, NH), 7.59-7.57 (m, 2H, ArH), 7.48 (t, J= 6.85 Hz, 1H, ArH), 7.38-7.35 (m, 2H, ArH), 7.27 (d, J= 8.60 Hz, 2H, ArH), 7.03-6.99 (m, 2H, ArH), 6.90 (t, J= 7.40 Hz, 1H, ArH), 3.91-3.87 (m, 1H, ArH), 3.05-3.01 (dd, J= 6.85, 6.85 Hz, 1H, CH_a of CH_2), 2.86-2.81 (dd, J= 8.05, 7.45 Hz, 1H, CH_b of CH_2). ^{13}C NMR (DMSO-d_6) δ: 173.1 (C=O), 141.5, 136.6, 132.6, 129.2, 127.5, 126.7, 124.4, 121.4, 118.9, 118.4, 111.9, 109.4 (aromatic carbons), 57.2, 28.8 (aliphatic carbons). HRMS-ESI (m/z): 344.0835 (M^+), calculated 344.0831.

2-Benzenesulphonamido-3-methylpentanoic acid (3l)

The amino acid was L-isoleucine, yield (3.1052 g, 91.55%), mp, 148.00 ^oC. FTIR (KBr, cm^{-1}): 3295 (NH), 2968 (C-H aromatic), 2936, 2883 (C-H aliphatic), 1699 (C=O), 1585, 1450, 1416 (C=C), 1340, 1384 (2S=O), 1168 (SO_2NH), 1092, 1026 (C-N, C-O). ^1H NMR (DMSO-d_6, 400 MHz) δ: 8.00 (d, J= 9.16 Hz, 1H, NH), 7.72 (d, J= 8.60 Hz, 2H, ArH), 7.58-7.49 (m, 3H, ArH), 3.50 (dd, J= 7.45, 7.45 Hz, 1H, CH-CO_2H), 1.62-1.57 (m, 1H, CH), 1.33-1.27 (m, 1H, CH_a of CH_2), 1.07-0.99 (m, 1H, CH_b of CH_2), 0.75-0.68 (m, 6H, $2CH_3$). ^{13}C NMR (DMSO-d_6, 400MHz) δ: 172.7 (C=O), 141.6, 132.8, 129.4, 127.0 (aromatic carbons), 60.5, 37.4, 24.9, 15.9, 11.4 (aliphatic carbons). HRMS-ESI (m/z): 271.0879 (M^+), calculated, 271.0878.

2-Benzenesulphonamido-3-methylbutanoic acid (3m)

The amino acid was L-valine, yield (3.2030 g, 99.68%), mp, 143.60 °C. FTIR (KBr, cm^{-1}): 3418 (OH CO_2H), 3302 (NH), 2969 (C-H aliphatic), 1703 (C=O), 1585, 1451 (C=C), 1338, 1294 (2S=O), 1169, 1143 (SO_2NH), 1093, 1042 (C-N, C-O). ^1H NMR (DMSO-d_6, 400MHz) δ: 7.98 (d, J= 9.16 Hz, 1H, NH), 7.73 (d, J= 6.88 Hz, 2H, ArH), 7.58-7.48 (m, 3H, ArH), 3.46 (dd, J= 6.44, 5.96 Hz, 1H, CH-CO_2H), 1.92-1.84 (m, 1H, CH), 0.77-0.73 (m, 6H, CH_3). ^{13}C NMR (DMSO-d_6, 400MHz) δ: 172.7 (C=O), 141.7, 132.8, 129.4, 127.0 (aromatic carbons), 61.8, 30.9, 19.5, 18.3 (aliphatic carbons). HRMS-ESI (m/z): 258.1824 (M+H), calculated, 258.1829.

1-(Benzenesulphonyl)-4-hydroxypyrrolidine-2-carboxylic acid (3n)

The amino acid was L-4-hydroxyproline, yield (3.3815 g, 99.99%), mp, 159.00 °C. FTIR (KBr, cm^{-1}): 3402 (OH), 2993, 2955 (C-H aliphatic), 1714 (C=O), 1589, 1484, 1450 (C=C), 1385, 1353 (2S=O), 1195, 1158 (SO_2NH), 1158, 1100, 1070 (C-N, C-O). ^1H NMR (DMSO-d_6, 400MHz) δ: 7.77 (d, J= 7.32 Hz, 2H, ArH), 7.63 (t, J= 7.36 Hz, 1H, ArH), 7.55 (t, J= 7.80 Hz, 2H, ArH), 4.18 (s, 1H, OH), 4.03 (t, J= 7.80 Hz, 1H, CH-CO_2H), 3.45-3.41 (m, 1H, CHOH), 3.09 (d, J= 11.00 Hz, 2H, CH_2-N), 1.98-1.87 (m, 2H, CH_2). ^{13}C NMR (DMSO-d_6, 400MHz) δ: 173.8 (C=O), 137.8, 133.5, 129.6, 127.9 (aromatic carbons), 68.9, 60.2, 56.8, 31.2 (aliphatic carbons). HRMS-ESI (m/z): 271.0518 (M^+), calculated, 271.0514.

1-(Benzenesulphonyl)-pyrrolidine-2-carboxylic acid (3o)

The amino acid was L-proline, yield (3.1911 g, 100%). FTIR (KBr, cm^{-1}): 3066 (C-H aromatic), 2983, 2884 (C-H aliphatic), 1730 (C=O), 1627, 1447 (C=C), 1343, 1292 (2S=O), 1199, 1161 (SO_2NH), 1095, 1073, 1016 (C-N, C-O). ^1H NMR (DMSO-d_6, 400MHz) δ: 7.83-7.78 (m, 2H, ArH), 7.67-7.63 (m, 1H, ArH), 7.59-7.56 (m, 2H, ArH), 4.07 (dd, J= 4.60, 4.60 Hz, 1H, CH-CO_2H), 3.34-3.28 (m, 1H, CH_a of CH_2N), 3.14-3.08 (m, 1H, CH_b of CH_2N), 1.86-1.71 (m, 3H), 1.53-1.47 (m, 1H). ^{13}C NMR (DMSO-d_6, 400MHz) δ: 173.7 (C=O), 137.7, 133.6, 129.9, 127.6 (aromatic carbons), 60.9, 48.9, 30.9, 24.7 (aliphatic carbons). HRMS-ESI (m/z): 256.0563 (M+H), calculated 256.0565.

{Benzoyl[(4-methylphenyl)sulfonyl]amino}acetic acid (5a)

Yield (0.3281 g, 98.49%), mp, 104.60 °C, FTIR (KBr, cm^{-1}): 3072 (C-H aromatic), 1729, 1687 (2C=O), 1601, 1583, 1453, 1423 (C=C), 1327, 1293 (2S=O), 1157, 1186 (SO_2N), 1128, 1094, 1073, 1001 (C-N, C-O). ^1H NMR (DMSO-d_6, 500MHz) δ: 7.64 (d, J=8.6 Hz, 2H, ArH), 7.57-

7.43 (m, 5H, ArH), 7.33 (d, J=8.05 Hz, 2H, ArH), 3.51 (d, J=5.7 Hz, CH_2), 2.34 (s, 3H, CH_3). ^{13}C NMR (DMSO-d_6, 500MHz) δ: 170.7, 167.8 (2C=O), 143.1, 138.4, 133.4, 131.3, 130.0, 129.8, 129.1, 127.1 (aromatic carbons), 44.3, 21.5 (aliphatic carbons). HRMS-ESI (m/z): 333.0679 (M^+), calculated, 333.0671.

2-[*N*-(4-methylbenzenesulfonyl)-1-phenylformamido]-3-phenylpropanoic acid (5b)

Yield (0.4201 g, 99.31%), mp, 100.50 ^{o}C, FTIR (KBr, cm^{-1}): 3443 (OH of CO_2H), 3072 (C-H aromatic), 1732, 1689 (2C=O), 1603, 1585, 1497, 1454, 1425 (C=C), 1344, 1327, (2S=O), 1187, 1168, 1158 (SO_2N), 1128, 1090, 1027 (C-N, C-O). ^{1}H NMR (DMSO-d_6, 500MHz) δ: 7.92-7.91 (m, 3H, ArH), 7.59 (t, J= 7.45 Hz, 2H, ArH), 7.48-7.41 (m, 5H, ArH), 7.20-7.15 (m, 3H, ArH), 7.08 (d, J= 7.45 Hz, 1H, ArH), 3.81 (t, J= 6.30 Hz, 1H, CH-CO_2H), 2.88 (dd, J= 5.75, 5.75 Hz, 1H, CH_a of CH_2), 2.67 (dd, J= 9.15, 8.55 Hz, 1H, CH_b of CH_2), 2.30 (s, 3H, CH_3-Ar). ^{13}C NMR (DMSO-d_6, 500MHz) δ: 172.8, 167.8 (2C=O), 142.8, 138.7, 137.3, 133.4, 133.2, 131.3, 129.8, 129.7, 129.1, 128.7, 126.9, 126.8 (aromatic carbons), 57.9, 38.4, 21.5 (aliphatic carbons). HRMS-ESI (m/z): 424.1150 (M+H), calculated, 424.1142.

3-(1*H*-indol-2-yl)-2-[*N*-(4-methylbenzenesulfonyl)-1-phenylformamido]propanoic acid (5c)

Yield (0.4609 g, 99.74%), mp, 108.60 ^{o}C, FTIR (KBr, cm^{-1}): 3335 (NH), 3072 (C-H aromatic), 1761, 1688 (2C=O), 1619, 1602, 1584, 1454, 1424 (C=C), 1326, 1292 (2S=O), 1178, 1160 (SO_2N), 1128, 1073, 1027 (C-N, C-O). ^{1}H NMR (DMSO-d_6, 500MHz) δ: 10.73 (s, 1H, NH of indole), 7.91 (t, J= 7.45 Hz, 3H, ArH), 7.59 (t, J= 7.45 Hz, 2H, ArH), 7.48-7.42 (m, 5H, ArH), 7.25 (t, J= 8.60 Hz, 1H, ArH), 7.13 (d, J= 8.05 Hz, 1H, ArH), 7.00 (t, J= 7.45 Hz, 1H, ArH), 6.88 (t, J= 7.45 Hz, 1H, ArH), 3.84 (dd, J= 7.45, 8.05 Hz, 1H, ArH), 3.00 (dd, J= 6.30, 6.30 Hz, 1H, CH_a of CH_2), 2.80 (dd, J= 8.05, 8.00 Hz, 1H, CH_b of CH_2), 2.28 (s, 3H, CH_3-Ar). ^{13}C NMR (DMSO-d_6, 500MHz) δ: 173.1, 167.8 (2C=O), 142.7, 138.5, 136.6, 133.4, 131.3, 129.8, 129.6, 129.1, 127.4, 126.8, 124.4, 121.3, 118.8, 118.3, 111.9, 109.4 (aromatic carbons), 57.1, 28.8, 21.5 (aliphatic carbons). HRMS-ESI (m/z): 480.2135 ($M+NH_4$), calculated, 480.2140.

4-Methyl-2-[*N*-(4-methylbenzenesulfonyl)-1-phenylformamido]pentanoic acid (5d)

Yield (0.3889 g, 99.92%), mp, 98.90 ^{o}C, FTIR (KBr, cm^{-1}): 3415 (OH of CO_2H), 2968 (C-H aromatic), 2676 (C-H aliphatic), 1705, 1688 (2C=O), 1619, 1602, 1584, 1454, 1424 (C=C), 1327, 1292 (2S=O), 1161, 1128 (SO_2N), 1091, 1073, 1027 (C-N, C-O). ^{1}H NMR (DMSO-d_6,

500MHz) δ: 7.91 (t, J= 6.90 Hz, 3H, ArH), 7.62-7.57 (m, 2H, ArH), 7.47 (t, J= 8.00 Hz, 3H, ArH), 7.31 (d, J= 8.00 Hz, 1H, ArH), 3.59 (dd, J= 8.05, 8.55 Hz, 1H, CH-CO_2H), 1.55-1.50 (m, 1H, CH), 1.36-1.30 (m, 2H, CH_2), 0.76 (d, J= 6.30 Hz, 3H, CH_3), 0.65 (d, J= 6.30 Hz, 3H, CH_3). ^{13}C NMR (DMSO-d_6, 500MHz) δ: 173.8, 167.8 (2C=O), 142.9, 138.9, 133.4, 131.3, 129.9, 129.8, 129.1, 127.0 (aromatic carbons), 54.5, 41.5, 24.4, 23.1, 21.6, 21.5 (aliphatic carbons). HRMS-ESI (m/z): 390.2354 (M+H), calculated, 390.2358.

3-Methyl-2-[*N*-(4-methylbenzenesulfonyl)-1-phenylformamido]pentanoic acid (5e)

Yield (0.3890 g, 99.95%), mp, 98.90 °C, FTIR (KBr, cm^{-1}): 3415 (OH of CO_2H), 3072, 2970 (C-H of aromatic), 1707, 1688 (2C=O), 1602, 1584, 1496, 1454, 1424 (C=C), 1328, 1292 (2S=O), 1186, 1161 (SO_2N), 1128, 1092, 1073, 1027 (C-N, C-O). ^{1}H NMR (DMSO-d_6, 500MHz) δ: 7.92-7.87 (m, 3H, ArH), 7.62-7.57 (m, 2H, ArH), 7.47 (t, J= 8.00 Hz, 3H, ArH), 7.30 (d, J= 8.00 Hz, 1H, ArH), 3.49 (d, J= 2.3 Hz, 1H, CH-CO_2H), 2.33 (s, 3H, CH_3-Ar), 1.64-1.58 (m, 1H, CH), 1.34-1.27 (m, 1H, CH_a of CH_2), 1.09-1.00 (m, 1H, CH_b of CH_2), 0.72 (dt, J= 6.30, 6.90 Hz, 6H, 2CH_3). ^{13}C NMR (DMSO-d_6, 500MHz) δ: 172.7, 167.8 (2C=O), 142.9, 138.9, 133.4, 131.3, 129.8, 129.8, 129.1, 127.1 (aromatic carbons), 60.5, 37.5, 24.9, 21.5, 15.9, 11.4 (aliphatic carbons). HRMS-ESI (m/z): 388.0894 (M-H), calculated, 388.0899.

3-Methyl-2-[*N*-(4-methylbenzenesulfonyl)-1-phenylformamido]butanoic acid acid (5f)

Yield (0.372 g, 99.20%), mp, 98.90 °C, FTIR (KBr, cm^{-1}): 3415 (OH, CO_2H), 2970 (C-H aliphatic), 1706, 1688 (2C=O), 1618, 1602, 1584, 1454, 1424 (C=C), 1328, 1292 (2S=O), 1185, 1161 (SO_2N), 1127, 1087, 1073 (C-N, C-O). ^{1}H NMR (DMSO-d_6, 500MHz) δ: 7.91 (d, J= 7.45 Hz, 2H, ArH), 7.63-7.57 (m, 4H, ArH), 7.46 (t, J= 8.00 Hz, 2H, ArH), 7.30 (d, J= 8.00 Hz), 3.45 (d, J= 4.60 Hz, 1H, CH-CO_2H), 1.90-1.85 (m, 1H, CH), 0.84-0.70 (m, 6H, CH_3). ^{13}C NMR (DMSO-d_6, 500MHz) δ: 172.7, 167.8 (2C=O), 142.9, 138.9, 133.4, 131.3, 129.8, 129.3, 129.1, 127.1 (aromatic carbons), 61.7, 30.9, 21.5, 19.5, 18.4 (aliphatic carbons). HRMS-ESI (m/z): 375.1148 (M^+), calculated, 375.1140.

2-[*N*-(benzenesulfonyl)-1-phenylformamido]acetic acid (5i)

Yield (0.2999 g, 93.92%), mp, 121.60 °C, FTIR (KBr, cm^{-1}): 3073, 3011 (C-H aromatic), 2837 (C-H aliphatic), 1732, 1688 (2C=O), 1619, 1603, 1584, 1454, 1425 (C=C), 1327, 1293 (2S=O),

1186, 1128 (SO_2N), 1101, 1073, 1027 (C-N, C-O). ^1H NMR (DMSO-d_6, 400MHz) δ: 7.90 (t, J= 8.20 Hz, 4H, ArH), 7.60-7.56 (m, 2H, ArH), 7.45 (t, J= 7.80 Hz, 4H, ArH), 3.54 (s, 2H, CH_2). ^{13}C NMR (DMSO-d_6, 400MHz) δ: 174.7, 167.8 (2C=O) 154.1, 151.1, 133.4, 131.3, 129.8, 129.1, 119.4, 112.8 (eight aromatic carbons), 59.0 (aliphatic carbon). HRMS-ESI (m/z): 320.1628 (M+H), calculated, 320.1631.

2-[*N*-(benzenesulphonyl)-1-phenylformamido]-3-phenylpropanoic acid (5j)

Yield (0.4090 g, 99.90%), mp, 94.10 °C, FTIR (KBr, cm^{-1}): 3342 (OH of COOH), 3073, 3029 (C-H aromatic), 2968 (C-H aliphatic), 1735, 1688 (C=O), 1603, 1584, 1496, 1453, 1425 (C=C), 1376, 1348 (2S=O), 1170, 1108 (SO_2N), 1094, 1073, 1027, 1000 (C-N, C-O). ^1H NMR (DMSO-d_6, 400MHz) δ: 7.91 (d, J=8.00 Hz, 4H, ArH), 7.57 (t, J= 7.45 Hz, 2H, ArH), 7.52 (t, J= 8.00 Hz, 1H, ArH), 7.47-7.45 (m, 4H, ArH), 7.39 (t, J= 8.00 Hz, 1H, ArH), 7.17-7.13 (m, 2H, ArH), 7.08 (d, J= 6.30 Hz, 1H, ArH), 3.84 (t, J= 5.75 Hz, 1H, CH-COOH), 2.90 (dd, J= 5.70, 5.70 Hz, 1H, CH_a of CH_2), 2.67 (dd, J= 9.15, 9.15 Hz, 1H, CH_b of CH_2). ^{13}C NMR (DMSO-d_6, 400MHz) δ: 172.8, 167.8 (2C=O), 141.5, 137.2, 133.4, 132.6, 131.3, 129.8, 129.7, 129.4, 129.1, 128.7, 127.1, 126.7 (twelve aromatic carbons), 57.9, 38.3 (aliphatic carbons). HRMS-ESI (m/z): 410.1874 (M+H), calculated 410.1876.

2-[*N*-(benzenesulfonyl)-1-phenylformamido]-3-(1*H*-indol-2-yl)propanoic acid (5k)

Yield (0.4480 g, 99.89%), mp, 110.90 °C, FTIR (KBr, cm^{-1}): 3385 (OH of COOH), 3305 (NH of indole), 3072 (C-H of aromatic), 2839 (C-H aliphatic), 1747, 1687 (2C=O), 1602, 1584, 1454, 1425 (C=C), 1339, 1292 (2S=O), 1162, 1129 (SO_2N), 1083, 1027 (C-O, C-N). ^1H NMR (DMSO-d_6, 500MHz) δ: 10.79 (s, 1H, NH), 7.92-7.90 (m, 3H, ArH), 7.60-7.56 (m, 2H, ArH), 7.49-7.45 (m, 3H, ArH), 7.36 (t, J= 7.45 Hz, 2H, ArH), 7.28-7.25 (m, 2H, ArH), 7.00 (t, J= 6.85 Hz, 2H, ArH), 6.89 (t, J= 7.45 Hz, 1H, ArH), 3.88 (dd, J= 7.45, 8.05 Hz, 1H, CH-COOH), 3.02 (dd, J= 6.85, 6.30 Hz, 1H, CH_a of CH_2), 2.82 (dd, J= 7.45, 7.45 Hz, 1H, CH_b of CH_2). ^{13}C NMR (DMSO-d_6, 500MHz) δ: 173.1, 167.9 (2C=O), 141.4, 136.6, 133.4, 132.6, 131.3, 129.8, 129.2, 129.1, 127.5, 126.7, 124.4, 121.4, 118.9, 118.4, 111.9, 109.4 (sixteen aromatic carbons), 57.2, 28.8 (aromatic carbons). HRMS-ESI (m/z): 449.2109 (M+H), calculated, 449.2101.

2-[*N*-(benzenesulfonyl)-1-phenylformamido]-3-methylpentanoic acid (5l)

Yield (0.3750 g, 99.89%), mp, 90.40 ^oC, FTIR (KBr, cm^{-1}): 3295 (OH of COOH), 3072 (C-H aromatic), 2969, 2883 (C-H aliphatic), 1723, 1698 (C=O), 1619, 1603, 1584, 1454, 1425 (C=C), 1327, 1293 (2S=O), 1169, 1129 (SO_2N), 1092, 1074, 1027 (C-N, C-O). ^1H NMR (DMSO-d_6, 500MHz) δ: 789 (d, J= 7.15 Hz, 2H, ArH), 7.72 (d, J= 7.15 Hz, 1H, ArH), 7.58-7.54 (m, 2H, ArH), 7.51-7.43 (m, 5H, ArH), 3.50 (t, J= 6.30 Hz, 1H, CH-COOH), 1.62-1.58 (m, 1H, CH), 1.30-1.25 (m, 1H, CH_a, CH_2), 1.06-0.99 (m, 1H, CH_b, CH_2), 0.72 (d, J= 6.90 Hz, 3H, $\underline{CH}_3$-CH), 0.67 (t, J= 7.45 Hz, 3H, $\underline{CH}_3$-CH_2). ^{13}C NMR (DMSO-d_6, 500MHz) δ: 172.7, 167.9 (2C=O), 141.5, 133.4, 132.8, 131.2, 129.8, 129.4, 129.1, 126.9 (eight aromatic carbons), 60.5, 37.4, 24.8, 15.8, 11.3 (five aliphatic carbons). HRMS-ESI (m/z): 376.2141 (M+H), calculated, 376.2144.

2-[*N*-(benzenesulfonyl)-1-phenylformamido]-3-methylpentanoic acid (5m)

Yield (0.3050 g, 84.37%), mp, 104.60 ^oC, FTIR (KBr, cm^{-1}): 3302 (OH of COOH), 3073, 2970 (C-H aromatic), 2877 (C-H aliphatic), 1734, 1688 (2C=O), 1603, 1584, 1454, 1425 (C=C), 1327, 1293 (2S=O), 1169, 1129 (SO_2N), 1128, 1093, 1074 (C-O, C-N). ^1H NMR (DMSO-d_6, 500MHz) δ: 7.92-7.91 (m, 3H, ArH), 7.74 (t, J= 7.45 Hz, 1H, ArH), 7.59-7.55 (m, 2H, ArH), 7.51 (d, J= 7.45 Hz, 1H, ArH), 7.49-7.45 (m, 3H, ArH), 3.49 (d, J= 6.30 Hz, 1H, CH-COOH), 1.91-1.86 (m, 1H, CH), 0.78-0.73 (m, 6H, $2CH_3$). ^{13}C NMR (DMSO-d_6, 500MHz) δ: 172.7, 167.8 (2C=O), 141.7, 133.4, 132.8, 131.3, 129.8, 129.4, 129.1, 127.0 (eight aromatic carbons), 56.8, 30.9, 19.5, 18.4 (aliphatic carbons). HRMS-ESI (m/z): 360.0988 (M-H), calculated, 360.0983.

***N*-(1,3-benzothiazol-2-yl)-2-[*N*-(4-nitrobenzenesulfonyl)-1-phenylformamido] acetamide (17a)**

Yield (0.3888 g, 78.37%), mp, 146.20-146.80 ^oC, FTIR (KBr, cm^{-1}): 3347 (NH), 3106 (C-H aromatic), 1727, 1698 (C=O), 1640 (C=N), 1606, 1585, 1469, 1444 (C=C), 1525 (NO_2), 1353, 1304 (2S=O), 1199, 1164 (SO_2N), 1092, 1072, 1013 (C-N). ^1H NMR (DMSO-d_6, 400 MHz) δ: 8.36-8.30 (m, 3H, ArH), 8.02-7.98 (m, 3H, ArH), 7.91-7.89 (m, 1H, ArH), 7.64 (d, J= 7.32 Hz, 1H, ArH), 7.45 (t, J= 7.56 Hz, 1H, ArH), 7.31 (d, J= 7.80 Hz, 1H, ArH), 7.21 (t, J= 7.1 Hz, 2H, ArH), 7.02 (t, J= 7.10 Hz, 2H, ArH), 3.66 (s, 2H, CH_2). ^{13}C NMR (DMSOd_6, 400MHz) δ: 170.66, 167.52 (C=O), 149.86, 146.99, 146.17, 133.39, 129.94, 129.78, 129.09, 128.91, 128.64, 126.38, 124.89, 124.73, 122.06, 121.84, 117.49 (fifteen aromatic carbons), 48.82 (aliphatic carbon). HRMS (m/z): 497.0612 (M+H), calculated: 497.0618.

N-(1,3-benzothiazol-2-yl)-2-[N-(4-nitrobenzenesulfonyl)-1-phenylformamido]-3-phenyl propanamide (17b)

Yield (0.4922 g, 83.98%), mp, 111.50-111.70 ^oC, FTIR (KBr, cm^{-1}): 3414 (NH), 3087 (C-H aromatic), 2926 (C-H aliphatic), 1700, 1687 (C=O), 1639 (C=N), 1607, 1583, 1470, 1455 (C=C), 1530, 1496 (NO_2), 1349, 1310 (2S=O), 1160 (SO_2N), 1092, 1013 (C-N). ^1H NMR ($DMSOd_6$, 400MHz) δ: 8.14 (d, J= 9.16 Hz, 2H, ArH), 7.91 (d, J= 7.32 Hz, 1H, ArH), 7.70 (d, J= 8.72 Hz, 2H, ArH), 7.45 (d, J= 9.16 Hz, 2H, ArH), 7.29 (d, J= 7.76 Hz, 1H, ArH), 7.21-7.15 (m, 3H, ArH), 7.12-7.07 (m, 5H, ArH), 6.95 (t, J= 7.56 Hz, 1H, ArH), 3.96 (dd, J= 3.64, 6.40 Hz, 1H, CH-C=O), 2.96 (dd, J= 4.56, 5.04 Hz, 1H, CH_a of CH_2), 2.68 (dd, J= 10.56, 10.52 Hz, 1H, CH_b of CH_2). ^{13}C NMR ($DMSOd_6$, 400MHz) δ: 172.72, 167.00 (C=O), 153.30 (C=N), 149.58, 147.16, 137.86, 137.22, 131.43, 129.79, 129.72, 129.41, 129.07, 128.71, 128.61, 128.44, 128.16, 126.87, 125.92, 125.82, 124.62, 121.33, 118.23 (nineteen aromatic carbons), 58.23, 38.09 (two aliphatic carbons). HRMS (m/z): 586.0991 (M^+), calculated, 586.0981.

N-(1,3-benzothiazol-2-yl)-3-(1H-indol-2-yl)-2-[N-(4-nitrobenzenesulfonyl)-1-phenyl formamido]propanamide (17c)

Yield (0.6209 g, 99.33%), mp, 140.00-140.40 ^oC, FTIR (KBr, cm^{-1}): 3413, 3362 (2NH), 3004 (C-H aromatic), 2984 (C-H aliphatic), 1693, 1640 (C=O), 1619 (C=N), 1601, 1458, 1403 (C=C), 1528 (NO_2), 1349, 1312 (2S=O), 1163 (SO_2N), 1091, 1012 (C-N). ^1H NMR ($DMSOd_6$, 400MHz) δ: 10.80 (NH of indole), 8.66 (s, 1H, NH of amide), 7.93 (d, J= 7.36 Hz, 1H, ArH), 7.83 (d, J= 8.68 Hz, 2H, ArH), 7.58 (d, J= 7.80 Hz, 1H, ArH), 7.46 (t, J= 8.24 Hz, 2H, ArH), 7.19 (t, J= 7.54 Hz, 4H, ArH), 7.12-7.04 (m, 5H, ArH), 6.97-6.80 (m, 3H, ArH), 3.94 (dd, J= 4.60, 2.76 Hz, 1H, CH-C=O), 3.08 (dd, J= 4.12, 3.68 Hz, 1H, CH_a of CH_2), 2.83 (dd, J= 10.52, 10.08 Hz, 1H, CH_b of CH_2). ^{13}C NMR ($DMSOd_6$, 400MHz) δ: 173.44, 167.09 (C=O), 153.38 (C=N), 148.83, 146.49, 137.85, 137.55, 136.50, 129.40, 129.03, 128.70, 128.17, 127.34, 126.99, 125.81, 125.48, 124.96, 123.71, 121.32, 121.27, 121.12, 118.71, 118.24, 111.72, 109.21 (twenty three aromatic carbons), 57.16, 28.19 (two aliphatic carbons). HRMS (m/z): 625.1110 (M^+), calculated, 625.1109.

N-(1,3-benzothiazol-2-yl)-4-methyl-2-[N-(4-nitrobenzenesulfonyl)-1-phenylformamido] pentanamide (17d)

Yield (0.4824 g, 87.78%), mp, 197.20-198.10 °C, FTIR (KBr, cm^{-1}): 3415 (NH), 3101 (C-H aromatic), 2958 (C-H aliphatic), 1692, 1654 (C=O), 1620 (C=N), 1607, 1568, 1437 (C=C), 1531, 1497 (NO_2), 1349, 1312 (2S=O), 1182, 1166 (SO_2N), 1092, 1003 (C-N). ^1H NMR ($DMSOd_6$, 400MHz) δ: 8.34 (d, J= 8.72 Hz, 2H, ArH), 7.98 (d, J= 8.68 Hz, 2H, ArH), 7.90 (d, J= 7.80 Hz, 1H, ArH), 7.58 (t, J= 7.76 Hz, 1H, ArH), 7.44 (t, J=7.76 Hz, 1H, ArH), 7.27 (d, J= 7.76 Hz, 1H, ArH), 7.19 (t, J= 7.32 Hz, 2H, ArH), 7.15-7.07 (m, 2H, ArH), 6.94 (t, J= 7.56 Hz, 1H, ArH), 6.52 (s, 1H, NH), 3.72 (t, J= 2.76 Hz, 1H, CH-C=O), 1.41-1.34 (m, 1H, CH), 1.22-1.18 (m, 2H, CH_2), 0.82-0.76 (m, 6H, $2CH_3$). ^{13}C NMR ($DMSOd_6$, 400MHz) δ: 173.37, 167.85 (C=O), 153.31 (C=N), 149.90, 147.29, 137.86, 133.37, 131.30, 129.79, 129.41, 129.08, 128.72, 128.65, 125.91, 124.82, 121.35, 118.24 (fifteen aromatic carbons), 54.71, 31.48, 23.13, 14.48 (four aliphatic carbons). HRMS (m/z): 552.1138 (M^+), calculated, 552.1137.

N-(1,3-benzothiazol-2-yl)-3-methyl-2-[N-(4-nitrobenzenesulfonyl)-1-phenylformamido] pentanamide (17e)

Yield (0.5514 g, 99.87%), mp, 158.30-158.90 °C, FTIR (KBr, cm^{-1}): 3391 (NH), 3065 (C-H aromatic), 2957 (C-H aliphatic), 1693, 1640 (C=O), 1613 (C=N), 1602, 1583, 1469, 1455 (C=C), 1529 (NO_2), 1349, 1308 (2S=O), 1164, 1143 (SO_2N), 1092, 1067, 1020 (C-N). ^1H NMR ($DMSOd_6$, 400 MHz) δ: 8.34 (d, J= 8.68 Hz, 2H, ArH), 8.98 (d, J= 8.24 Hz, 2H, ArH), 7.91 (d, J= 7.32 Hz, 1H, ArH), 7.59 (t, J= 7.80 Hz, 1H, ArH), 7.45 (t, J= 7.76 Hz, 1H, ArH), 7.28 (d, J= 8.28 Hz, 1H, ArH), 7.20 (d, J= 7.34 Hz, 2H, ArH), 7.13-7.08 (m, 2H, ArH), 6.95 (t, J= 7.32 Hz,1H, ArH), 6.44 (s, 1H, NH), 3.73 (d, J= 5.15 Hz, 1H, CH-C=O), 1.42-1.34 (m, 1H, CH), 1.22-1.18 (m, 2H, CH_2), 0.80-0.70 (m, 6H, $2CH_3$). ^{13}C NMR ($DMSOd_6$, 400MHz) δ: 173.38, 167.86 (C=O), 166.98 (C=N), 153.33 (C-NO_2), 149.89, 147.30, 137.86, 133.35, 131.45, 129.79, 129.41, 129.07, 128.72, 125.91, 125.83, 124.82, 121.36, 121.29, 118.22 (seventeen aromatic carbons), 54.74, 31.49, 24.49, 23.13, 14.47 (five aliphatic carbons). HRMS (m/z): 553.1242 (M+H), calculated, 553.1249.

***N*-(1,3-benzothiazol-2-yl)-3-methyl-2-[N-(4-nitrobenzenesulfonyl)-1-phenylformamido] butanamide (17f)**

Yield (0.5309 g, 98.66%), mp, 162.90-163.30 °C, FTIR (KBr, cm^{-1}): 3398 (NH), 3110 (C-H aromatic), 2987, 2899 (C-H aliphatic), 1696, 1659 (C=O), 1618 (C=N), 1601, 1592, 1487 (C=C), 1531, 1515 (NO_2), 1356, 1334 (2S=O), 1168, 1139 (SO_2N), 1094, 1071 (C-N). ^1H NMR ($DMSOd_6$, 400MHz) δ: 8.34 (d, J= 7.80 Hz, 2H, ArH), 8.00 (d, J= 8.72 Hz, 1H, ArH), 7.61 (d, J=

7.80 Hz, 1H, ArH), 7.29 (t, J= 8.70 Hz, 2H, ArH), 7.20 (t, J= 7.56 Hz, 2H, ArH), 7.13-7.08 (m, 4H, ArH), 7.98 (t, J= 7.34 Hz, 1H, ArH), 6.54 (s, 1H, NH), 3.60 (d, J= 5.96 Hz, 1H, CH-C=O), 1.99-1.94 (m, 1H, CH), 0.81-0.76 (m, 6H, $2CH_3$). ^{13}C NMR ($DMSOd_6$, 400MHz) δ: 172.42, 167.22 (C=O), 160.11 (C=N), 150.26 ($C-NO_2$), 137.87, 133.87, 131.09, 130.83, 129.42, 129.09, 128.72, 128.45, 126.11, 125.83, 124.76, 121.56, 117.92 (fifteen aromatic carbons), 61.97, 31.51, 19.56, 18.24 (four aliphatic carbons). HRMS (m/z): 538.0980 (M^+), calculated, 538.0981.

N-(1,3-benzothiazol-2-yl)-4-hydroxy-1-(4-nitrobenzenesulfonyl)pyrrolidine-2-carboxamide (17g)

Yield (0.4479 g, 99.96%), mp, 127.40-127.90 °C, FTIR (KBr, cm^{-1}): 3492 (NH), 3389 (OH), 3118 (C-H aromatic), 2987, 2899 (C-H aliphatic), 1750 (C=O), 1646 (C=N), 1608, 1451, 1403 (C=C), 1528 (NO_2), 1354, 1331 (2S=O), 1184, 1164 (SO_2N), 1090, 1069, 1016, 1005 (C-N, C-O). ^1H NMR ($DMSOd_6$, 400MHz) δ: 8.35 (d, J= 8.72 Hz, 2H, ArH), 8.02 (d, J= 8.72 Hz, 2H, ArH), 7.59 (d, J= 7.80 Hz, 1H, ArH), 7.27 (d, J= 7.76 Hz, 1H, ArH), 7.20-7.14 (m, 2H, ArH), 6.50 (s, 1H, NH), 4.16 (s, 1H, OH), 4.09 (t, J= 5.32 Hz, 1H, CH-C=O), 3.46-3.42 (m, 1H, CH-OH), 3.19 (d, J= 5.95 Hz, 2H, CH_2N), 2.03-1.98 (m, 1H, CH of CH_2), 1.92-1.86 (m, 1H, CH of CH_2). ^{13}C NMR ($DMSOd_6$, 400MHz) δ: 175.76 (C=O), 173.53, 166.95, 163.69, 150.32, 143.47, 129.53, 125.95, 124.85, 121.39, 121.34, 118.20 (eleven aromatic carbons), 68.99, 60.38, 57.14, 30.77 (four aliphatic carbons). HRMS (m/z): 449.0586 (M+H), calculated, 449.0588.

N-(1,3-benzothiazol-2-yl)-1-(4-nitrobenzenesulfonyl)pyrrolidine-2-carboxamide (17h)

Yield (0.4320 g, 99.98%), mp, 155.10 °C, FTIR (KBr, cm^{-1}): 3419 (NH), 3106 (C-H aromatic), 2981 (C-H aliphatic), 1703 (C=O), 1623 (C=N), 1604, 1454, 1401 (C=C), 1531, 1495 (NO_2), 1350, 1313 (2S=O), 1200, 1164 (SO_2N), 1093, 1065, 1011 (C-N). ^1H NMR ($DMSOd_6$, 400MHz) δ: 8.4193-8.3574 (m, 2H, ArH), 8.1146-8.0447 (m, 2H, ArH), 7.9725-7.9531 (d, J= 7.76 Hz, 1H, ArH), 7.7469-7.7274 (d, J= 7.80 Hz, 1H, ArH), 7.2211-7.0779 (m, 2H, ArH), 6.5029 (s, 1H, NH), 4.4399-4.4089 (dd, J= 4.16, 4.12 Hz, 1H, CH-C=O), 4.1890-4.1581 (m, 1H, CH_a of CH_2N), 3.5555-3.5006 (m, 1H, CH_b of CH_2N), 3.2279-3.1672 (m, 1H, CH_a of CH_2), 2.0103-1.7457 (m, 2H, CH_2), 1.6368-1.5899 (m, 1H, CH_b of CH_2). ^{13}C NMR ($DMSOd_6$, 400MHz) δ: 173.4140 (C=O), 166.9951 (C=N), 150.4116 ($C-NO_2$), 143.7725, 129.3732, 128.7314, 125.8381, 125.1387, 122.3317, 121.3833, 121.1725, 118.1739 (eleven aromatic carbons), 61.0656,

48.9465, 31.5774, 24.9670 (four aliphatic carbons). HRMS (m/z): 433.0649 (M+H), calculated, 433.0652.

N-(1,3-benzothiazol-2-yl)-4-hydroxy-1-(4-methylbenzenesulfonyl)pyrrolidine-2-carboxamide (17i)

Yield (0.4171 g, 100%), mp, 177.00 °C, FTIR (KBr, cm^{-1}): 3412 (NH), 3376 (OH), 3004 (C-H aromatic), 2949 (C-H aliphatic), 1725 (C=O), 1647 (C=N), 1598, 1495, 1470, 1404 (C=C), 1333, 1312 (2S=O), 1198, 1155 (SO_2N), 1088, 1011 (C-N). ^{1}H NMR ($DMSOd_6$, 400MHz) δ: 7.6530-7.6323 (d, J= 8.28 Hz, 2H, ArH), 7.6117-7.5945 (d, J= 6.88 Hz, 1H, ArH), 7.4788 (s, 1H, NH), 7.3666-7.3460 (d, J= 8.24 Hz, 2H, ArH), 7.3001-7.2807 (d, J= 7.76 Hz, 1H, ArH), 7.2016-7.1146 (m, 1H, ArH), 6.9863-6.9485 (t, J= 7.56 Hz, 1H, ArH), 4.1718 (s, 1H, OH), 4.0172-3.9782 (t, J= 7.80 Hz, 1H, CH-C=O), 3.4318-3.3940 (m, 1H, <u>CH</u>-OH), 3.0618-3.0309 (d, J= 6.82 Hz, 2H, CH_2N), 2.3413 (s, 3H, CH_3-Ar), 1.9175-1.8866 (m, 2H, CH_2). ^{13}C NMR ($DMSOd_6$, 400MHz) δ: 173.8163 (C=O), 167.0622 (C=N), 152.8354, 143.7629, 134.9298, 131.1935, 130.1013, 129.4307, 128.7314, 127.9745, 126.0297, 121.4599, 118.1355 (eleven aromatic carbons), 68.9119, 60.1650, 56.7736, 21.5276 (four aliphatic carbons). HRMS (m/z): 416.0736 (M-H), calculated, 416.0739.

N-(1,3-benzothiazol-2-yl)-1-(4-methylbenzenesulfonyl)pyrrolidine-2-carboxamide (17j)

Yield (0.4010 g, 99.98%), pale yellowish oil, FTIR (KBr, cm^{-1}): 3356 (NH), 3021 (C-H aromatic), 2994 (C-H aliphatic), 1701 (C=O), 1623 (C=N), 1608, 1495, 1470 (C=C), 1339, 1322 (2S=O), 1198, 1155 (SO_2N), 1088, 1071 (C-N). ^{1}H NMR ($CDOD_3$, 400MHz) δ: 7.6530-7.6323 (d, J= 8.28 Hz, 2H, ArH), 7.6117-7.5945 (d, J= 6.88 Hz, 1H, ArH), 7.3011-7.2817 (d, J= 7.76 Hz, 1H, ArH), 7.2211-7.0779 (m, 2H, ArH), 6.5007 (s, 1H, NH), 4.4399-4.4089 (dd, J= 4.16, 4.12 Hz, 1H, CH-C=O), 4.1890-4.1581 (m, 1H, CH_a of CH_2N), 3.5555-3.5006 (m, 1H, CH_b of CH_2N), 3.2279-3.1672 (m, 1H, CH_a of CH_2), 2.0103-1.7457 (m, 2H, CH_2), 1.6368-1.5899 (m, 1H, CH_b of CH_2). ^{13}C NMR ($CDOD_3$, 400MHz) δ: 174.7552 (C=O), 168.7388 (C=N), 152.1176 ($C-NO_2$), 144.0216, 137.5740, 134.7574, 129.7565, 128.6164, 125.6369, 124.9855, 123.8646, 116.8326 (eleven aromatic carbons), 60.7303, 30.7151, 24.2963, 22.2803, 20.1864 (five aliphatic carbons). HRMS (m/z): 400.0796 (M-H), calculated, 400.0801.

1-(Benzenesulfonyl)-N-(1,3-benzothiazol-2-yl)-4-hydroxypyrrolidine-2-carboxamide (17k)

Yield (0.4031 g, 100%), pale yellowish oil, FTIR (KBr, cm^{-1}): 3432 (NH), 3316 (OH), 3098 (C-H aromatic), 2988, 2879 (C-H aliphatic), 1692 (C=O), 1619 (C=N), 1605, 1592, 1454 (C=C), 1361, 1319 (2S=O), 1179, 1136 (SO_2N), 1095, 1073, 1027 (C-N, C-O). ^1H NMR ($CDOD_3$, 400MHz) δ: 7.8587-7.8404 (d, J= 7.32 Hz, 2H, ArH), 7.6312-7.5945 (t, J= 7.34 Hz, 1H, ArH), 7.5579-7.5197 (t, J= 7.64 Hz, 3H, ArH), 7.3685-7.3487 (d, J= 7.92 Hz, 1H, ArH), 7.2540-7.2143 (t, J= 7.94 Hz, 1H, ArH), 7.1791-7.0325 (m, 1H, ArH), 6.5405 (s, 1H, NH), 4.3021 (s, 1H, OH), 4.2410-4.2013 (t, J= 7.94 Hz, 1H, CH-C=O), 3.5783-3.5416 (m, 1H, CH-OH), 3.2805-3.2515 (d, J= 11.60 Hz, 2H, CH_2N), 2.0665-2.0008 (m, 2H, CH_2). ^{13}C NMR ($CDOD_3$, 400MHz) δ: 174.9756 (C=O), 150.5553 (C=N), 137.5548, 132.7743, 129.9864, 128.7888, 128.6068, 127.8979, 127.5338, 125.7232, 124.9855, 121.7569, 120.6935, 117.1775 (thirteen aromatic carbons), 69.1993, 60.0501, 56.2850, 39.0117 (four aliphatic carbons). HRMS (m/z): 422.0832 ($M+H_3O$), calculated, 422.0837.

1-(Benzenesulfonyl)-N-(1,3-benzothiazol-2-yl)pyrrolidine-2-carboxamide (17l)

Yield (0.3864 g, 99.82%), pale yellowish oil, FTIR (KBr, cm^{-1}): 3418 (NH), 3020 (C-H aromatic), 2987, 2895 (C-H aliphatic), 1693 (C=O), 1638 (C=N), 1601, 1447 (C=C), 1385, 1339 (2S=O), 1161 (SO_2N), 1094, 1049, 1025 (C-N). ^1H NMR ($DMSOd_6$, 400MHz) δ: 7.7755-7.7572 (d, J= 7.32 Hz, 2H, ArH), 7.6747 (s, 1H, NH), 7.6381-7.6026 (t, J= 7.10 Hz, 1H, ArH), 7.5716-7.5522 (d, J= 7.76 Hz, 2H, ArH), 7.2990-7.2784 (d, J= 8.24 Hz, 1H, ArH), 7.1787-7.1455 (t, J= 6.64 Hz, 1H, ArH), 7.0962-7.0424 (m, 1H, ArH), 6.9886-6.9508 (t, J= 7.56 Hz, 1H, ArH), 4.0630-4.0321 (t, J= 6.18 Hz, 1H, CH-C=O), 3.3505-3.3104 (m, 1H, CH_a of CH_2N), 3.1855-3.0973 (m, 1H, CH_b of CH_2N), 1.8911-1.7411 (m, 3H), 1.5406-1.4982 (m, 1H, CH of CH_2). ^1H NMR ($DMSOd_6$, 400MHz) δ: 173.6165 (C=O), 167.3809 (C=N), 151.2674, 138.0430, 133.4187, 129.7574, 129.2998, 128.5847, 127.5168, 126.0771, 125.6957, 121.7293, 121.4528, 117.6866 (thirteen aromatic carbons), 60.8415, 48.8660, 30.9410, 24.7245 (four aliphatic carbons). HRMS (m/z): 388.0784 (M+H), calculated, 388.0781.

17a

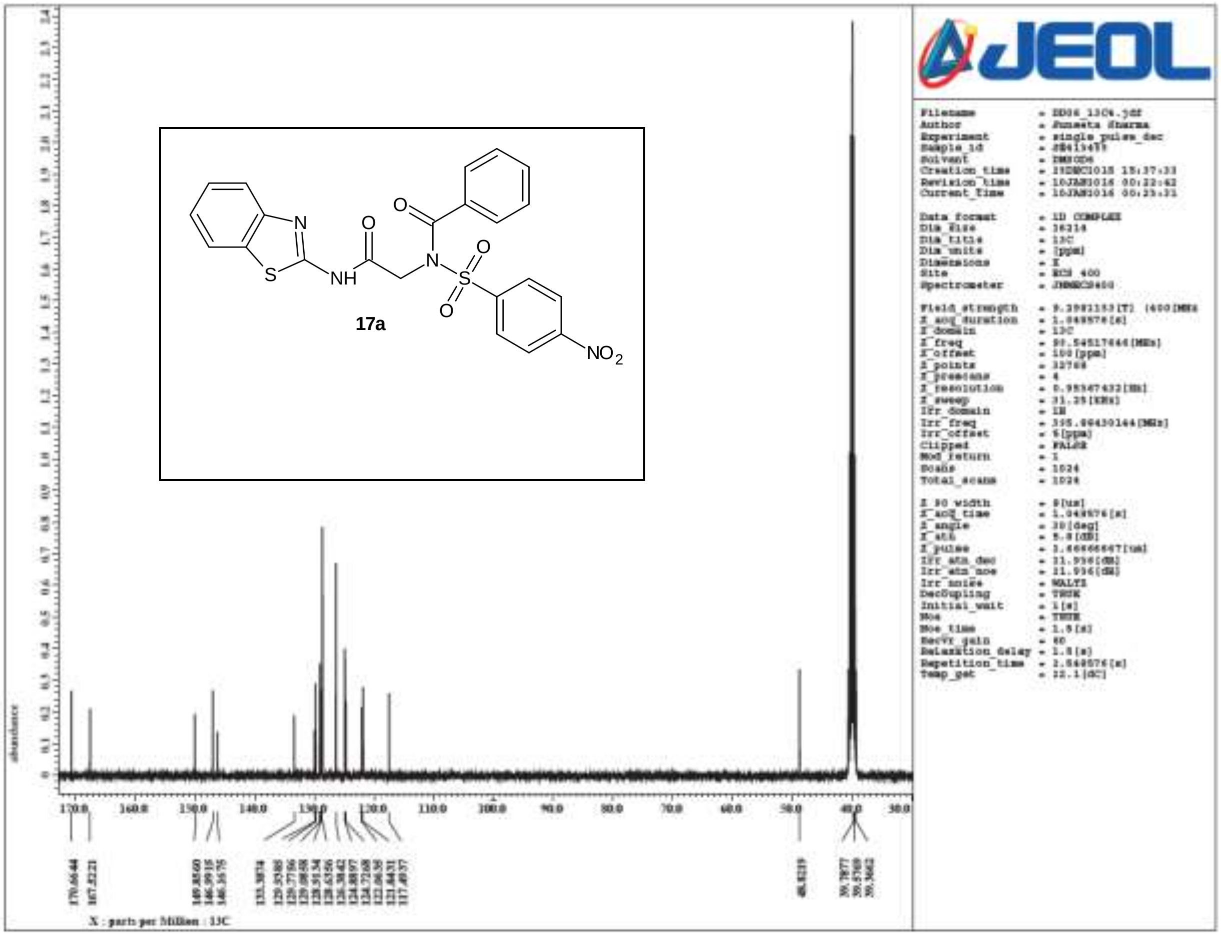

17a
N
S
NH
O
N
O
S
O
O
NO_2
JEOL
Filename
Author
Experiment = single_pulse_dec
Sample_id
Solvent
Creation_time
Revision_time
Current_Time
Data_format = 1D COMPLEX
Dim_size
Dim_title = 13C
Dim_units = [ppm]
Dimensions = X
Site
Spectrometer
Field_strength
X_acq_duration
X_domain = 13C
X_freq
X_offset = 100[ppm]
X_points = 32768
X_prescans = 4
X_resolution
X_sweep
Irr_domain = 1H
Irr_freq
Irr_offset
Clipped = FALSE
Mod_return = 1
Scans = 1024
Total_scans = 1024
X_90_width
X_acq_time
X_angle
X_atn
X_pulse
Irr_atn_dec
Irr_atn_noe
Irr_noise = WALTZ
Decoupling = TRUE
Initial_wait = 1[s]
Noe = TRUE
Noe_time
Recvr_gain
Relaxation_delay
Repetition_time
Temp_get
abundance
170.0
160.0
150.0
140.0
130.0
120.0
110.0
100.0
90.0
80.0
70.0
60.0
50.0
40.0
30.0
170.6644
167.8221
149.8560
146.9915
146.3675
133.3874
129.9380
129.7756
129.0888
128.9154
128.6356
126.3842
124.8897
124.7268
122.0630
121.6431
117.4937
48.8219
39.7877
39.5769
39.3662
X : parts per Million : 13C

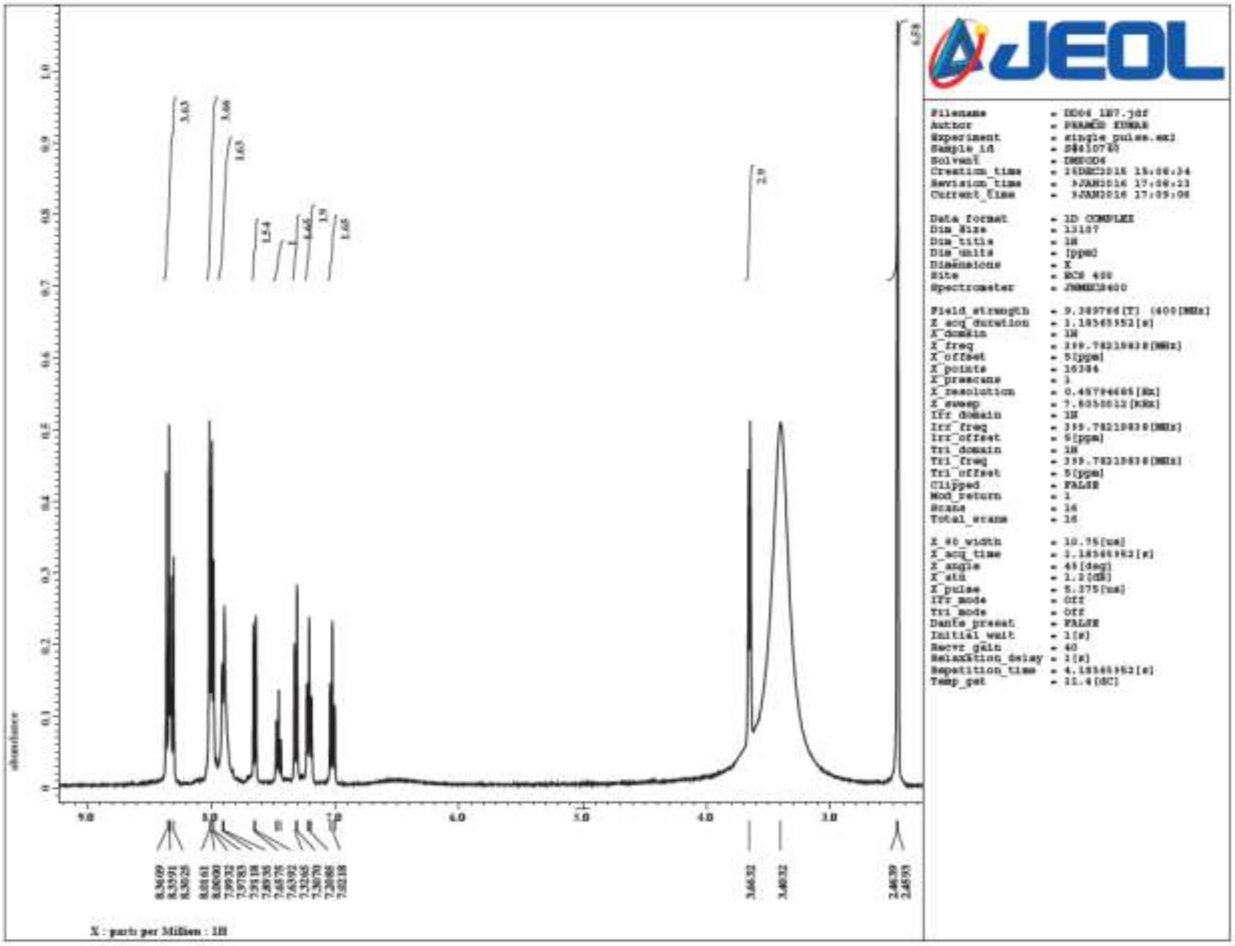

JEOL
Filename = DD04 1H7.jdf
Experiment = single_pulse.ex2
Solvent = DMSOD6
Data_format = 1D COMPLEX
Dim_title = 1H
Dim_units = [ppm]
Dimensions = X
Field_strength = 9.389766[T] (400[MHz])
X_domain = 1H
X_offset = 5[ppm]
X_points = 16384
X_prescans = 1
Irr_domain = 1H
Irr_offset = 5[ppm]
Tri_domain = 1H
Tri_offset = 5[ppm]
Clipped = FALSE
Mod_return = 1
Scans = 16
Total_scans = 16
X_90_width = 10.75[us]
X_angle = 45[deg]
X_atn = 1.2[dB]
X_pulse = 5.375[us]
Irr_mode = Off
Tri_mode = Off
Dante_presat = FALSE
Initial_wait = 1[s]
Recvr_gain = 40
Relaxation_delay = 1[s]
abundance
X : parts per Million : 1H

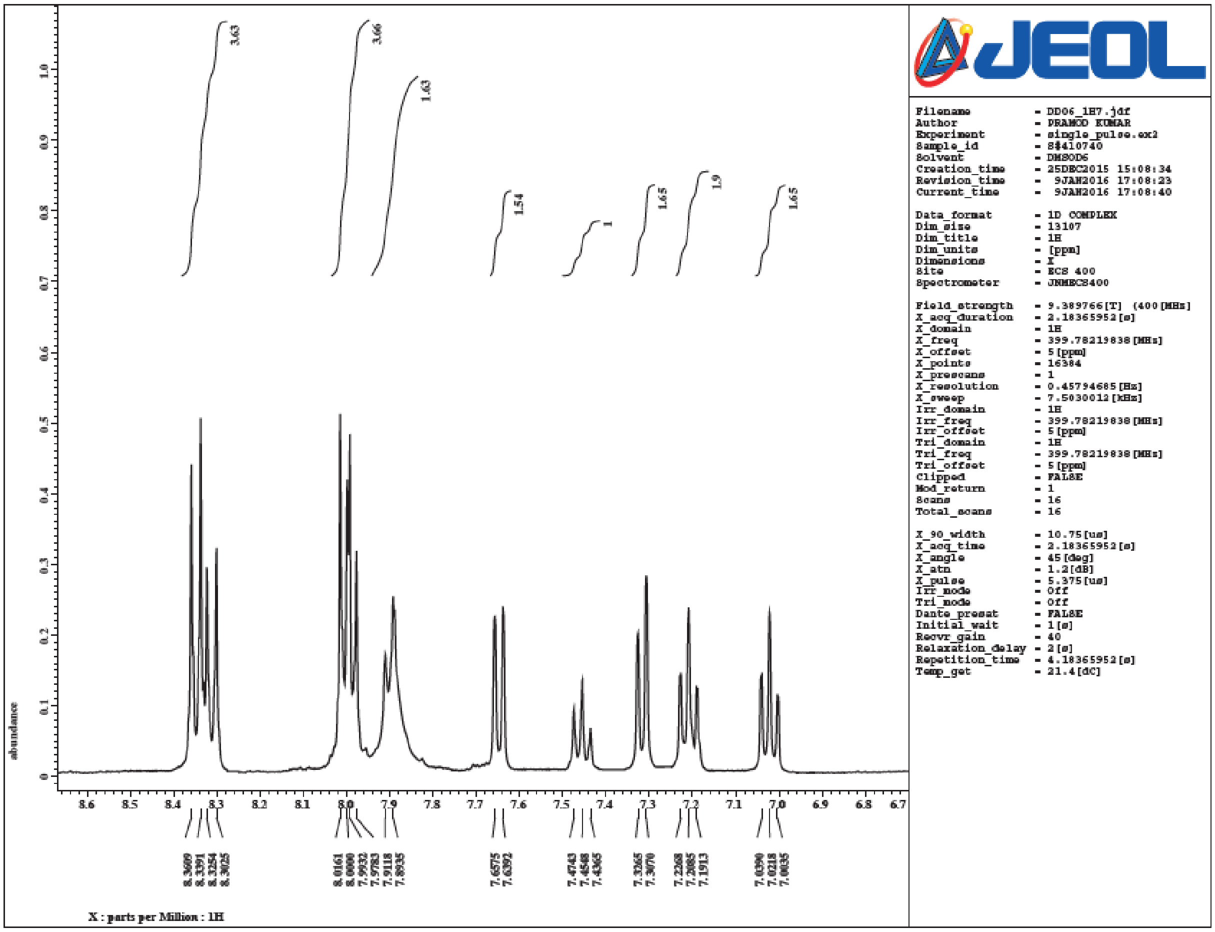

3.63
3.66
1.63
1.54
1
1.65
1.9
1.65
abundance
0
0.1
0.2
0.3
0.4
0.5
0.6
0.7
0.8
0.9
1.0
8.6
8.5
8.4
8.3
8.2
8.1
8.0
7.9
7.8
7.7
7.6
7.5
7.4
7.3
7.2
7.1
7.0
6.9
6.8
6.7
8.3609
8.3391
8.3254
8.3025
8.0161
8.0000
7.9932
7.9783
7.9118
7.8935
7.6575
7.6392
7.4743
7.4548
7.4365
7.3265
7.3070
7.2268
7.2085
7.1913
7.0390
7.0218
7.0035
X : parts per Million : 1H
JEOL
Filename - DD06_1H7.jdf
Author - PRAMOD KUMAR
Experiment - single_pulse.ex2
Sample_id - 8$410740
Solvent - DMSOD6
Creation_time - 25DEC2015 15:08:34
Revision_time - 9JAN2016 17:08:23
Current_time - 9JAN2016 17:08:40
Data_format - 1D COMPLEX
Dim_size - 13107
Dim_title - 1H
Dim_units - [ppm]
Dimensions - X
Site - ECS 400
Spectrometer - JNMECS400
Field_strength - 9.389766[T] (400[MHz]
X_acq_duration - 2.18365952[s]
X_domain - 1H
X_freq - 399.78219838[MHz]
X_offset - 5[ppm]
X_points - 16384
X_prescans - 1
X_resolution - 0.45794685[Hz]
X_sweep - 7.5030012[kHz]
Irr_domain - 1H
Irr_freq - 399.78219838[MHz]
Irr_offset - 5[ppm]
Tri_domain - 1H
Tri_freq - 399.78219838[MHz]
Tri_offset - 5[ppm]
Clipped - FALSE
Mod_return - 1
Scans - 16
Total_scans - 16
X_90_width - 10.75[us]
X_acq_time - 2.18365952[s]
X_angle - 45[deg]
X_atn - 1.2[dB]
X_pulse - 5.375[us]
Irr_mode - Off
Tri_mode - Off
Dante_presat - FALSE
Initial_wait - 1[s]
Recvr_gain - 40
Relaxation_delay - 2[s]
Repetition_time - 4.18365952[s]
Temp_get - 21.4[dC]

17b

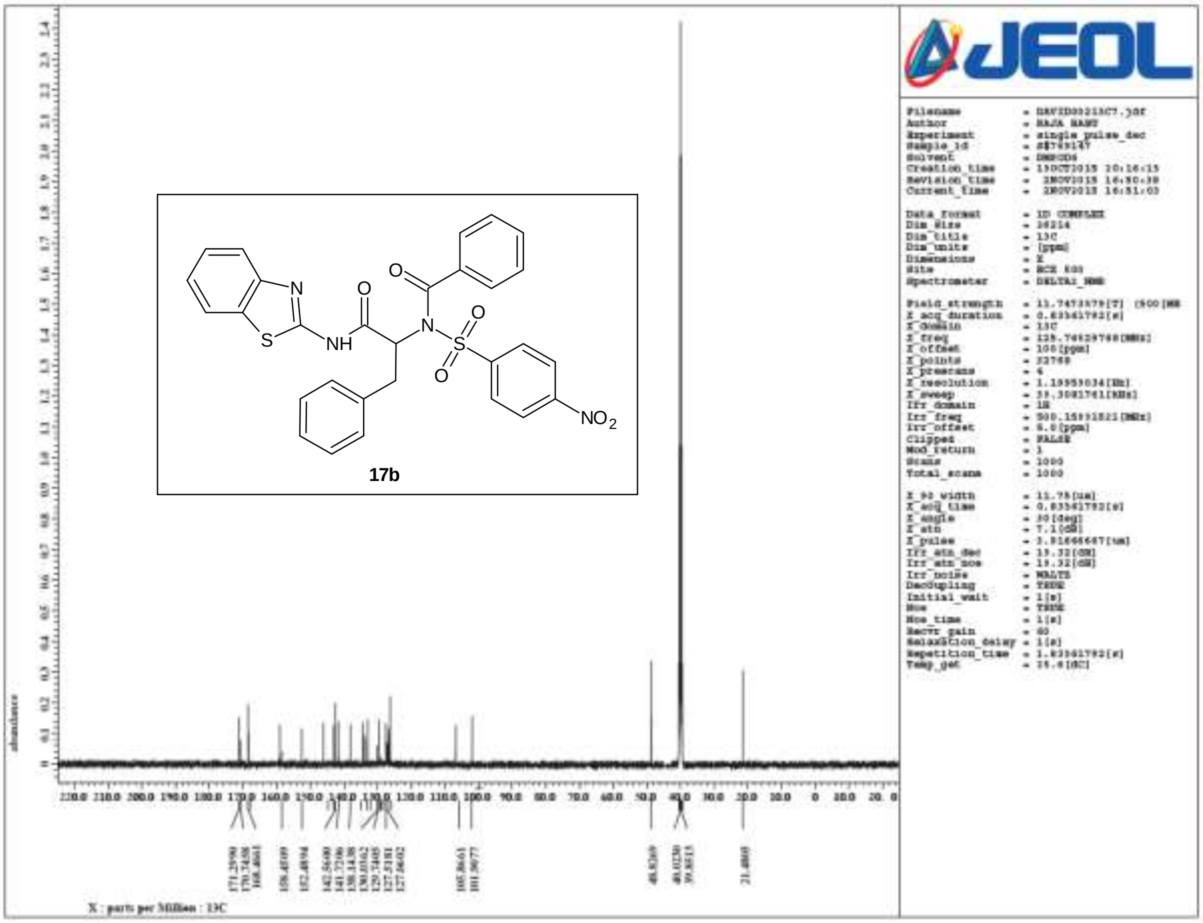
17b
abundance
X : parts per Million : 13C

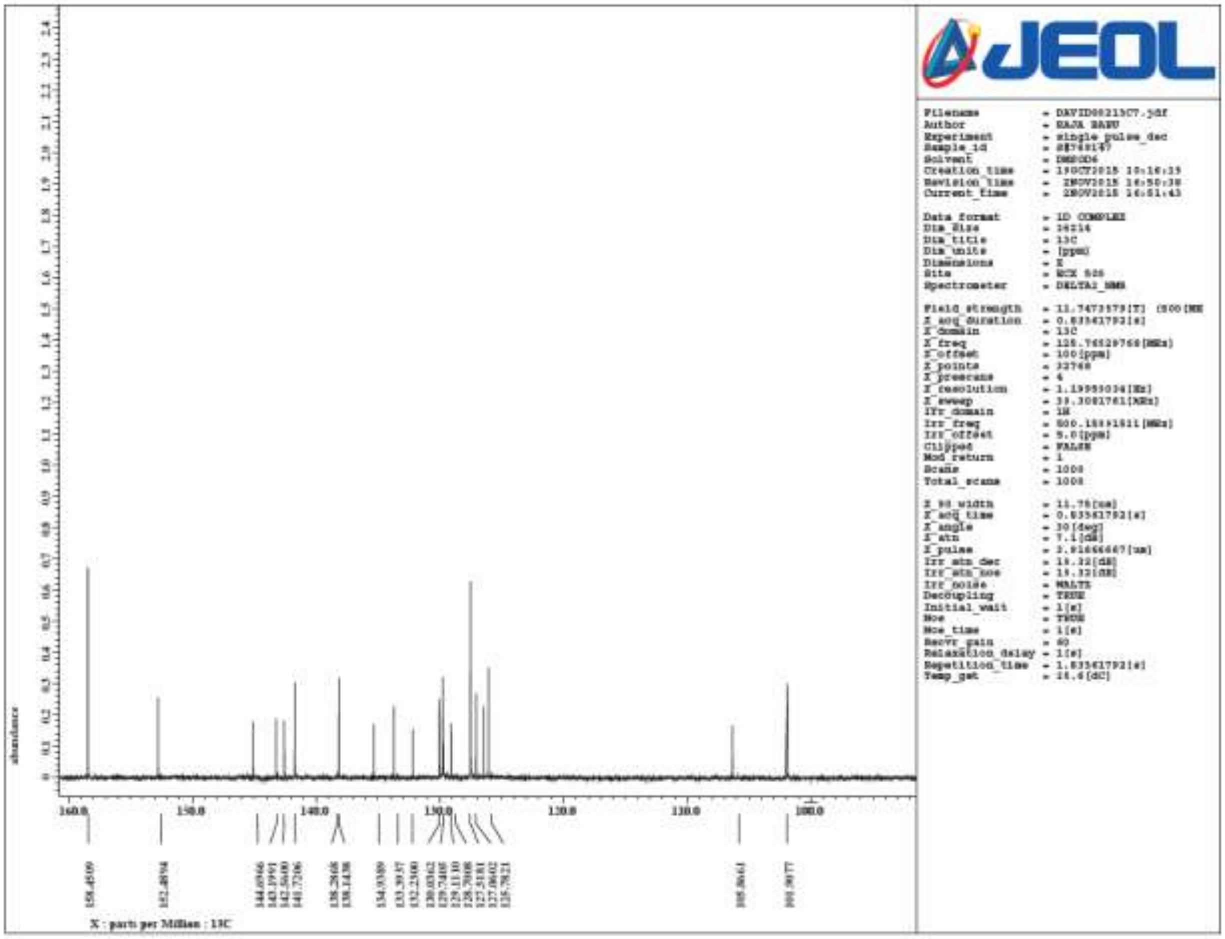
abundance
160.0
150.0
140.0
130.0
120.0
110.0
100.0
158.4509
152.4894
144.6946
143.1991
142.5600
141.7206
138.2868
138.1438
134.9389
133.3937
132.2300
130.0362
129.7416
129.1110
128.7008
127.5181
127.0602
126.7821
105.8641
101.9077
X : parts per Million : 13C
Filename = DAVID00213C7.jdf
Author = RAJA BABU
Experiment = single_pulse_dec
Solvent = DMSOD6
Data_format = 1D COMPLEX
Dim_title = 13C
Dim_units = [ppm]
Dimensions = X
Spectrometer = DELTA2_NMR
X_domain = 13C
X_offset = 100[ppm]
X_prescans = 4
Irr_domain = 1H
Irr_offset = 5.0[ppm]
Clipped = FALSE
Mod_return = 1
Scans = 1000
Total_scans = 1000
X_angle = 30[deg]
Irr_noise = WALTZ
Decoupling = TRUE
Initial_wait = 1[s]
Noe = TRUE
Noe_time = 1[s]
Relaxation_delay = 1[s]

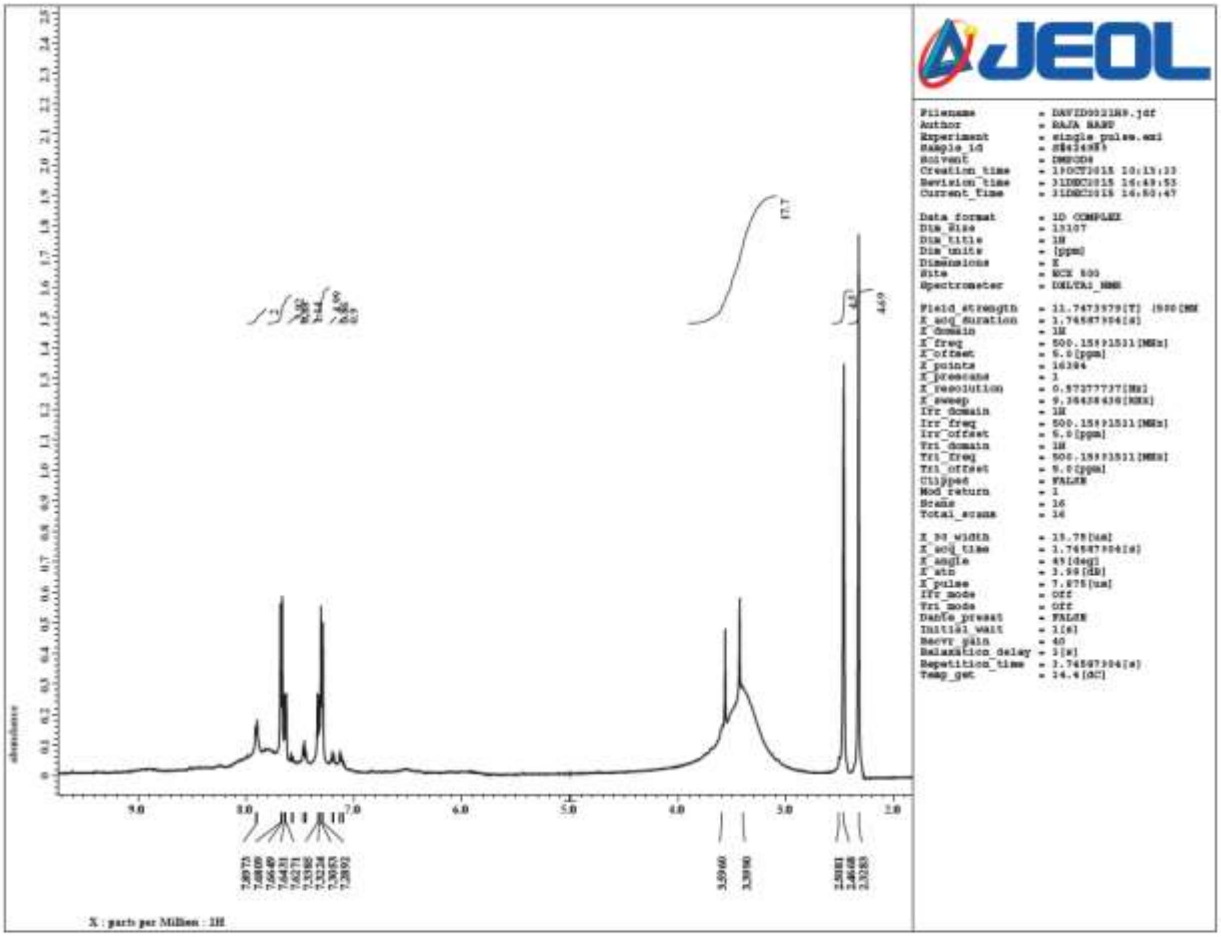
JEOL
Filename = DAVID0021H9.jdf
Author = RAJA BABU
Experiment = single_pulse.ex2
Solvent = DMSOD6
Data_format = 1D COMPLEX
Dim_title = 1H
Dim_units = [ppm]
Dimensions = X
Site = ECX 500
Spectrometer = DELTA2_NMR
X_domain = 1H
X_freq = 500.15991511[MHz]
X_offset = 5.0[ppm]
X_points = 16384
X_prescans = 1
Irr_domain = 1H
Irr_freq = 500.15991511[MHz]
Irr_offset = 5.0[ppm]
Tri_domain = 1H
Tri_freq = 500.15991511[MHz]
Tri_offset = 5.0[ppm]
Clipped = FALSE
Mod_return = 1
Scans = 16
Total_scans = 16
X_angle = 45[deg]
X_pulse = 7.875[us]
Irr_mode = Off
Tri_mode = Off
Dante_presat = FALSE
Initial_wait = 1[s]
Recvr_gain = 40
Relaxation_delay = 1[s]
Temp_get = 14.4[dC]
abundance
17.7
4.69
7.8973
7.6809
7.6649
7.6431
7.6271
7.3398
7.3224
7.3053
7.2892
3.5960
3.3990
2.5081
2.4668
2.3283
X : parts per Million : 1H

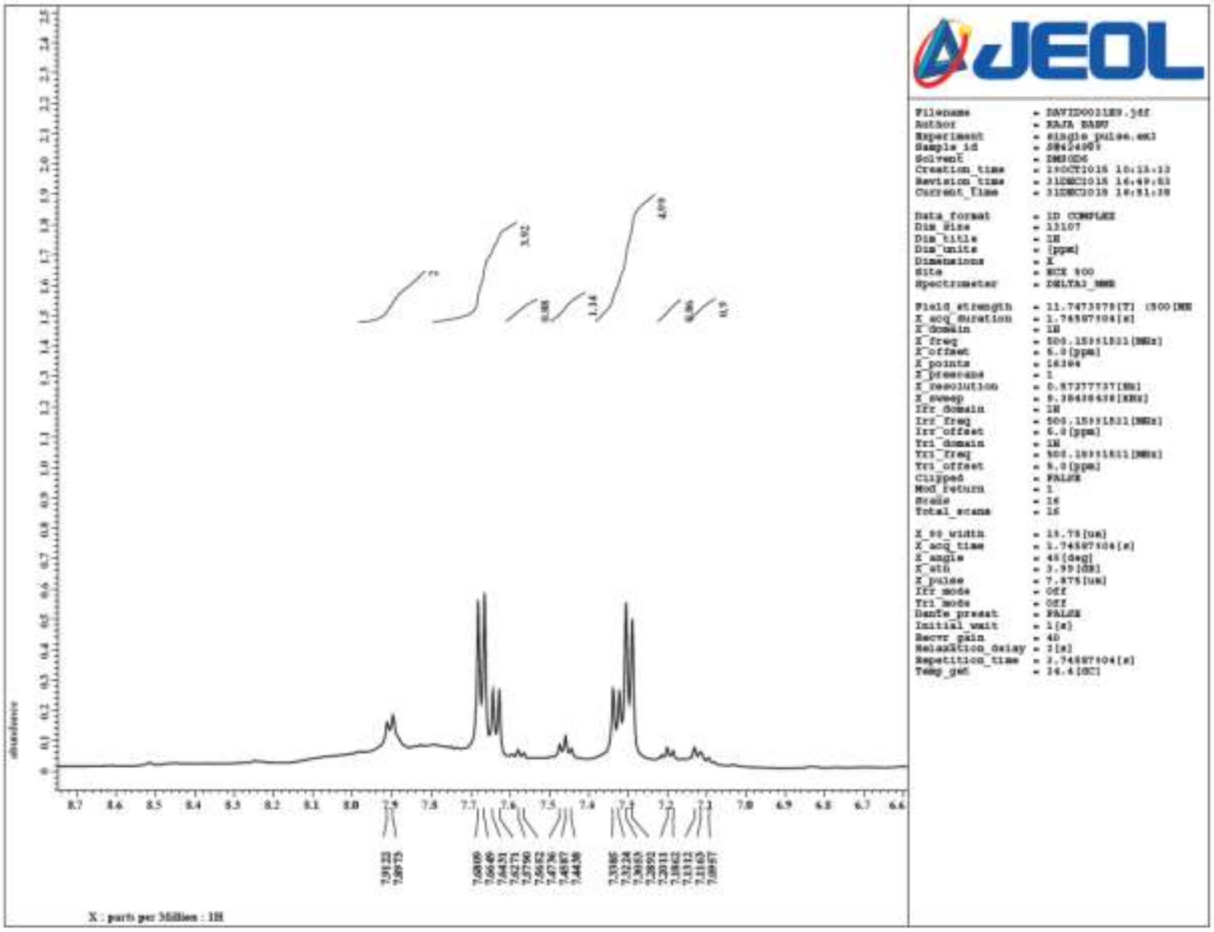

17c

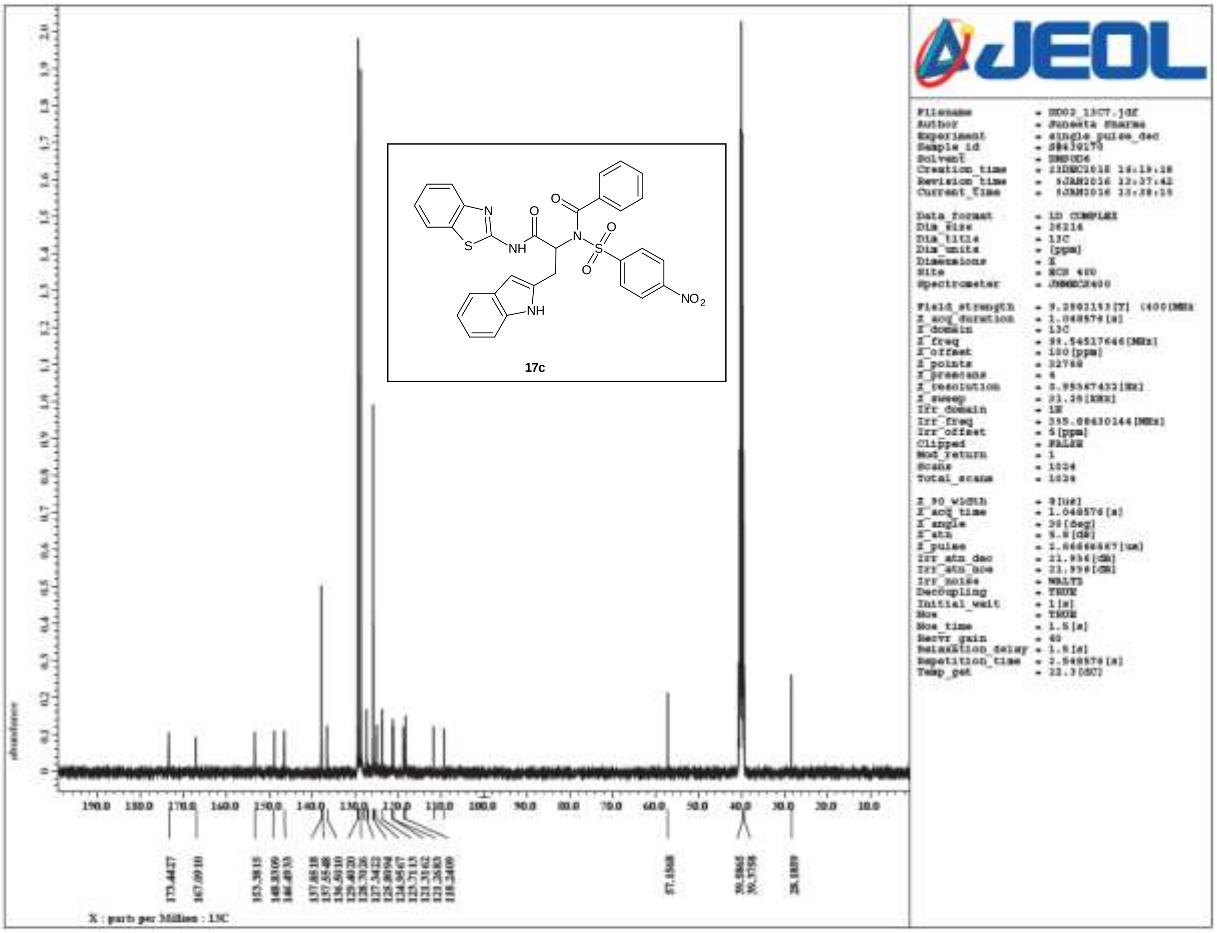
JEOL
17c
NH
S
N
O
NO2
abundance
X : parts per Million : 13C
Filename
Author
Experiment
Sample_id
Solvent
Data_format = 1D COMPLEX
Dim_title = 13C
Dim_units = [ppm]
Field_strength
X_domain = 13C
X_offset = 100[ppm]
Irr_domain = 1H
Irr_offset = 5[ppm]
Scans = 1024
Total_scans = 1024
Decoupling = TRUE
Initial_wait = 1[s]
Noe = TRUE
Noe_time = 1.5[s]
Relaxation_delay = 1.5[s]

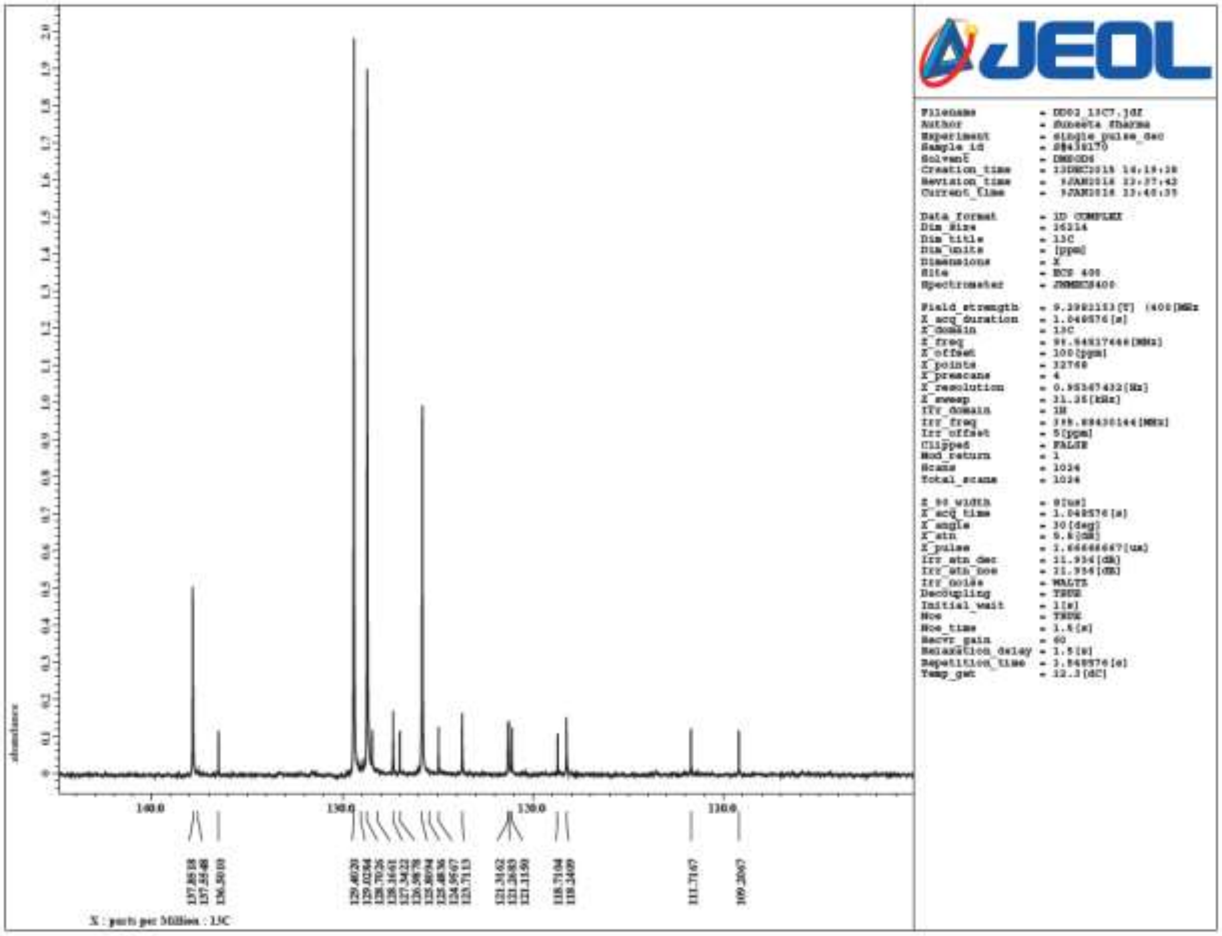

JEOL
Filename = DD02_13C7.jdf
Experiment = single_pulse_dec
Data_format = 1D COMPLEX
Dim_size = 16384
Dim_title = 13C
Dim_units = [ppm]
Dimensions = X
Site = ECS 400
Field_strength = 9.3982153[T] (400[MHz
X_acq_duration = 1.048576[s]
X_domain = 13C
X_offset = 100[ppm]
X_points = 32768
X_prescans = 4
X_sweep = 31.25[kHz]
Irr_domain = 1H
Irr_offset = 5[ppm]
Clipped = FALSE
Mod_return = 1
Scans = 1024
Total_scans = 1024
X_acq_time = 1.048576[s]
X_angle = 30[deg]
Irr_noise = WALTZ
Decoupling = TRUE
Initial_wait = 1[s]
Noe = TRUE
Noe_time = 1.5[s]
Recvr_gain = 60
Relaxation_delay = 1.5[s]
Temp_get = 12.3[dC]
abundance
140.0
130.0
120.0
110.0
137.8518
137.5548
136.5010
129.4020
129.0284
128.7026
128.1641
127.3422
126.9878
125.8094
125.4836
124.9567
123.7113
121.3162
121.2683
121.1150
118.7104
118.2409
111.7167
109.2067
X : parts per Million : 13C

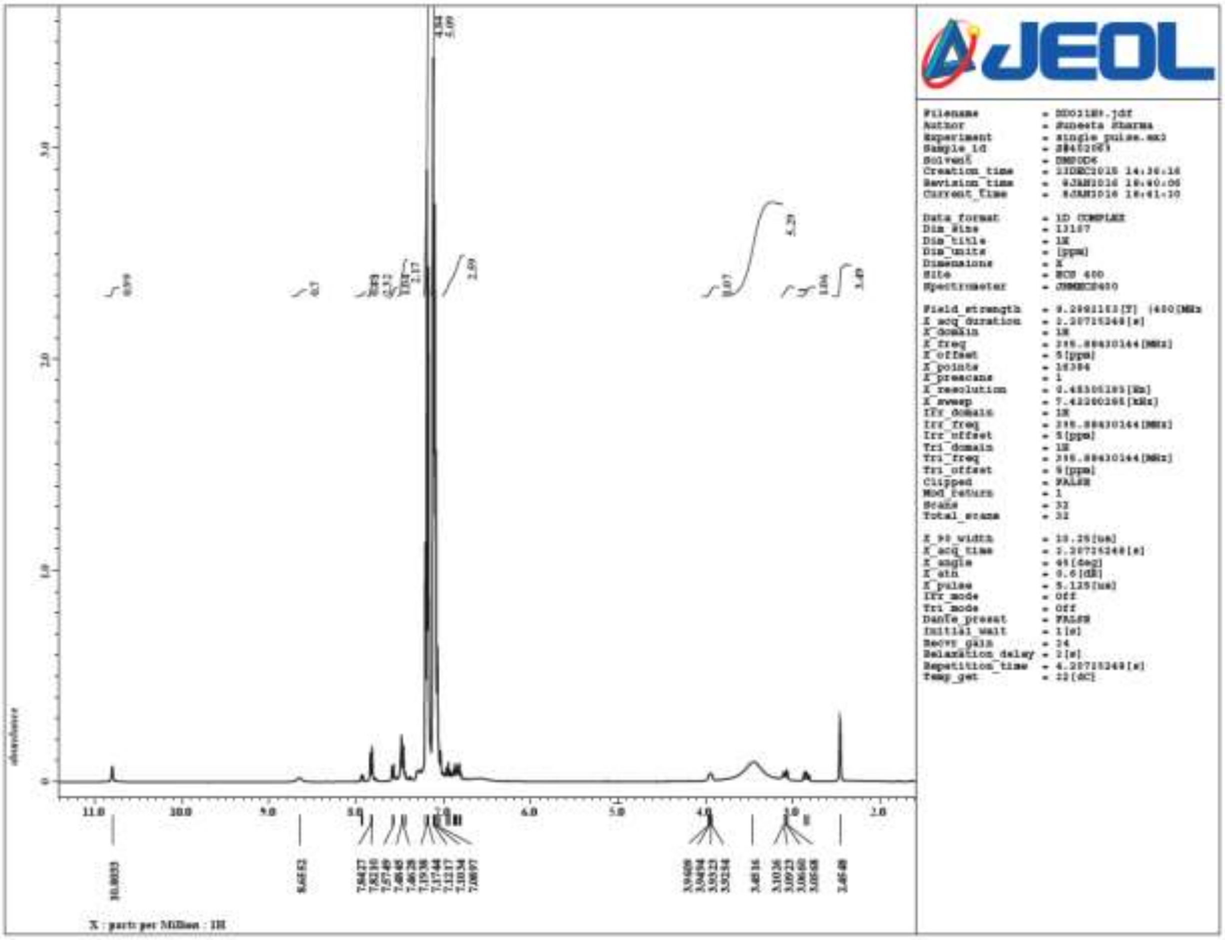
X : parts per Million : 1H
abundance
Filename
Author = Suneeta Sharma
Experiment = single_pulse.ex2
Solvent = DMSO-D6
Creation_time
Revision_time
Current_time
Data_format = 1D COMPLEX
Dim_title = 1H
Dim_units = [ppm]
Dimensions = X
Site = ECS 400
Spectrometer
Field_strength
X_acq_duration
X_domain = 1H
X_freq
X_offset = 5[ppm]
X_points = 16384
X_prescans = 1
X_resolution
X_sweep
Irr_domain = 1H
Irr_freq
Irr_offset = 5[ppm]
Tri_domain = 1H
Tri_freq
Tri_offset = 5[ppm]
Clipped = FALSE
Mod_return = 1
Scans = 32
Total_scans = 32
X_90_width
X_acq_time
X_angle = 45[deg]
X_atn
X_pulse
Irr_mode = Off
Tri_mode = Off
Dante_presat = FALSE
Initial_wait = 1[s]
Recvr_gain
Relaxation_delay
Repetition_time
Temp_get

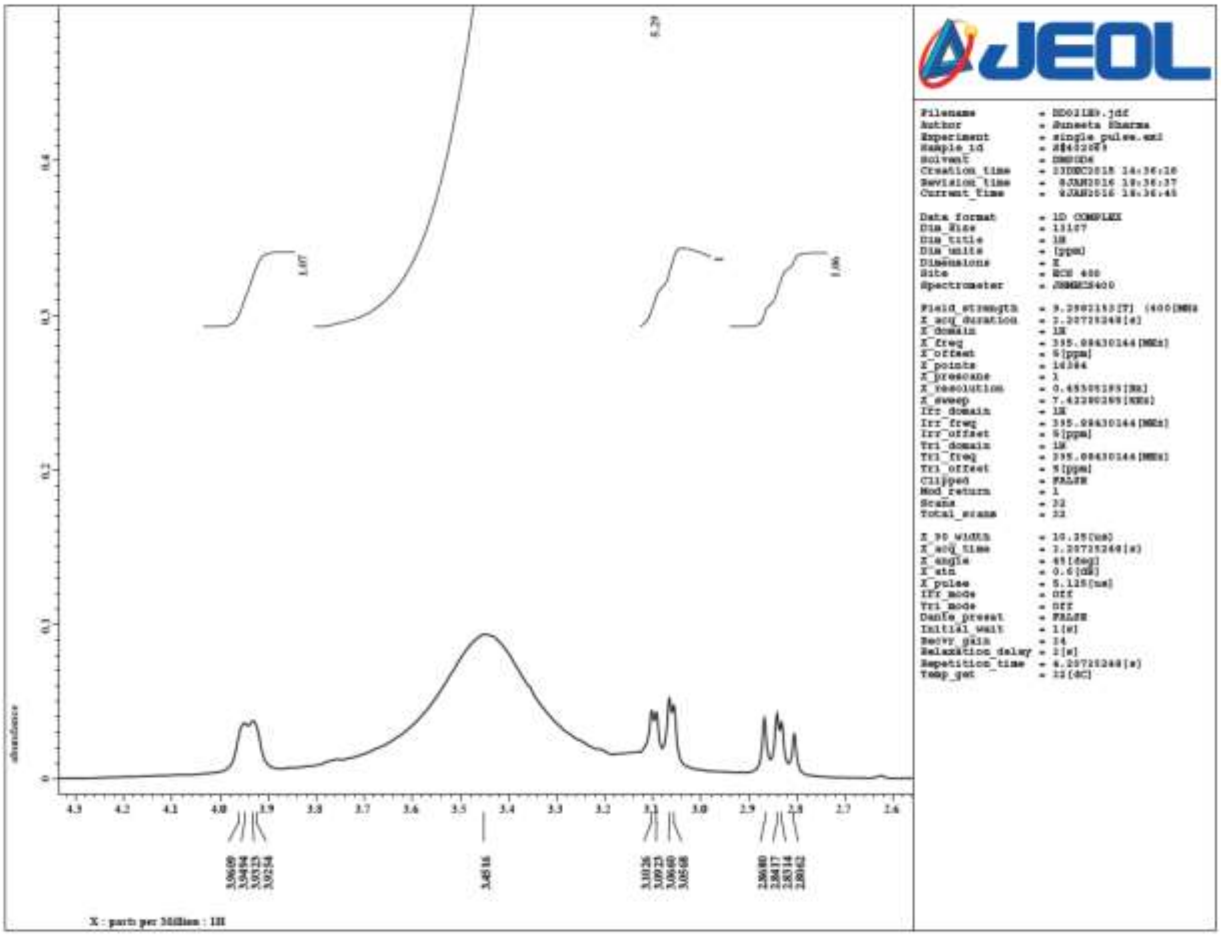

JEOL
Filename = DD021E0.jdf
Author = Suneeta Sharma
Experiment = single_pulse.exl
Solvent = DMSO-D6
Creation_time = 23DEC2015 14:36:16
Revision_time = 8JAN2016 18:36:37
Current_time = 9JAN2016 18:36:45
Data_format = 1D COMPLEX
Dim_size = 13107
Dim_title = 1H
Dim_units = [ppm]
Dimensions = X
Site = ECS 400
Field_strength = 9.3902153[T] (400[MHz
X_acq_duration = 2.20725248[s]
X_domain = 1H
X_freq = 395.88430144[MHz]
X_offset = 5[ppm]
X_points = 16384
X_prescans = 1
X_resolution = 0.45305185[Hz]
X_sweep = 7.42280285[KHz]
Irr_domain = 1H
Irr_freq = 395.88430144[MHz]
Irr_offset = 5[ppm]
Tri_domain = 1H
Tri_freq = 395.88430144[MHz]
Tri_offset = 5[ppm]
Clipped = FALSE
Mod_return = 1
Scans = 32
Total_scans = 32
X_90_width = 10.25[us]
X_acq_time = 2.20725248[s]
X_angle = 45[deg]
X_atn = 0.6[dB]
X_pulse = 5.125[us]
Irr_mode = Off
Tri_mode = Off
Dante_presat = FALSE
Initial_wait = 1[s]
Recvr_gain = 24
Relaxation_delay = 2[s]
Repetition_time = 4.20725248[s]
Temp_get = 22[dC]
1.07
5.29
1.06
3.9609
3.9494
3.9323
3.9234
3.4516
3.1026
3.0923
3.0640
3.0548
2.8680
2.8417
2.8314
2.8062
abundance
X : parts per Million : 1H

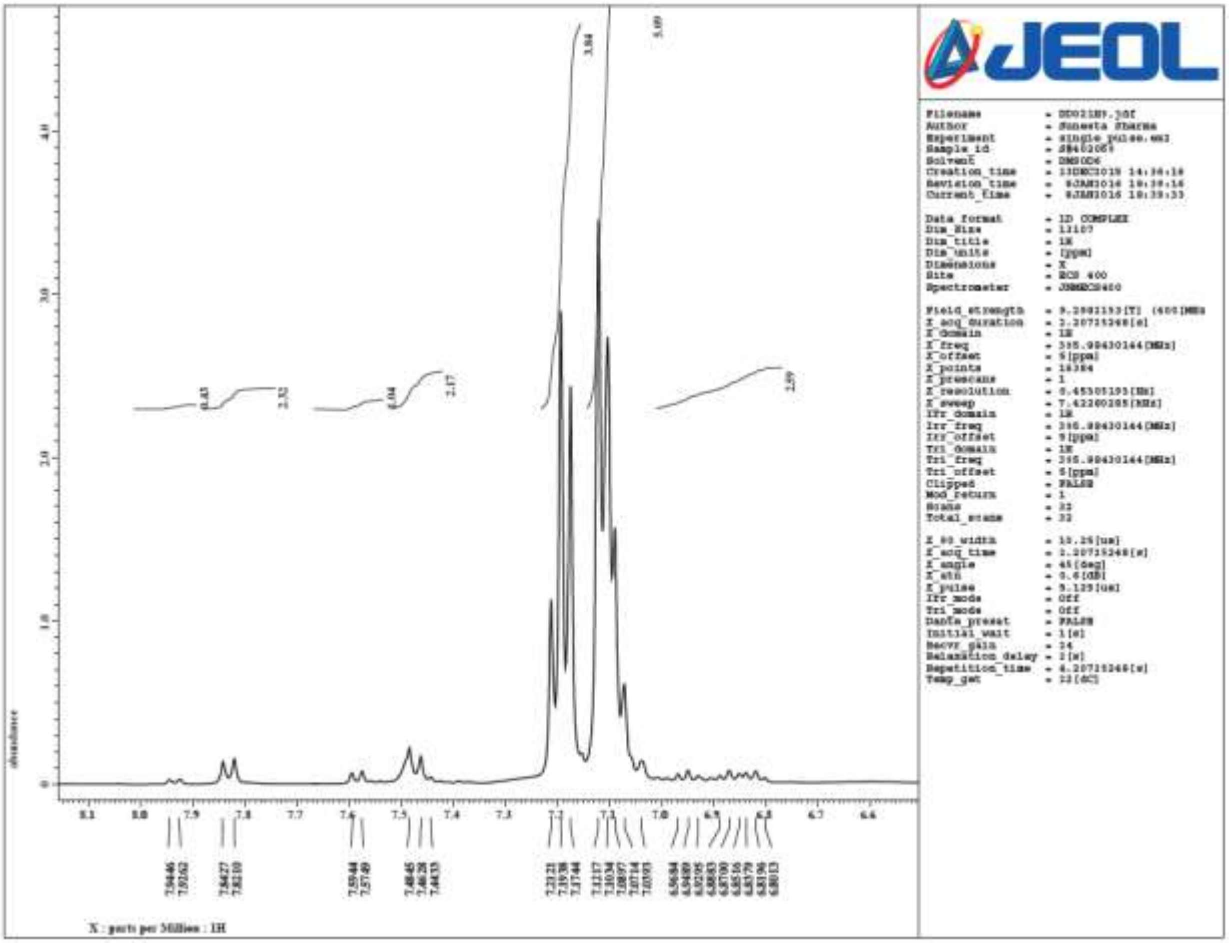

17d

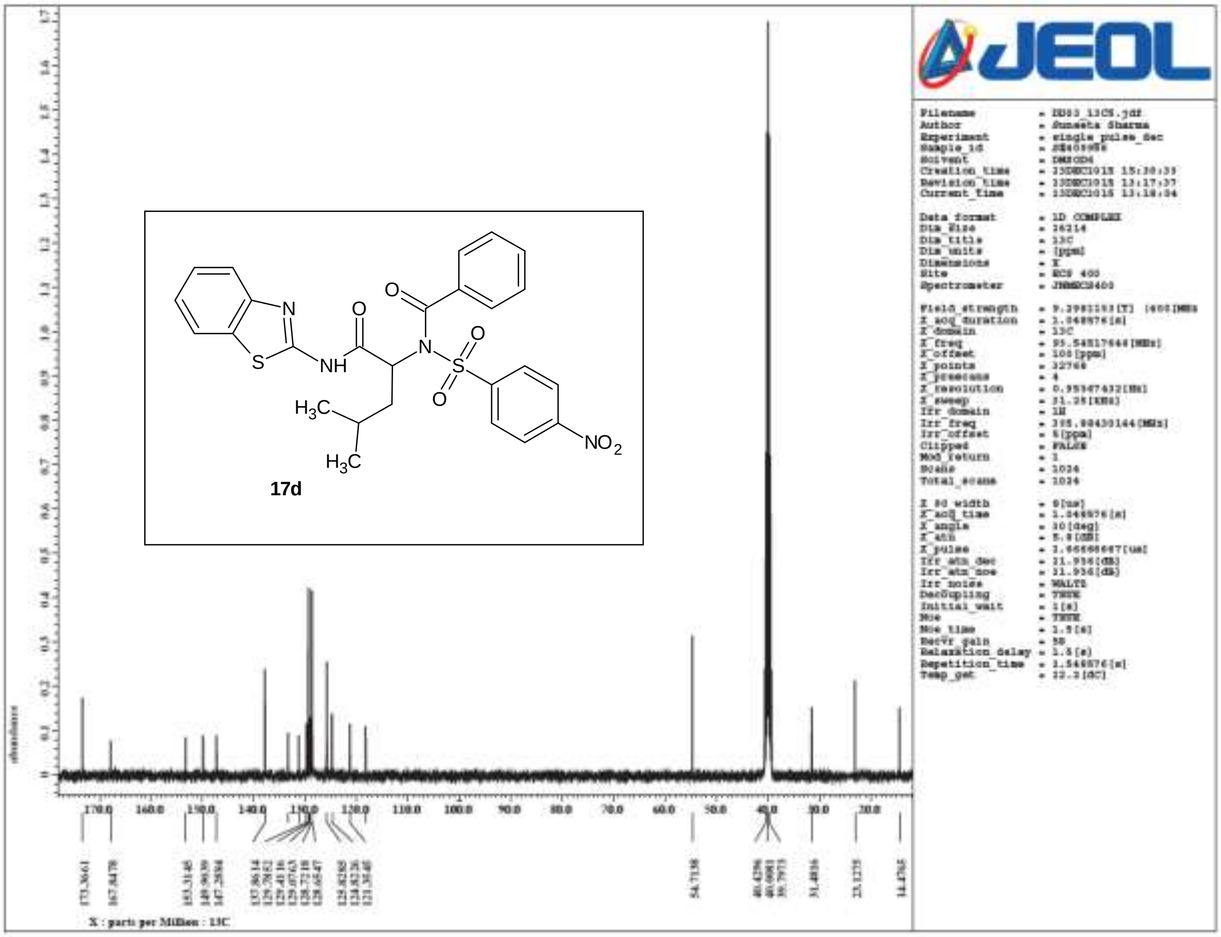

N
O
O
S
NH
N
O
S
O
H_3C
H_3C
NO_2
17d
abundance
173.3661
167.8478
153.3145
149.9039
147.2884
137.3614
129.7852
129.4116
129.0763
128.7218
128.6547
125.8288
124.8236
121.3048
54.7138
40.4296
40.0081
39.7973
31.4816
23.1275
14.4765
X : parts per Million : 13C

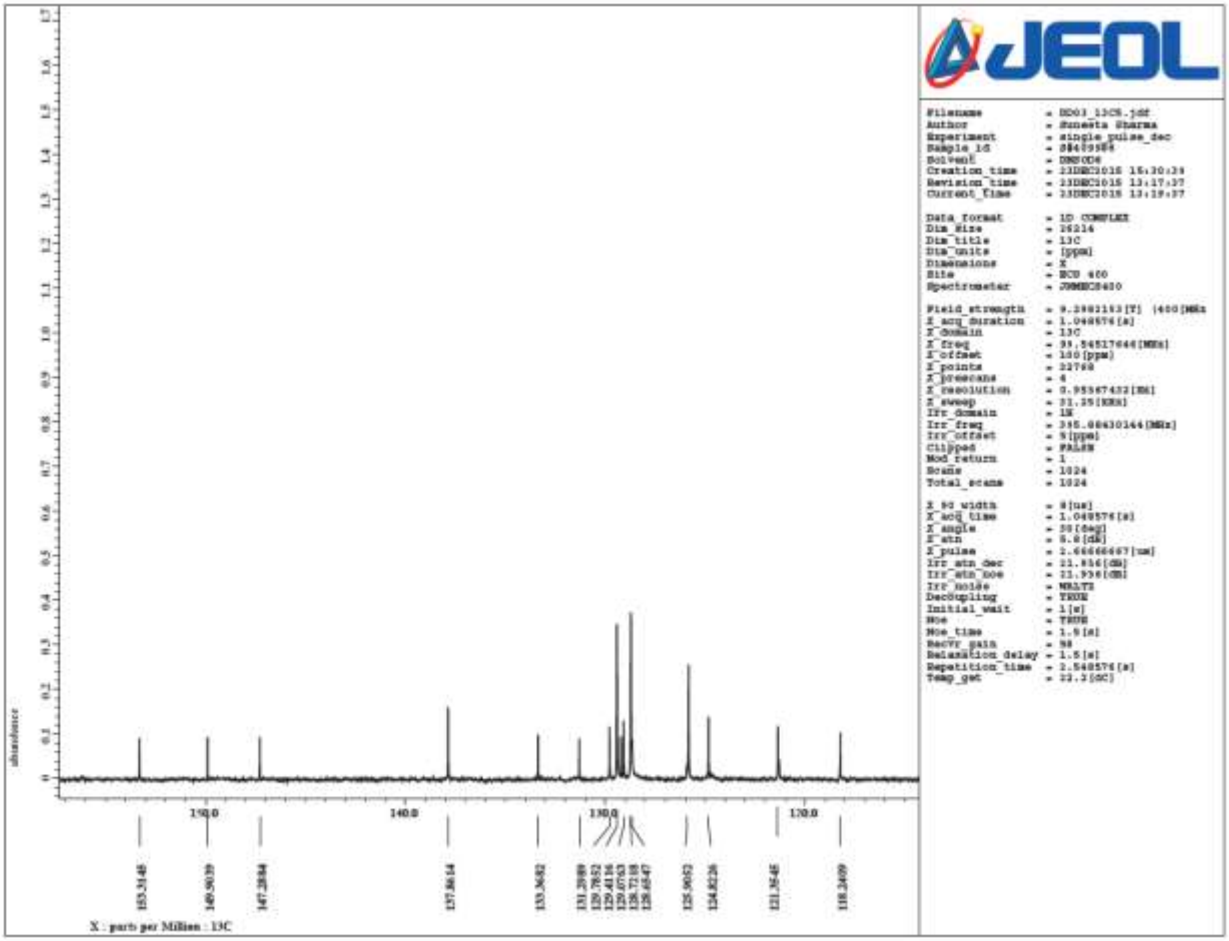
abundance
150.0
140.0
130.0
120.0
153.3148
149.9039
147.2884
137.8614
133.3682
131.2989
129.7852
129.4116
129.0763
128.7218
128.6547
125.9052
124.8226
121.3546
118.2409
X : parts per Million : 13C
JEOL
Filename = DD03_13CS.jdf
Author = Suneeta Sharma
Experiment = single_pulse_dec
Solvent = DMSOD6
Data_format = 1D COMPLEX
Dim_title = 13C
Dim_units = [ppm]
Dimensions = X
Site = ECS 400
X_domain = 13C
X_offset = 100[ppm]
X_points = 32768
X_prescans = 4
Irr_domain = 1H
Irr_offset = 5[ppm]
Clipped = FALSE
Mod_return = 1
Scans = 1024
Total_scans = 1024
X_90_width = 8[us]
X_acq_time = 1.048576[s]
X_angle = 30[deg]
Decoupling = TRUE
Initial_wait = 1[s]
Noe = TRUE
Noe_time = 1.5[s]
Relaxation_delay = 1.5[s]
Repetition_time = 2.548576[s]

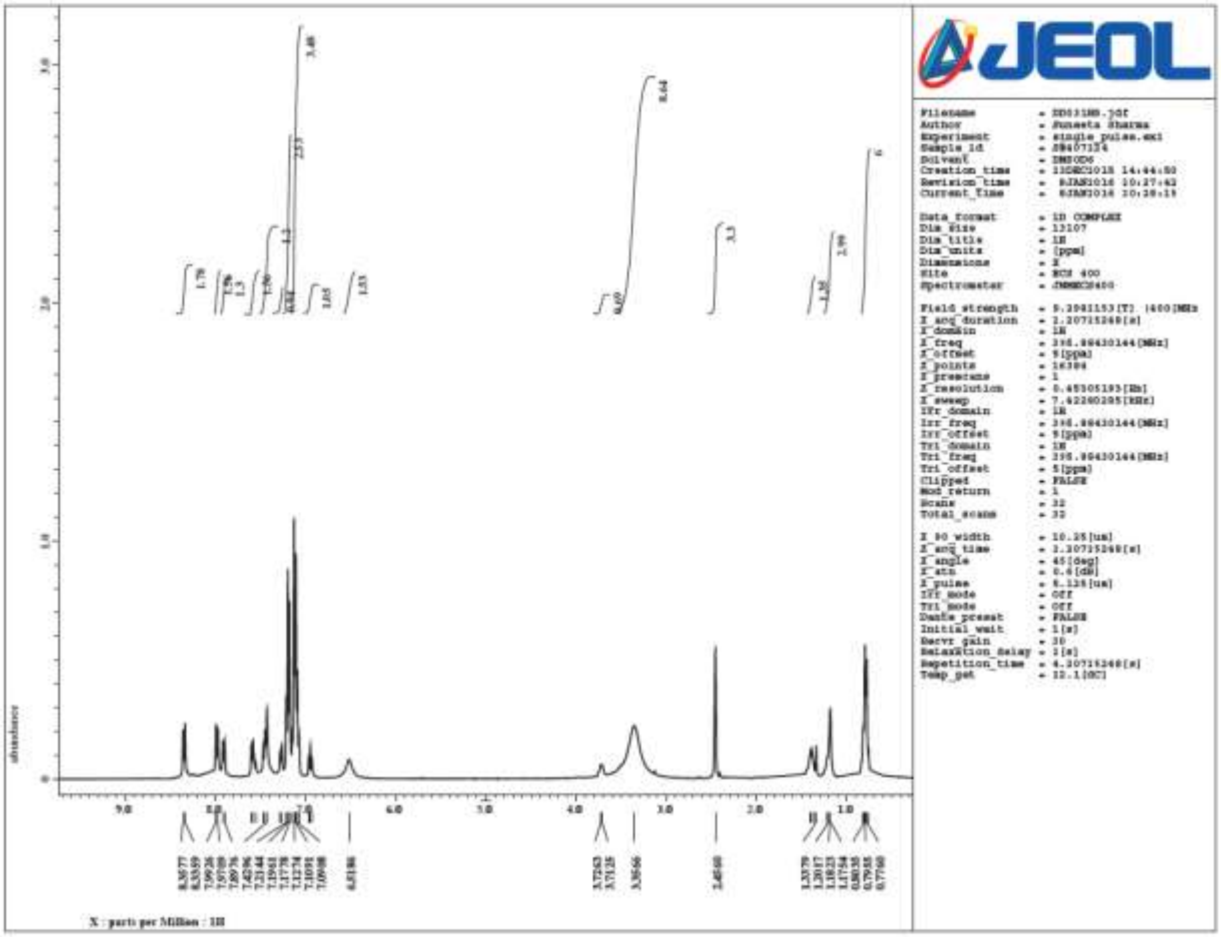
JEOL
Filename
Author
Experiment
Sample_id
Solvent
Creation_time
Revision_time
Current_time
Data_format = 1D COMPLEX
Dim_size
Dim_title = 1H
Dim_units = [ppm]
Dimensions
Site
Spectrometer
Field_strength
X_acq_duration
X_domain = 1H
X_freq
X_offset = 5[ppm]
X_points = 16384
X_prescans = 1
X_resolution
X_sweep
Irr_domain = 1H
Irr_freq
Irr_offset = 5[ppm]
Tri_domain = 1H
Tri_freq
Tri_offset = 5[ppm]
Clipped = FALSE
Mod_return = 1
Scans = 32
Total_scans = 32
X_90_width
X_acq_time
X_angle = 45[deg]
X_atn
X_pulse
Irr_mode = Off
Tri_mode = Off
Dante_presat = FALSE
Initial_wait = 1[s]
Recvr_gain
Relaxation_delay
Repetition_time
Temp_get
abundance
X : parts per Million : 1H

17e

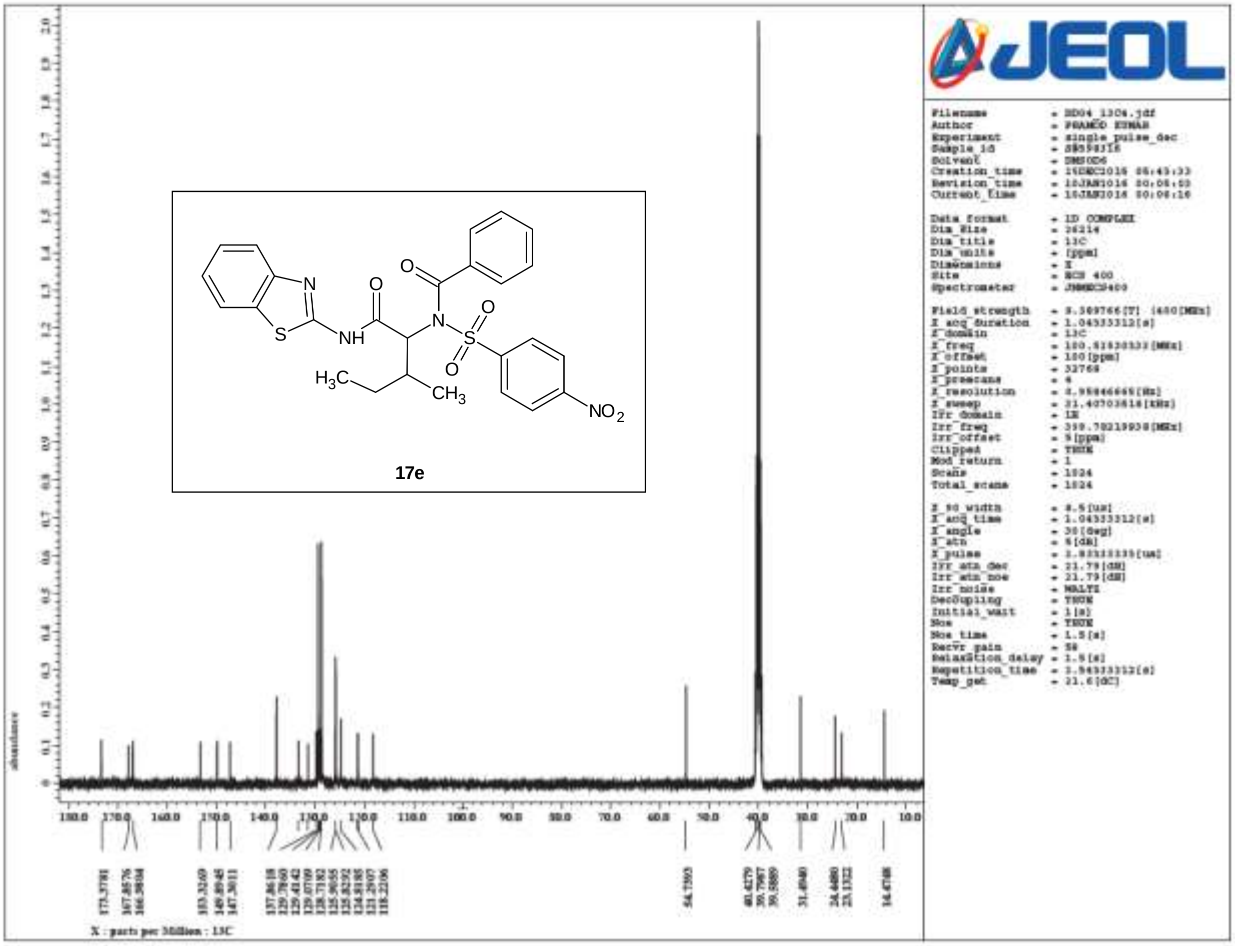
17e
N
S
NH
O
O
N
S
O
O
H_3C
CH_3
NO_2
abundance
X : parts per Million : 13C
173.3781
153.3269
149.8945
147.3011
137.8618
54.7393
40.4279
31.4940
14.4748
Filename = BD04_13C4.jdf
Experiment = single_pulse_dec
Solvent = DMSOD6
Dim_title = 13C
Dim_units = [ppm]
Site = ECS 400
X_domain = 13C
X_offset = 100[ppm]
X_points = 32768
Irr_domain = 1H
Scans = 1024
Total_scans = 1024
Irr_noise = WALTZ
Decoupling = TRUE
Noe = TRUE

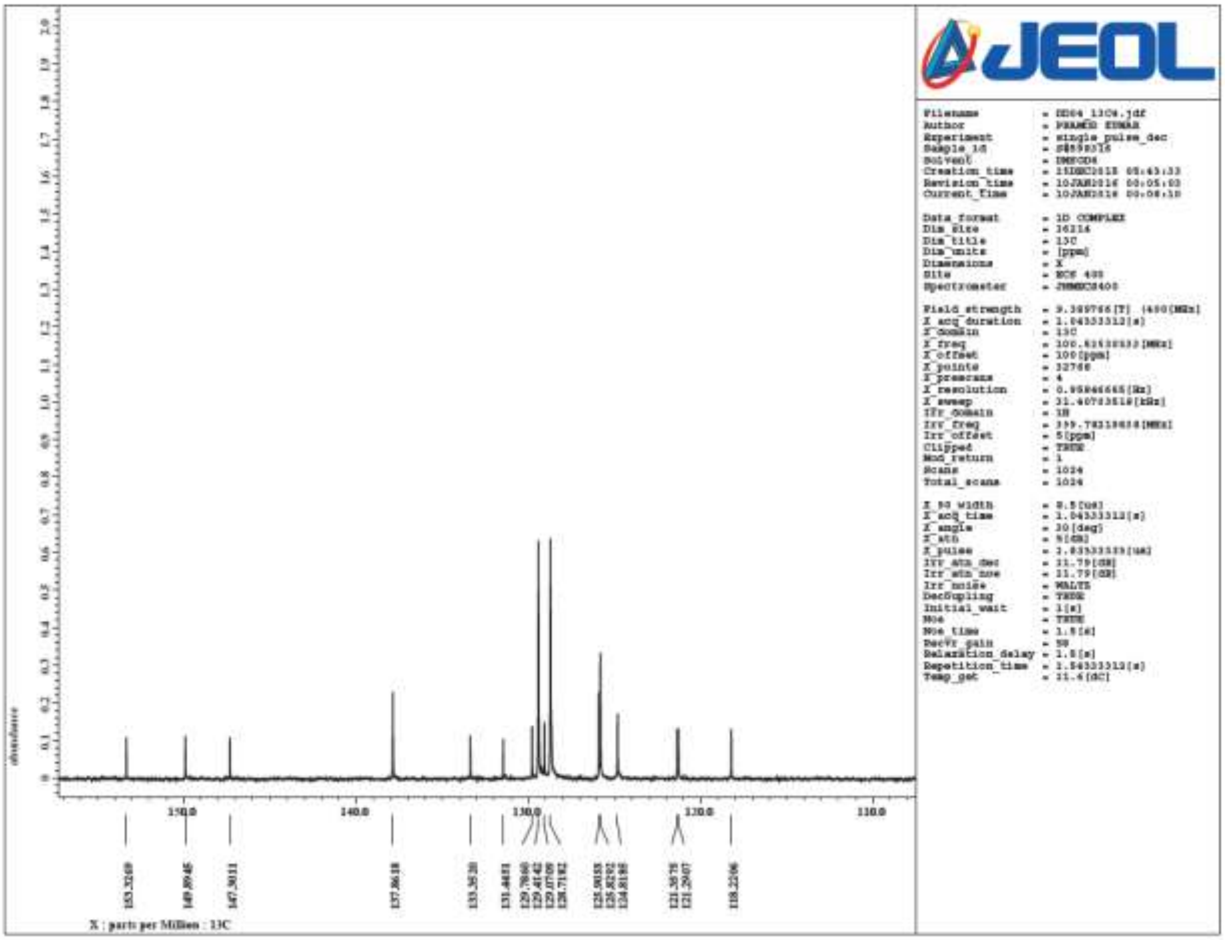
JEOL
Experiment = single_pulse_dec
Data_format = 1D COMPLEX
Dim_title = 13C
Dim_units = [ppm]
Dimensions = X
X_domain = 13C
X_offset = 100[ppm]
X_points = 32768
X_prescans = 4
Irr_domain = 1H
Irr_offset = 5[ppm]
Clipped = TRUE
Mod_return = 1
Scans = 1024
Total_scans = 1024
X_angle = 30[deg]
Irr_noise = WALTZ
Decoupling = TRUE
Noe = TRUE
abundance
150.0
140.0
130.0
120.0
110.0
153.3269
149.8946
147.3011
137.8618
133.3530
131.4481
129.7846
129.4142
129.0709
128.7182
125.9088
125.8292
124.8198
121.3575
121.2907
118.2206
X : parts per Million : 13C

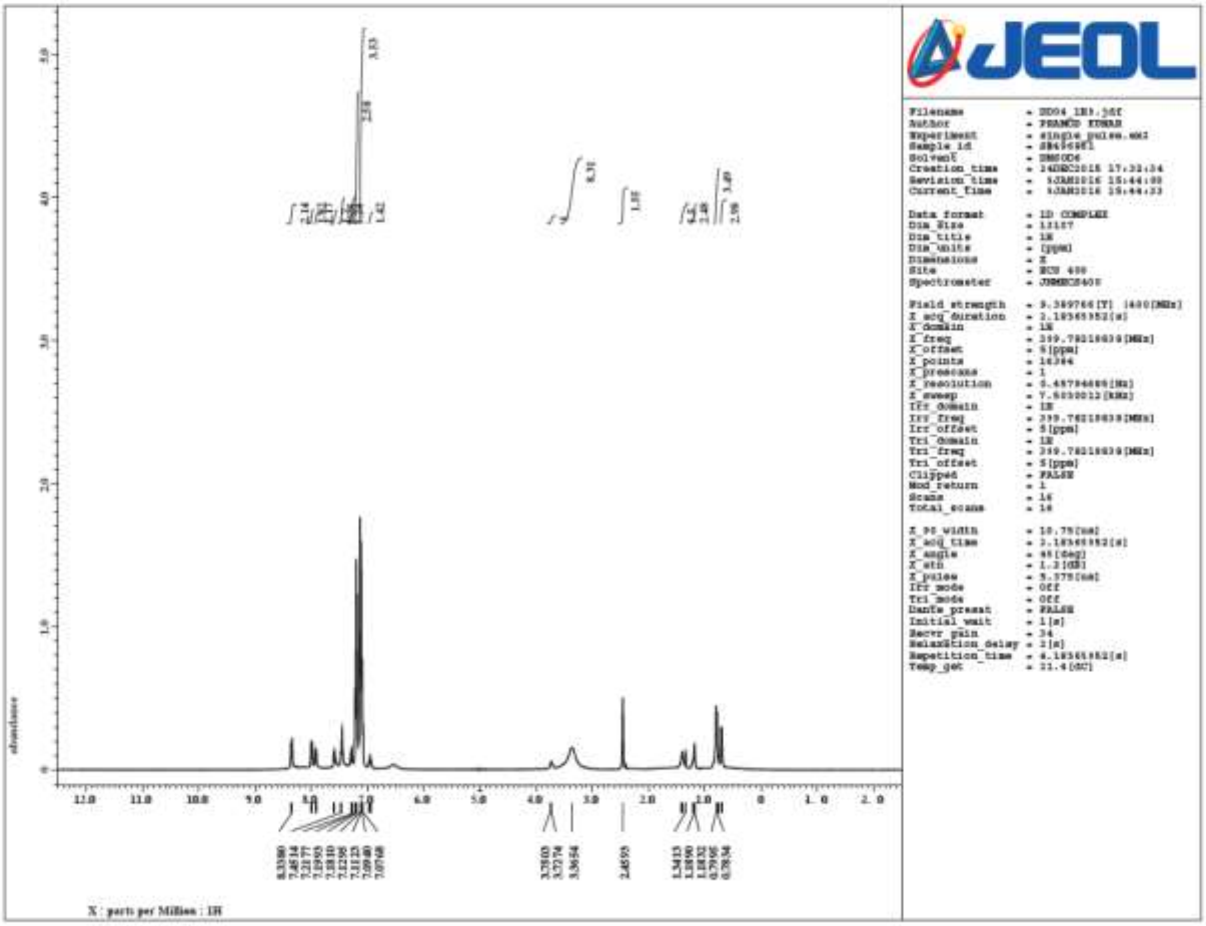
JEOL
Filename
Author
Experiment
Sample_id
Solvent = DMSOD6
Creation_time
Revision_time
Current_time
Data format = 1D COMPLEX
Dim_size
Dim_title = 1H
Dim_units = [ppm]
Dimensions = X
Site = ECS 400
Spectrometer
Field_strength
X_acq_duration
X_domain = 1H
X_freq
X_offset = 5[ppm]
X_points = 16384
X_prescans = 1
X_resolution
X_sweep
Irr_domain = 1H
Irr_freq
Irr_offset = 5[ppm]
Tri_domain = 1H
Tri_freq
Tri_offset = 5[ppm]
Clipped = FALSE
Mod_return = 1
Scans = 16
Total_scans = 16
X_90_width
X_acq_time
X_angle = 45[deg]
X_atn
X_pulse
Irr_mode = Off
Tri_mode = Off
Dante_presat = FALSE
Initial_wait = 1[s]
Recvr_gain = 34
Relaxation_delay
Repetition_time
Temp_get
abundance
X : parts per Million : 1H

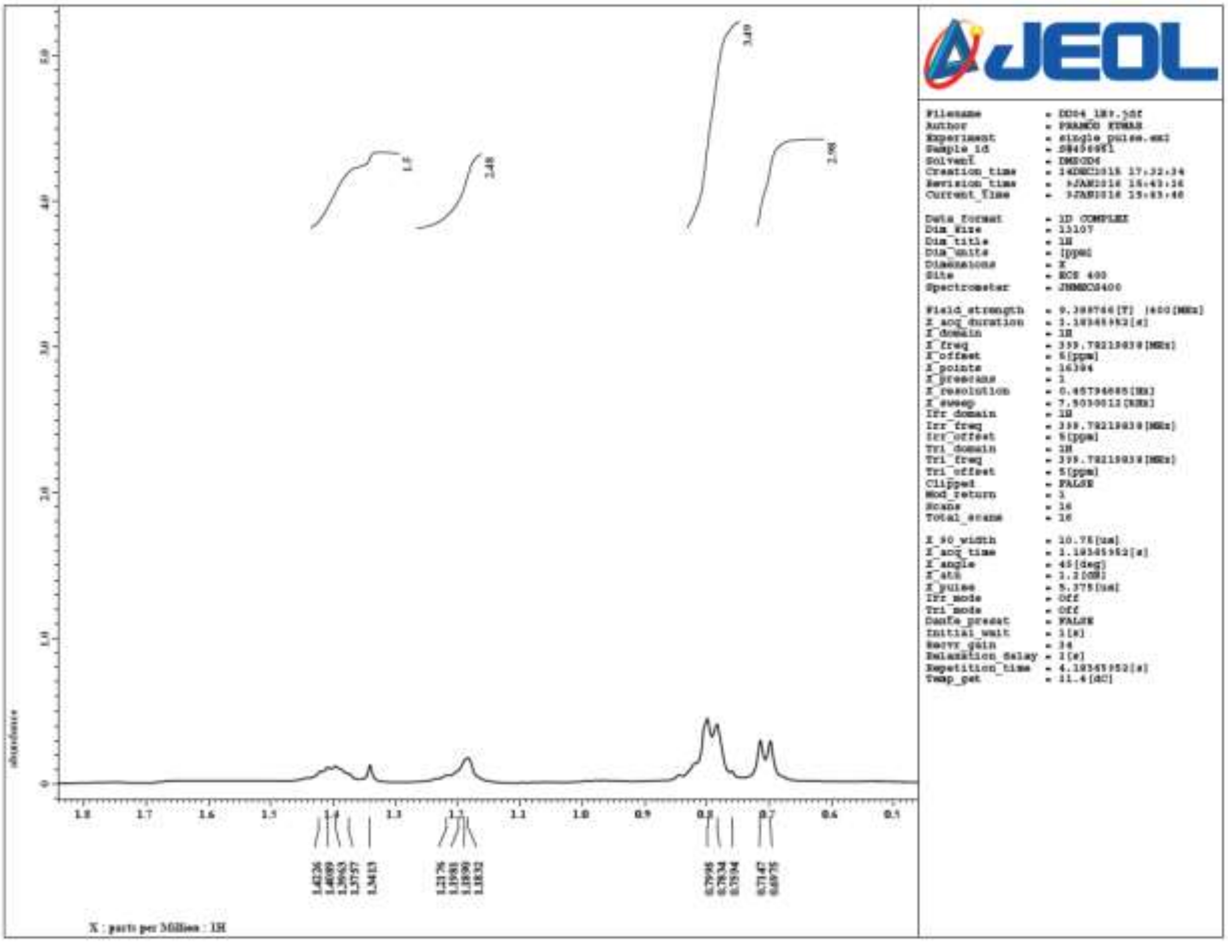

JEOL
Filename = DD04_1H9.jdf
Author = FRANCO ETMAS
Experiment = single_pulse.exp
Sample_id = 08496981
Solvent = DMSOD6
Creation_time = 14DEC2015 17:32:34
Revision_time = 9JAN2016 15:43:16
Current_Time = 9JAN2016 15:43:46
Data_format = 1D COMPLEX
Dim_size = 13107
Dim_title = 1H
Dim_units = [ppm]
Dimensions = X
Site = ECS 400
Spectrometer = JNMECS400
Field_strength = 9.389766[T] (400[MHz]
X_acq_duration = 2.18365952[s]
X_domain = 1H
X_freq = 399.78219838[MHz]
X_offset = 5[ppm]
X_points = 16384
X_prescans = 1
X_resolution = 0.45794685[Hz]
X_sweep = 7.5030012[kHz]
Irr_domain = 1H
Irr_freq = 399.78219838[MHz]
Irr_offset = 5[ppm]
Tri_domain = 1H
Tri_freq = 399.78219838[MHz]
Tri_offset = 5[ppm]
Clipped = FALSE
Mod_return = 1
Scans = 16
Total_scans = 16
X_90_width = 10.75[us]
X_acq_time = 2.18365952[s]
X_angle = 45[deg]
X_atn = 1.2[dB]
X_pulse = 5.375[us]
Irr_mode = Off
Tri_mode = Off
Dante_presat = FALSE
Initial_wait = 1[s]
Recvr_gain = 34
Relaxation_delay = 2[s]
Repetition_time = 4.18365952[s]
Temp_get = 21.4[dC]
abundance
5.0
4.0
3.0
2.0
1.0
0
1.5
2.48
3.49
2.98
1.8 1.7 1.6 1.5 1.4 1.3 1.2 1.1 1.0 0.9 0.8 0.7 0.6 0.5
1.4226
1.4089
1.3963
1.3757
1.3413
1.2176
1.1981
1.1890
1.1832
0.7998
0.7834
0.7594
0.7147
0.6975
X : parts per Million : 1H

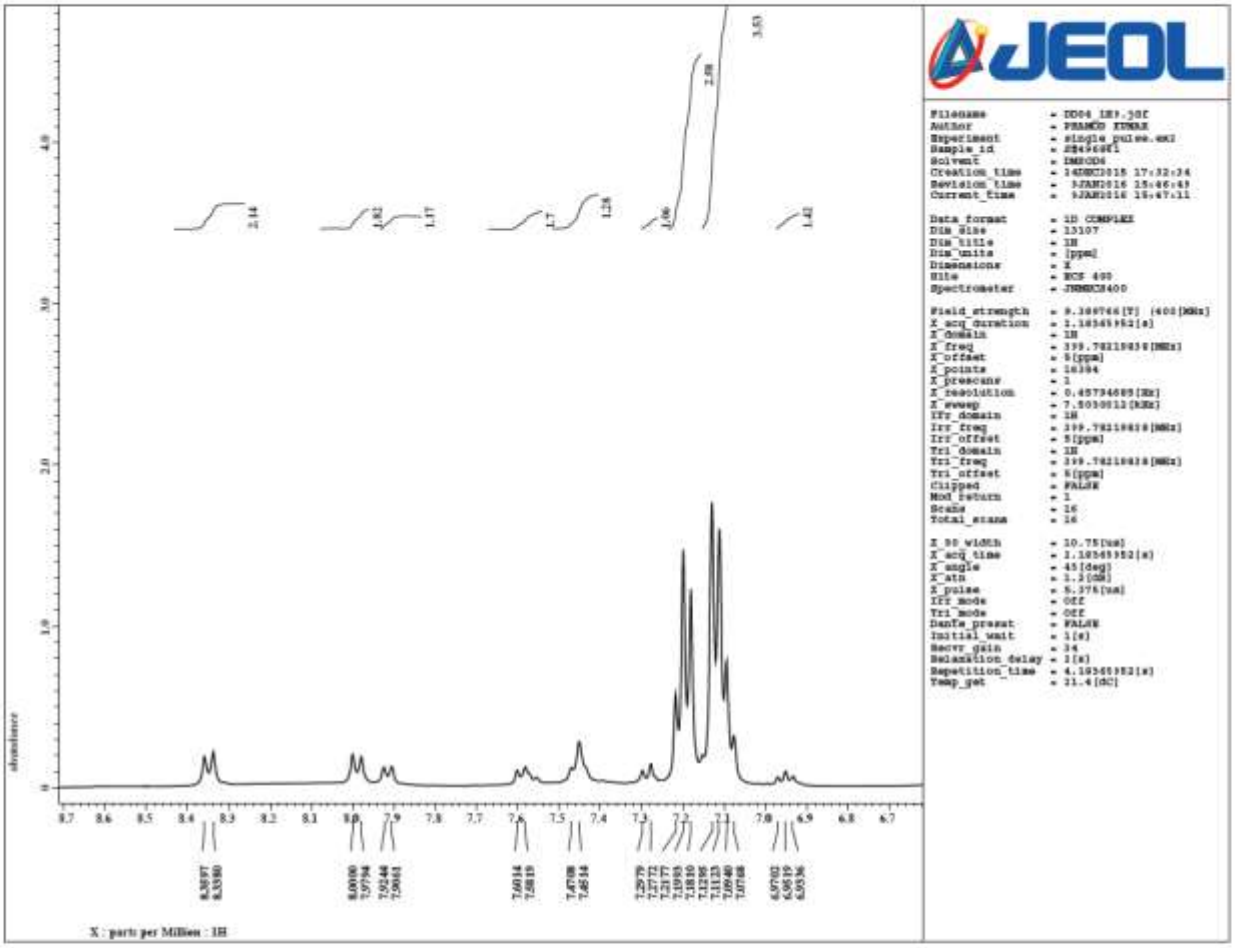

17f

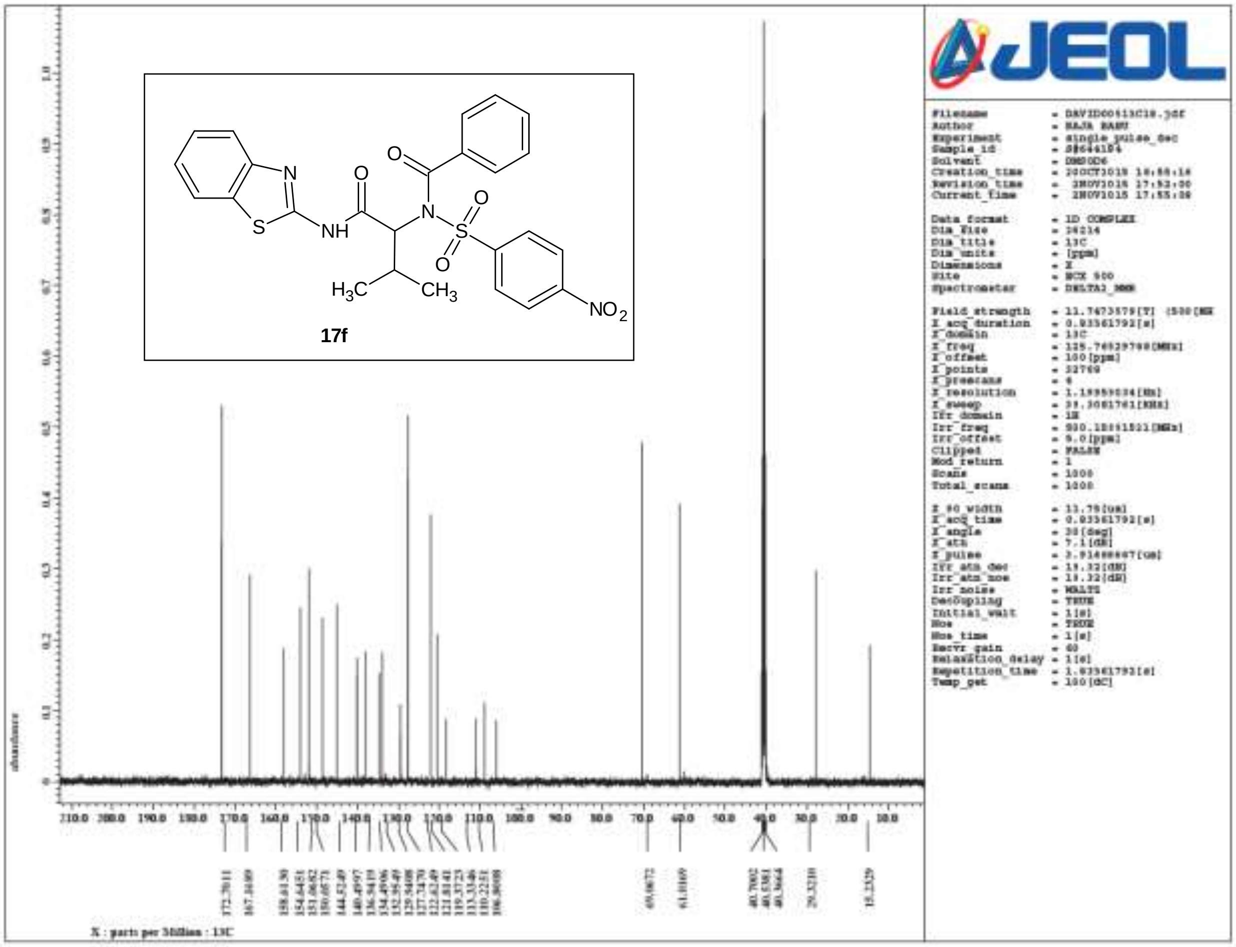

N
S
NH
O
O
N
S
O
O
H3C
CH3
NO2
17f
abundance
X : parts per Million : 13C

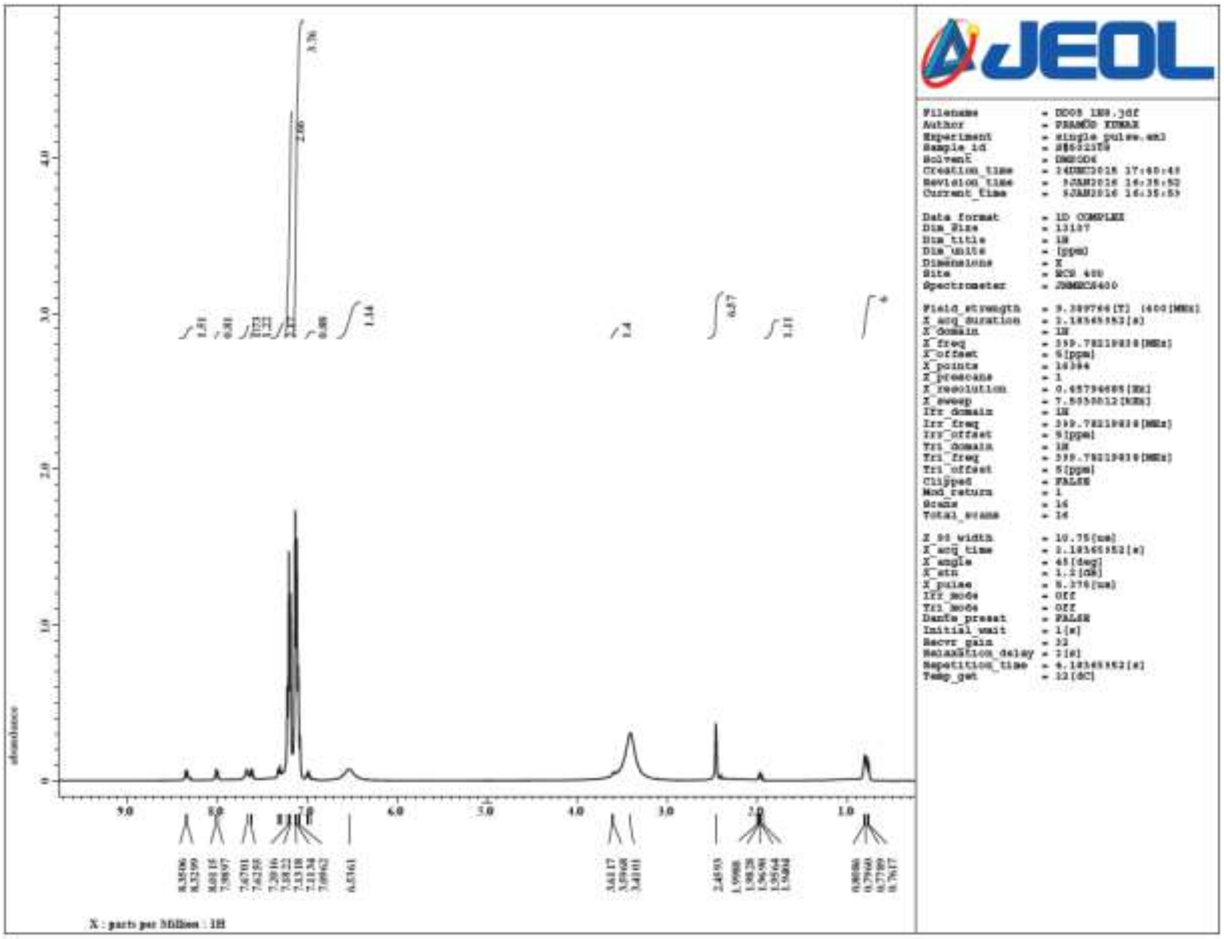

JEOL
Experiment = single_pulse.ex2
Data_format = 1D COMPLEX
Dim_title = 1H
Dim_units = [ppm]
Dimensions = X
Site = ECS 400
Field_strength = 9.389766[T] (400[MHz])
X_domain = 1H
X_offset = 5[ppm]
X_points = 16384
X_prescans = 1
Irr_domain = 1H
Irr_offset = 5[ppm]
Tri_domain = 1H
Tri_offset = 5[ppm]
Clipped = FALSE
Mod_return = 1
Scans = 16
Total_scans = 16
X_angle = 45[deg]
Irr_mode = Off
Tri_mode = Off
Dante_presat = FALSE
Initial_wait = 1[s]
Recvr_gain = 32
abundance
X : parts per Million : 1H

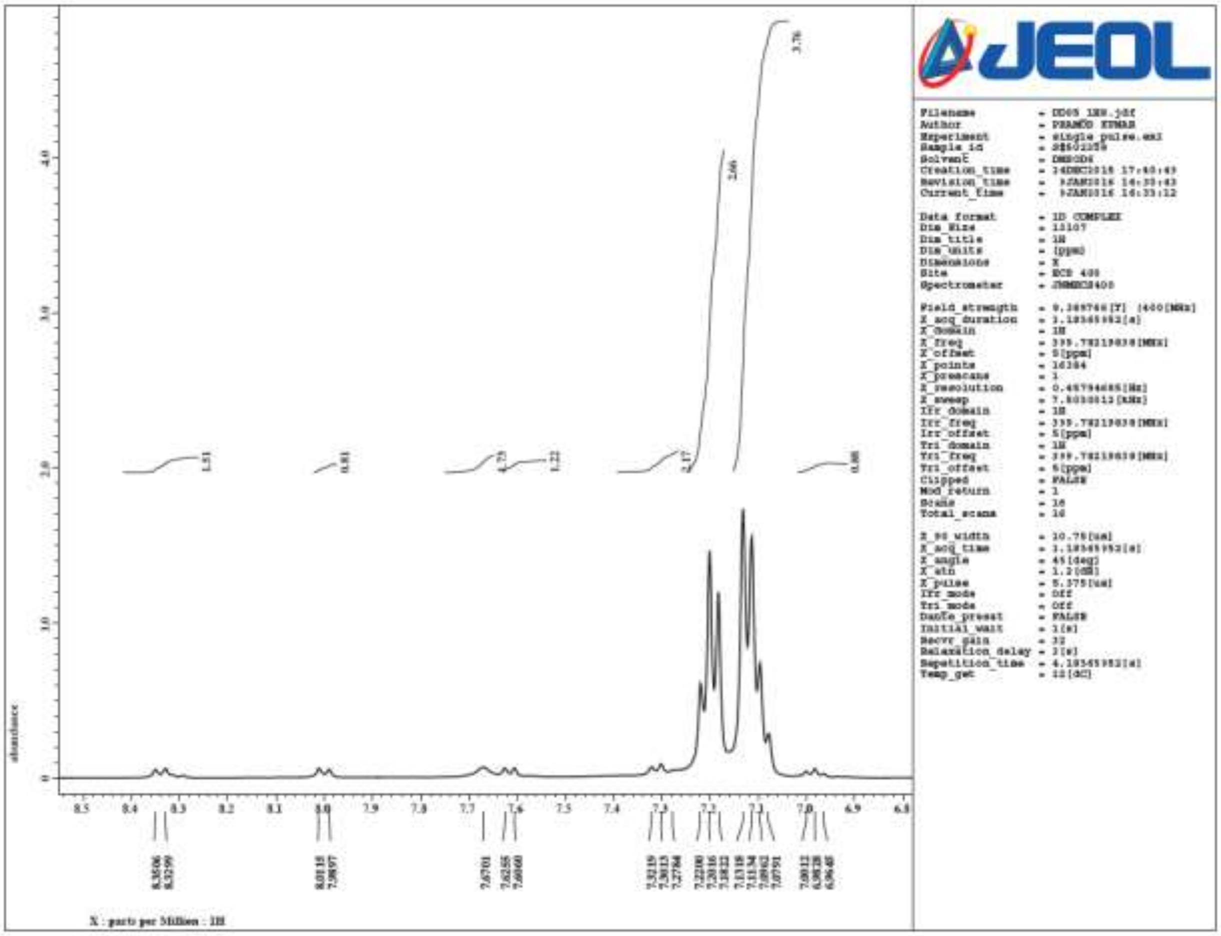

17g

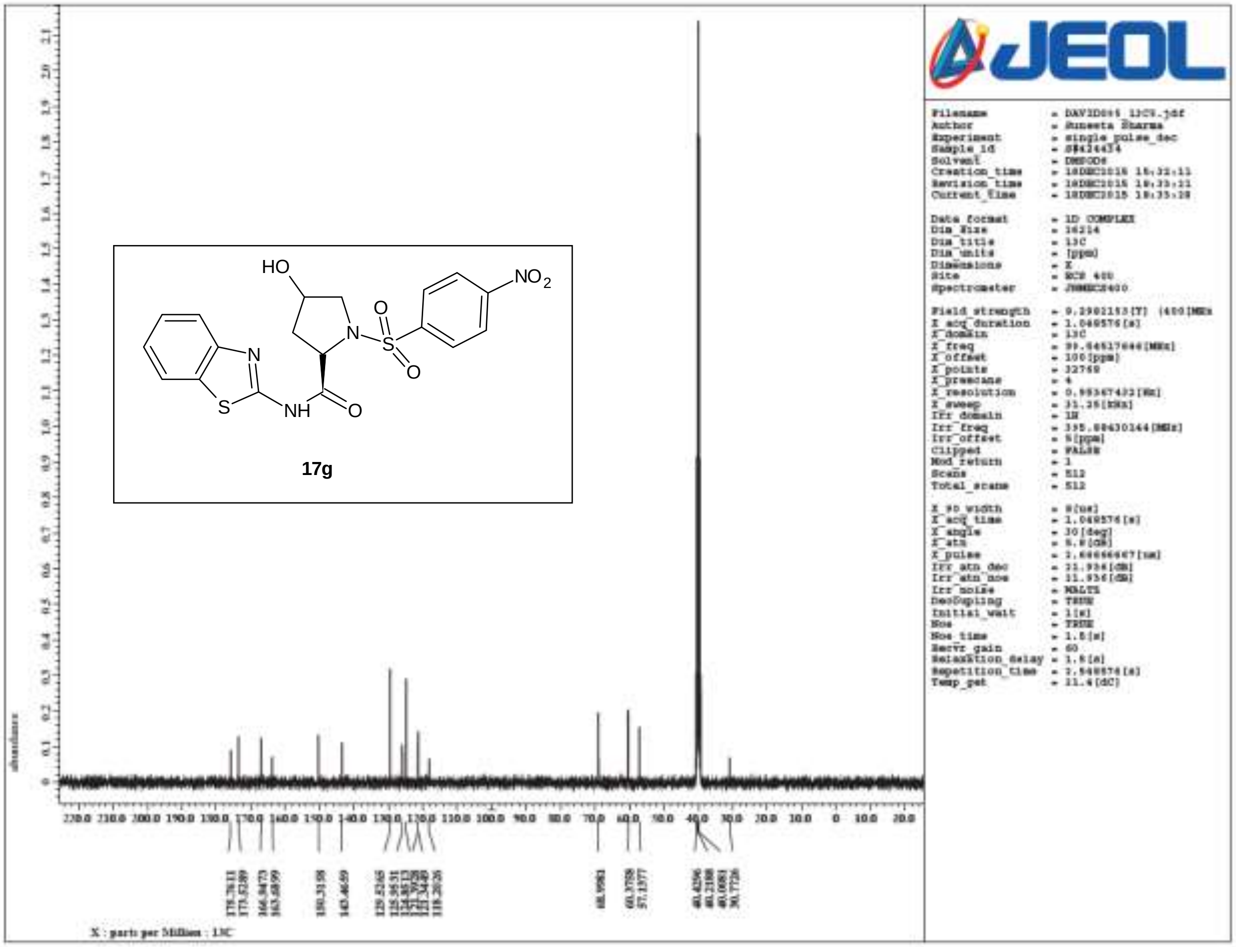
HO
NO2
N
O
S
O
N
S
NH
O
17g
abundance
X : parts per Million : 13C
175.7611
173.5289
166.9473
163.6899
150.3158
143.4659
129.5265
118.2026
68.9983
60.3758
57.1377
40.4256
40.2188
40.0081
30.7726
JEOL
Filename
Author
Experiment = single_pulse_dec
Solvent
Creation_time
Revision_time
Current_time
Data_format = 1D COMPLEX
Dim_title = 13C
Dim_units = [ppm]
Dimensions = X
Spectrometer
Field_strength
X_acq_duration = 1.048576[s]
X_domain = 13C
X_offset = 100[ppm]
X_points = 32768
X_prescans = 4
Irr_domain = 1H
Irr_offset = 5[ppm]
Clipped = FALSE
Mod_return = 1
Scans = 512
Total_scans = 512
X_acq_time = 1.048576[s]
X_angle = 30[deg]
Irr_noise = WALTZ
Decoupling = TRUE
Initial_wait = 1[s]
Noe = TRUE
Noe_time = 1.5[s]
Recvr_gain = 60
Relaxation_delay = 1.5[s]
Temp_get

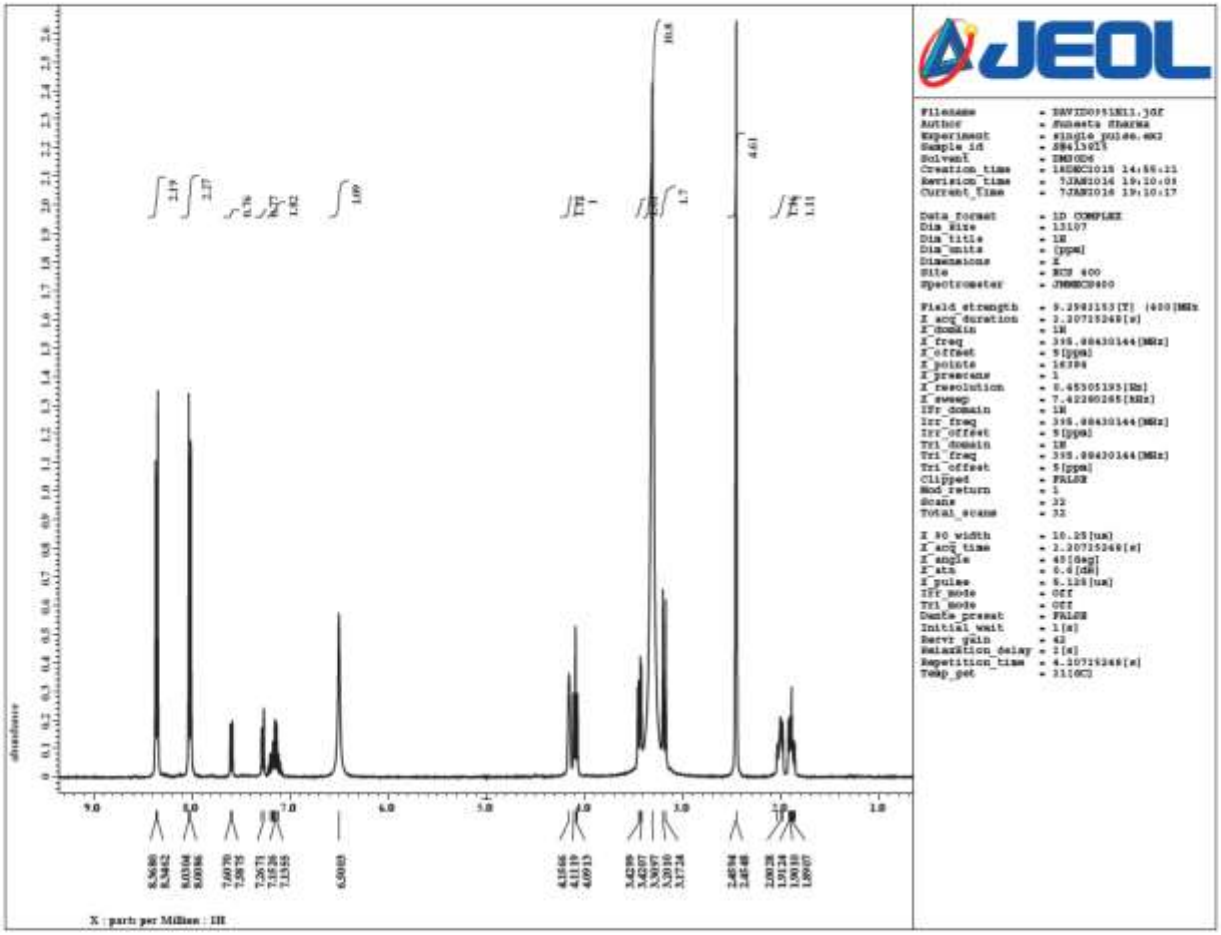

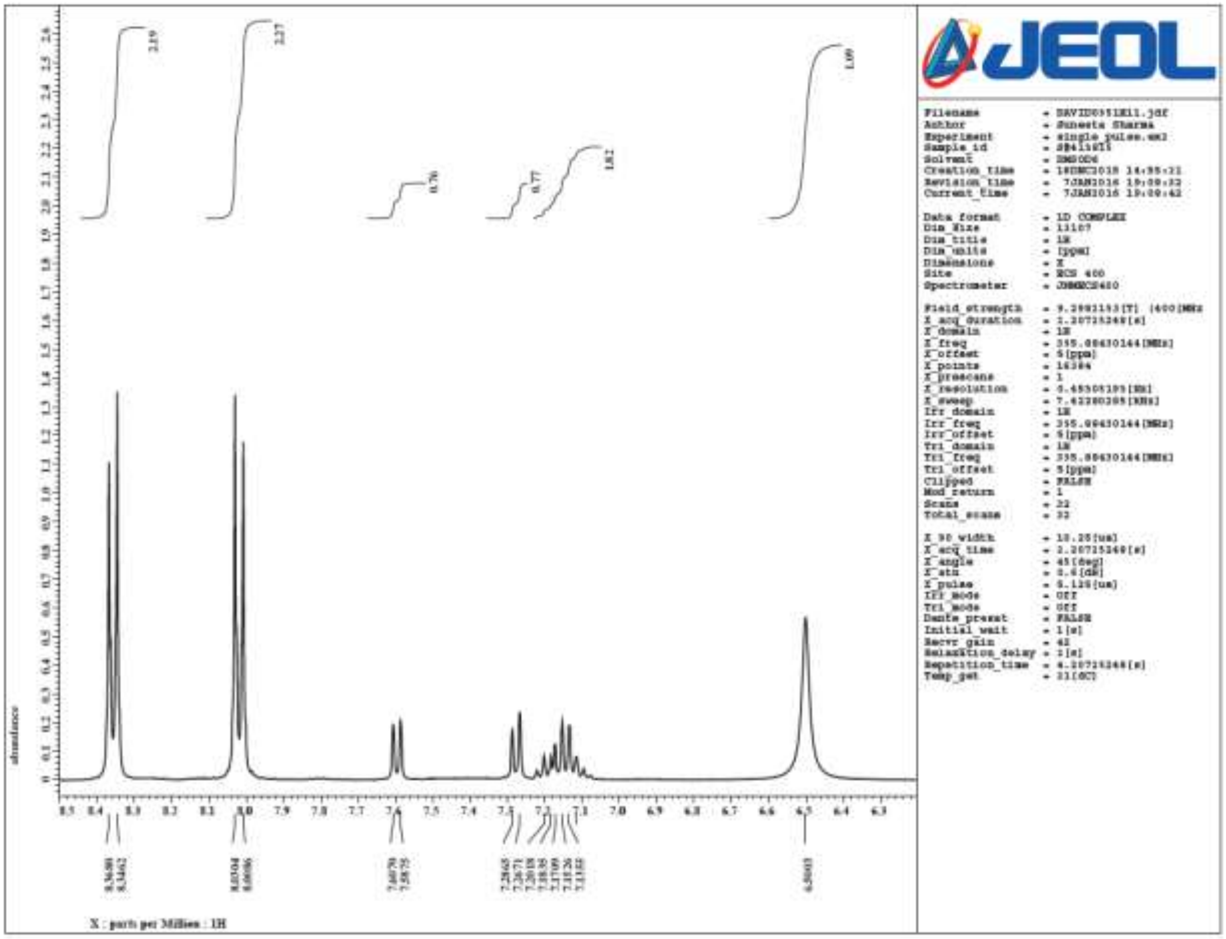
JEOL
Filename = DAVID0351E11.jdf
Author = Suneeta Sharma
Experiment = single_pulse.ex2
Solvent = DMSO-D6
Creation_time = 18DEC2015 14:55:21
Revision_time = 7JAN2016 19:09:32
Current_time = 7JAN2016 19:09:42
Data_format = 1D COMPLEX
Dim_title = 1H
Dim_units = [ppm]
Dimensions = X
Site = ECS 400
Field_strength = 9.3983153[T] (400[MHz
X_domain = 1H
X_offset = 5[ppm]
X_points = 16384
X_prescans = 1
Irr_domain = 1H
Irr_offset = 5[ppm]
Tri_domain = 1H
Tri_offset = 5[ppm]
Clipped = FALSE
Mod_return = 1
Scans = 32
Total_scans = 32
X_angle = 45[deg]
Irr_mode = Off
Tri_mode = Off
Dante_presat = FALSE
Initial_wait = 1[s]
Recvr_gain = 42
Relaxation_delay = 1[s]
Temp_get = 21[dC]
2.19
2.27
0.76
0.77
1.82
1.09
8.3690
8.3462
8.0304
8.0086
7.6079
7.5875
6.5003
abundance
X : parts per Million : 1H

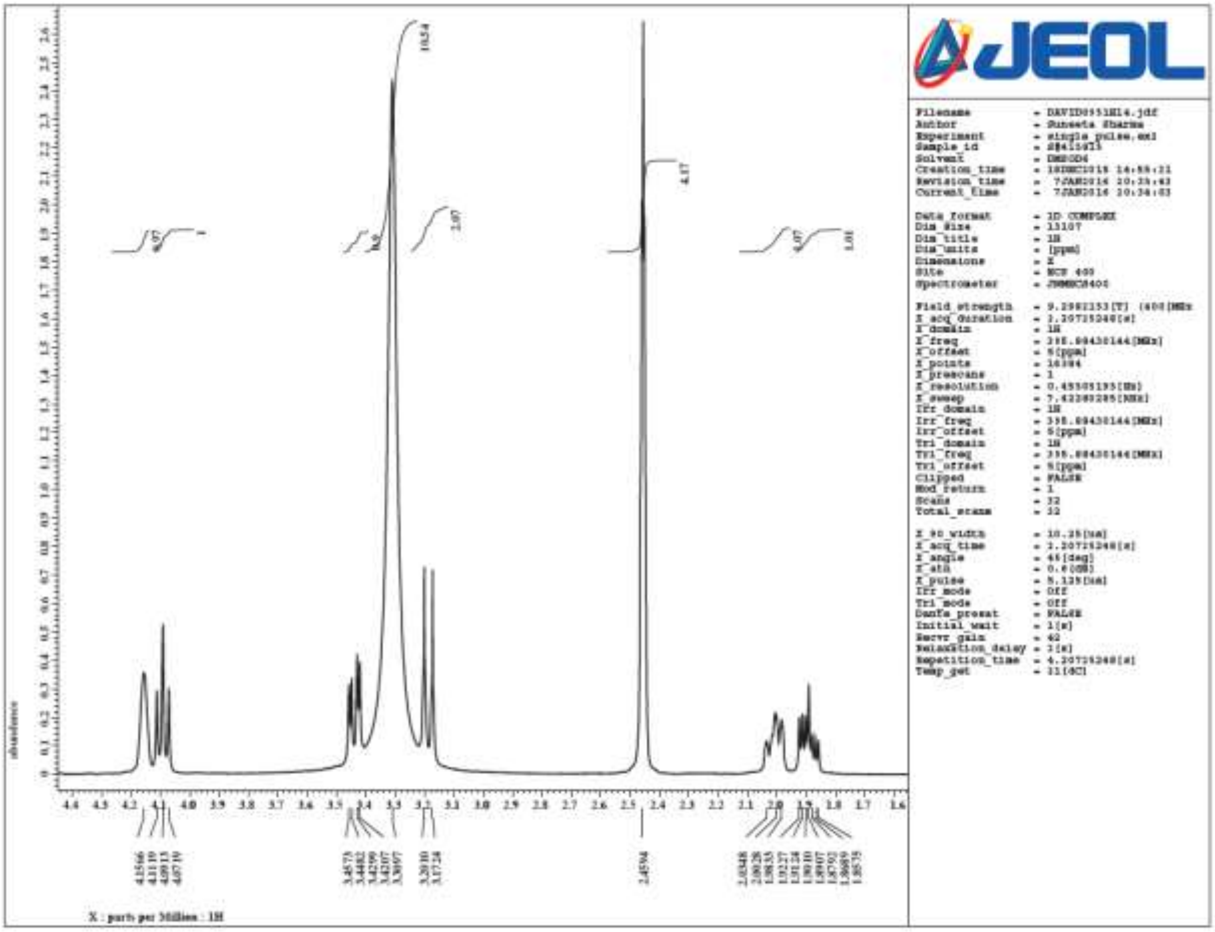
JEOL
Filename = DAVID0551H14.jdf
Author = Suneeta Sharma
Experiment = single_pulse.ex2
Solvent = DMSOD4
Data_format = 1D COMPLEX
Dim_title = 1H
Dim_units = [ppm]
Field_strength = 9.3982153[T] (400[MHz
X_offset = 5[ppm]
X_points = 16384
X_prescans = 1
X_resolution = 0.45505193[Hz]
X_sweep = 7.45280285[kHz]
Irr_domain = 1H
Irr_offset = 5[ppm]
Tri_domain = 1H
Tri_offset = 5[ppm]
Clipped = FALSE
Mod_return = 1
Scans = 32
Total_scans = 32
X_angle = 45[deg]
Irr_mode = Off
Tri_mode = Off
Dante_presat = FALSE
Initial_wait = 1[s]
Recvr_gain = 42
Relaxation_delay = 3[s]
Temp_get = 21[dC]
abundance
10.54
4.17
2.07
1.01
4.1566
4.1119
4.0913
4.0719
3.4573
3.4482
3.4299
3.4207
3.3097
3.2010
3.1724
2.4594
2.0348
2.0028
1.9835
1.9227
1.9124
1.9010
1.8907
1.8792
1.8689
1.8575
X : parts per Million : 1H

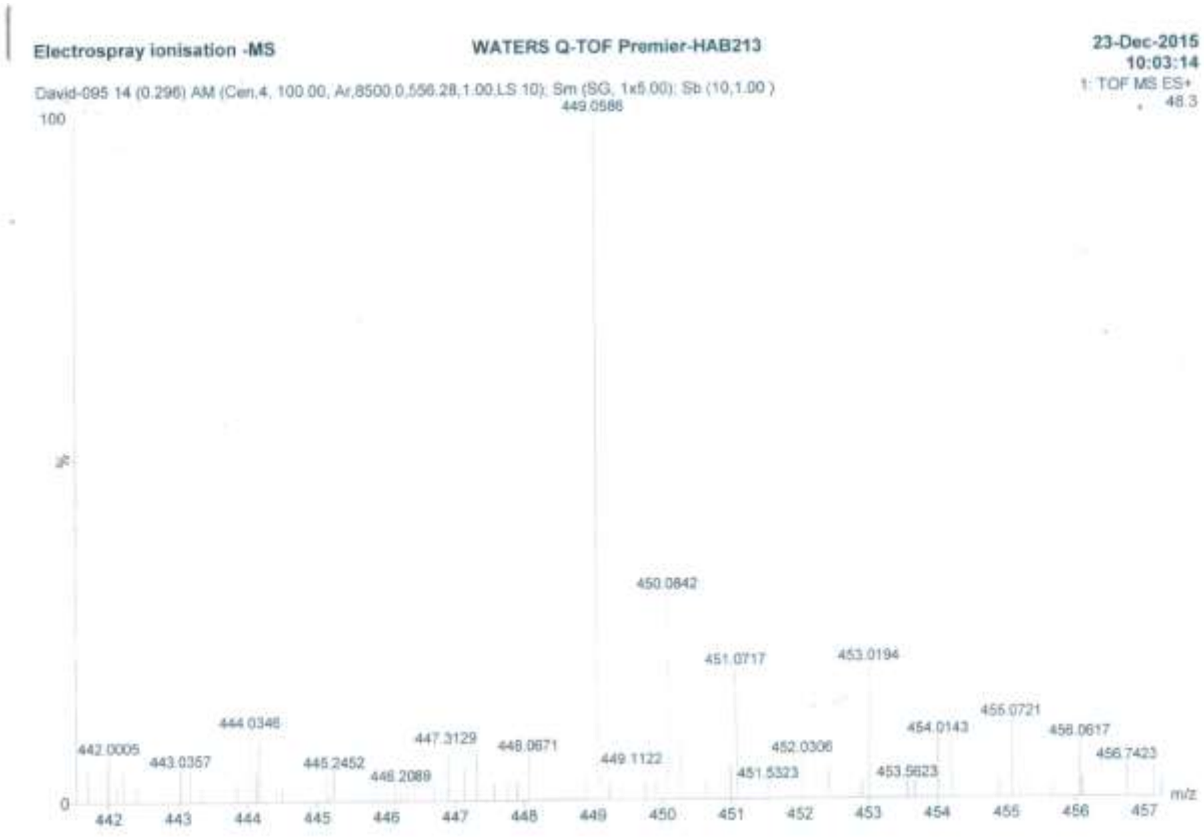

Electrospray ionisation -MS
WATERS Q-TOF Premier-HAB213
23-Dec-2015
10:03:14
David-095 14 (0.296) AM (Cen,4, 100.00, Ar,8500.0,556.28,1.00,LS 10); Sm (SG, 1x5.00); Sb (10,1.00)
1: TOF MS ES+
48.3
449.0586
450.0842
451.0717
453.0194
455.0721
454.0143
456.0617
456.7423
452.0306
451.5323
453.5623
449.1122
448.0671
447.3129
446.2089
445.2452
444.0346
443.0357
442.0005
m/z

17h

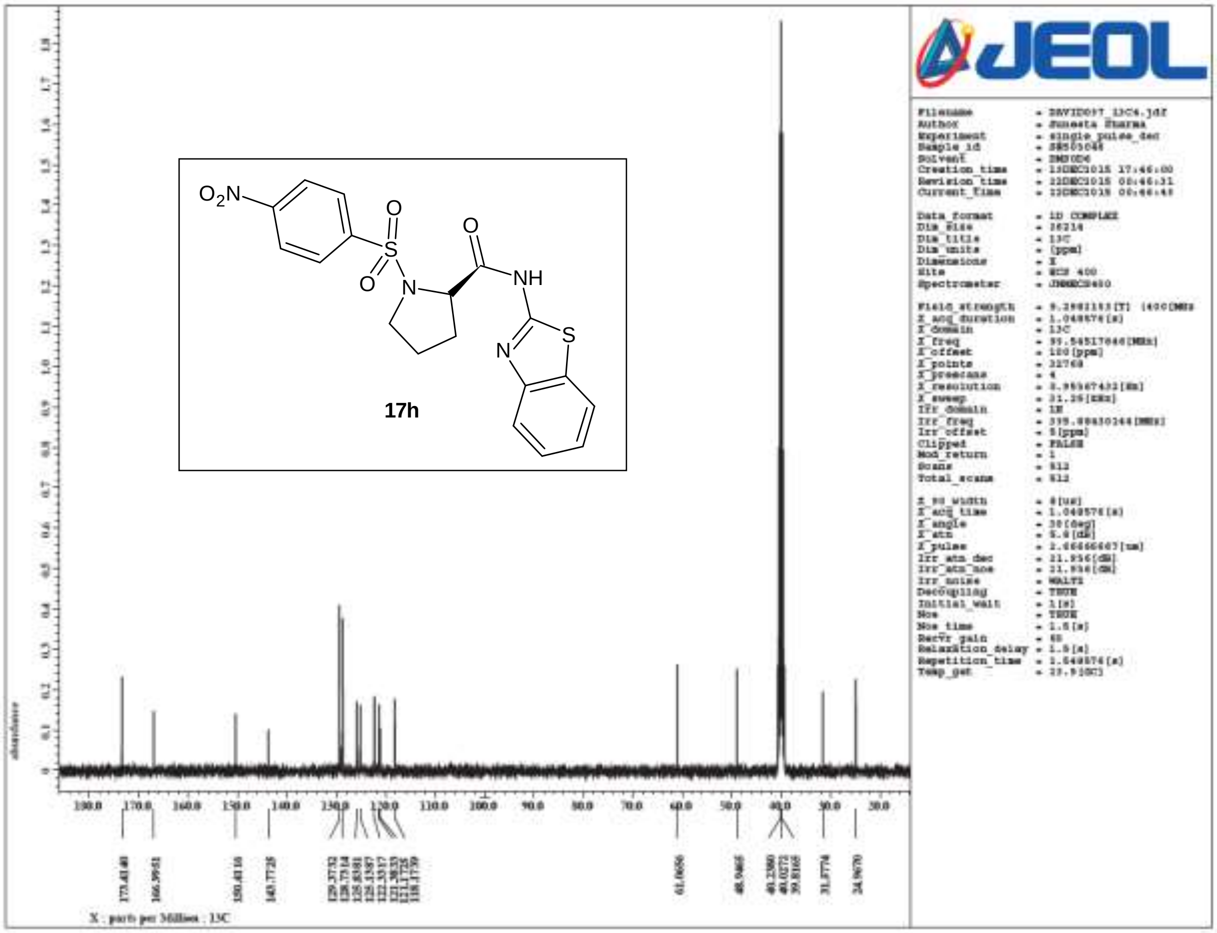

JEOL
O2N
N
O
S
NH
17h
abundance
173.4140
166.3951
150.4116
143.7728
61.0656
48.9465
40.2380
40.0272
39.8165
31.3774
24.9670
X : parts per Million : 13C

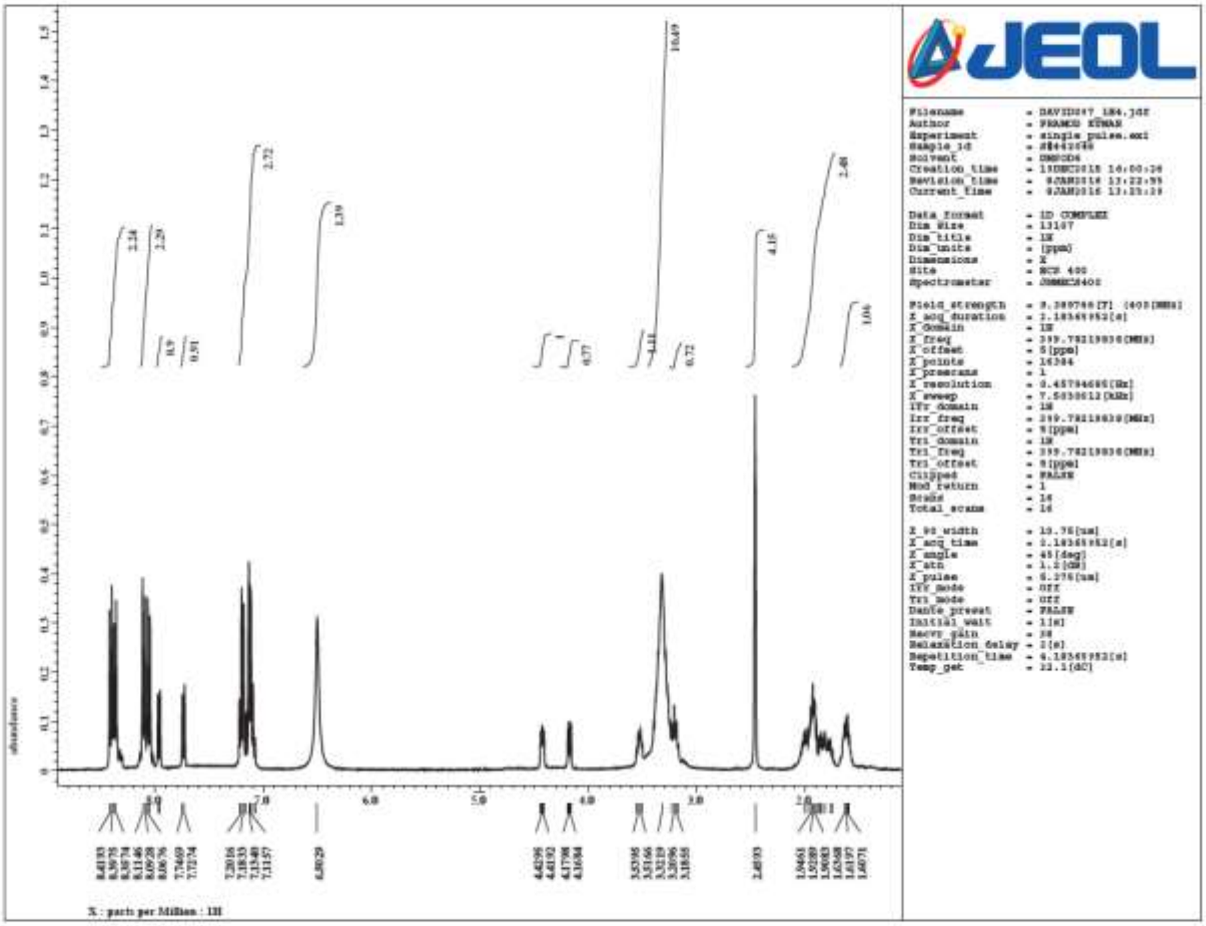
JEOL
Filename
Author
Experiment = single_pulse.exi
Sample_id
Solvent
Creation_time
Revision_time
Current_time
Data_format = 1D COMPLEX
Dim_size
Dim_title = 1H
Dim_units = [ppm]
Dimensions = X
Site
Spectrometer
Field_strength
X_acq_duration
X_domain = 1H
X_freq
X_offset = 5[ppm]
X_points = 16384
X_prescans = 1
X_resolution
X_sweep
Irr_domain = 1H
Irr_freq
Irr_offset
Tri_domain = 1H
Tri_freq
Tri_offset
Clipped = FALSE
Mod_return = 1
Scans = 16
Total_scans = 16
X_90_width
X_acq_time
X_angle = 45[deg]
X_atn
X_pulse
Irr_mode = Off
Tri_mode = Off
Dante_presat = FALSE
Initial_wait = 1[s]
Recvr_gain
Relaxation_delay = 2[s]
Repetition_time
Temp_get
abundance
X : parts per Million : 1H

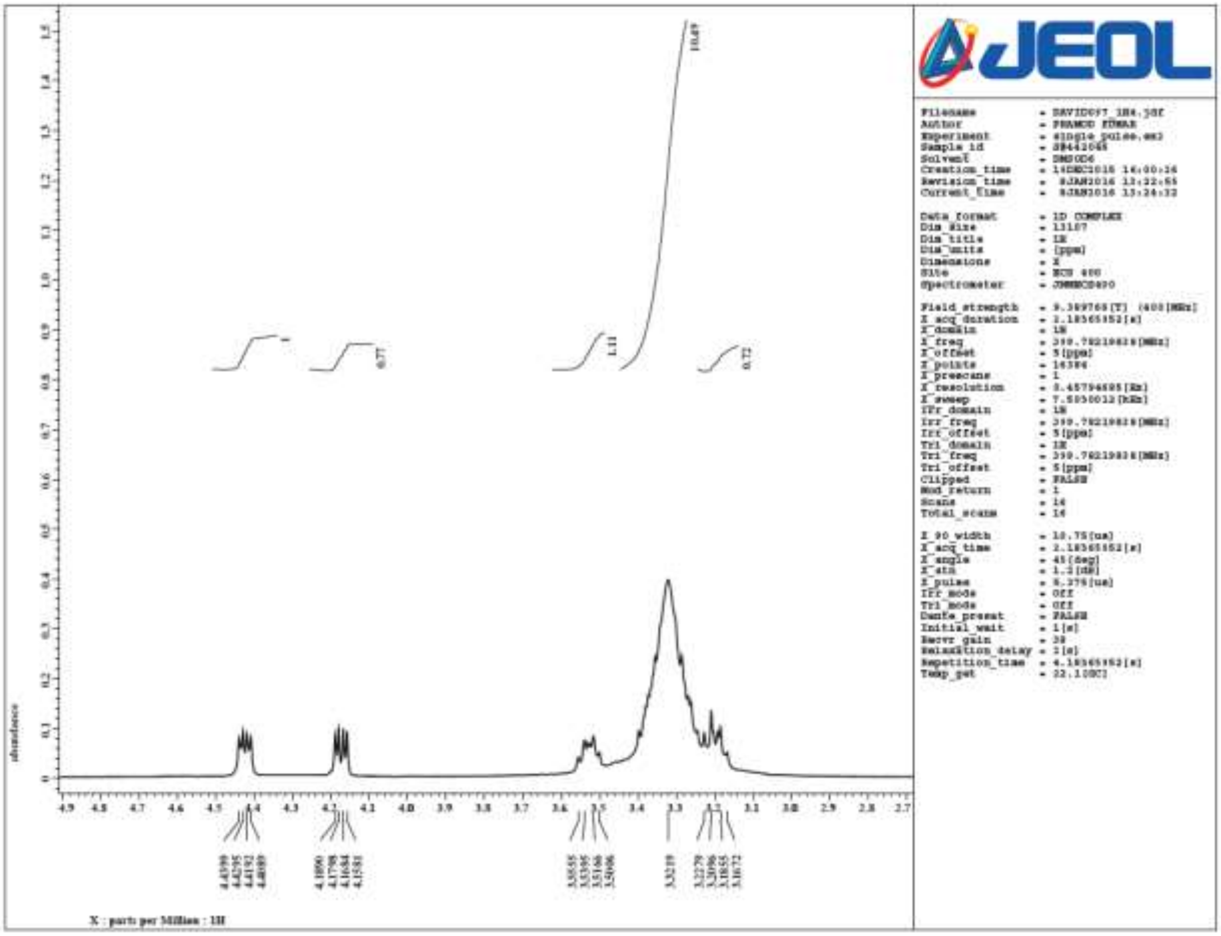

JEOL
Filename = DAVID097_1H4.jdf
Author = PRAMOD KUMAR
Experiment = single_pulse.ex2
Solvent = DMSO-D6
Creation_time = 14DEC2015 16:00:16
Revision_time = 8JAN2016 13:22:55
Current_time = 8JAN2016 13:24:32
Data_format = 1D COMPLEX
Dim_size = 13107
Dim_title = 1H
Dim_units = [ppm]
Dimensions = X
Site = ECS 400
Spectrometer = JNM-ECS400
Field_strength = 9.389766[T] (400[MHz])
X_acq_duration = 2.18365952[s]
X_domain = 1H
X_freq = 399.78219838[MHz]
X_offset = 5[ppm]
X_points = 16384
X_prescans = 1
X_resolution = 0.45794685[Hz]
X_sweep = 7.50300312[kHz]
Irr_domain = 1H
Irr_freq = 399.78219838[MHz]
Irr_offset = 5[ppm]
Tri_domain = 1H
Tri_freq = 399.78219838[MHz]
Tri_offset = 5[ppm]
Clipped = FALSE
Mod_return = 1
Scans = 16
Total_scans = 16
X_90_width = 10.75[us]
X_acq_time = 2.18365952[s]
X_angle = 45[deg]
X_atn = 1.3[dB]
X_pulse = 5.375[us]
Irr_mode = Off
Tri_mode = Off
Dante_presat = FALSE
Initial_wait = 1[s]
Recvr_gain = 38
Relaxation_delay = 2[s]
Repetition_time = 4.18365952[s]
Temp_get = 22.1[DC]
10.49
0.77
1.11
0.72
abundance
4.4399
4.4295
4.4192
4.4089
4.1890
4.1798
4.1684
4.1583
3.5555
3.5395
3.5166
3.5006
3.3219
3.2279
3.2096
3.1855
3.1672
X : parts per Million : 1H

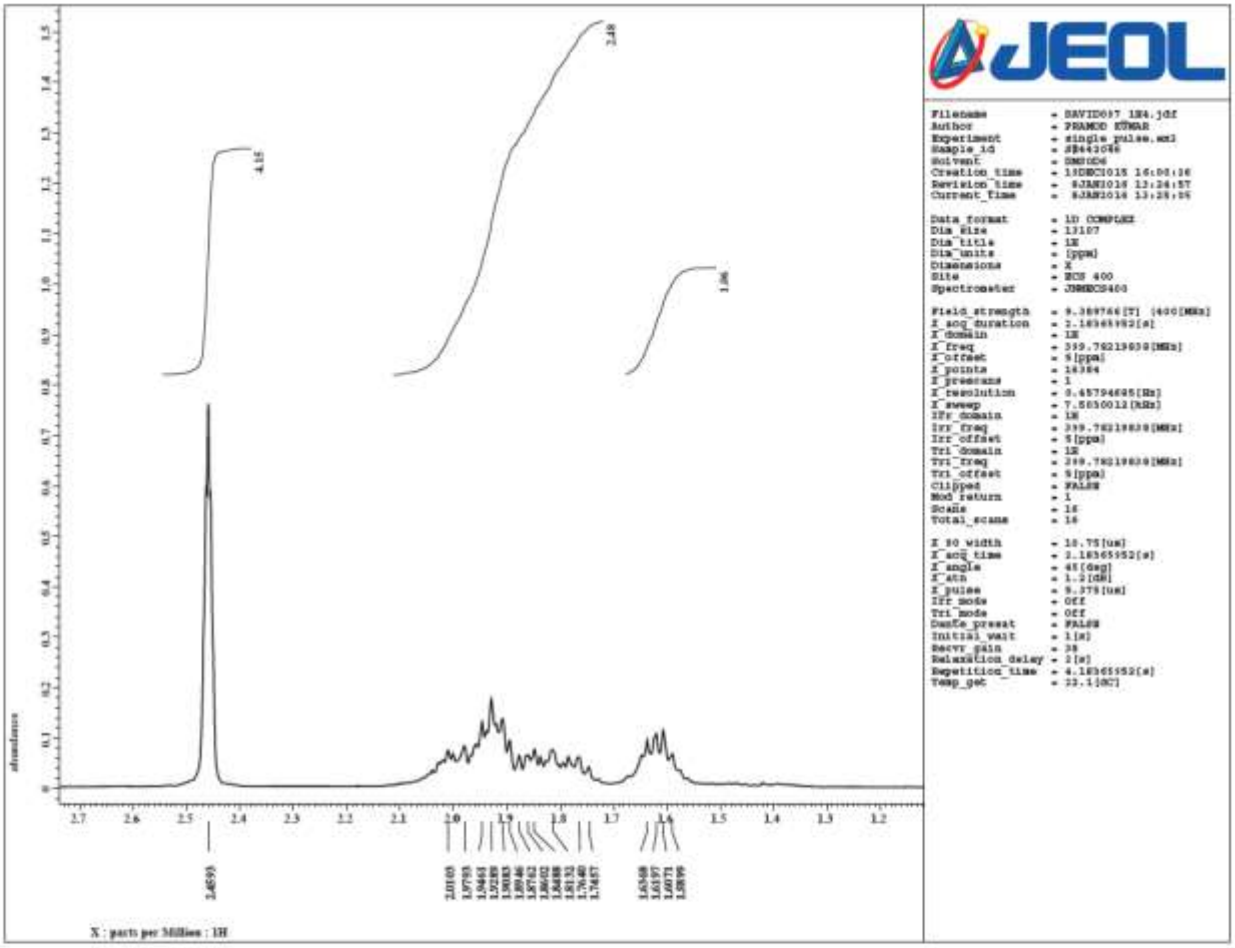
JEOL
Filename = DAVID037_1H4.jdf
Author = PRAMOD KUMAR
Experiment = single_pulse.ex2
Solvent = DMSOD6
Data_format = 1D COMPLEX
Dim_size = 13107
Dim_title = 1H
Dim_units = [ppm]
Dimensions = X
Site = ECS 400
Field_strength = 9.389766[T] (400[MHz])
X_domain = 1H
X_freq = 399.78219838[MHz]
X_offset = 5[ppm]
X_points = 16384
X_prescans = 1
X_resolution = 0.45794685[Hz]
X_sweep = 7.5030012[kHz]
Irr_domain = 1H
Irr_freq = 399.78219838[MHz]
Irr_offset = 5[ppm]
Tri_domain = 1H
Tri_freq = 399.78219838[MHz]
Tri_offset = 5[ppm]
Clipped = FALSE
Mod_return = 1
Scans = 16
Total_scans = 16
X_90_width = 10.75[us]
X_acq_time = 2.18365952[s]
X_angle = 45[deg]
X_atn = 1.2[dB]
X_pulse = 5.375[us]
Irr_mode = Off
Tri_mode = Off
Dante_presat = FALSE
Initial_wait = 1[s]
Recvr_gain = 38
Relaxation_delay = 2[s]
Repetition_time = 4.18365952[s]
Temp_get = 23.1[dC]
abundance
X : parts per Million : 1H

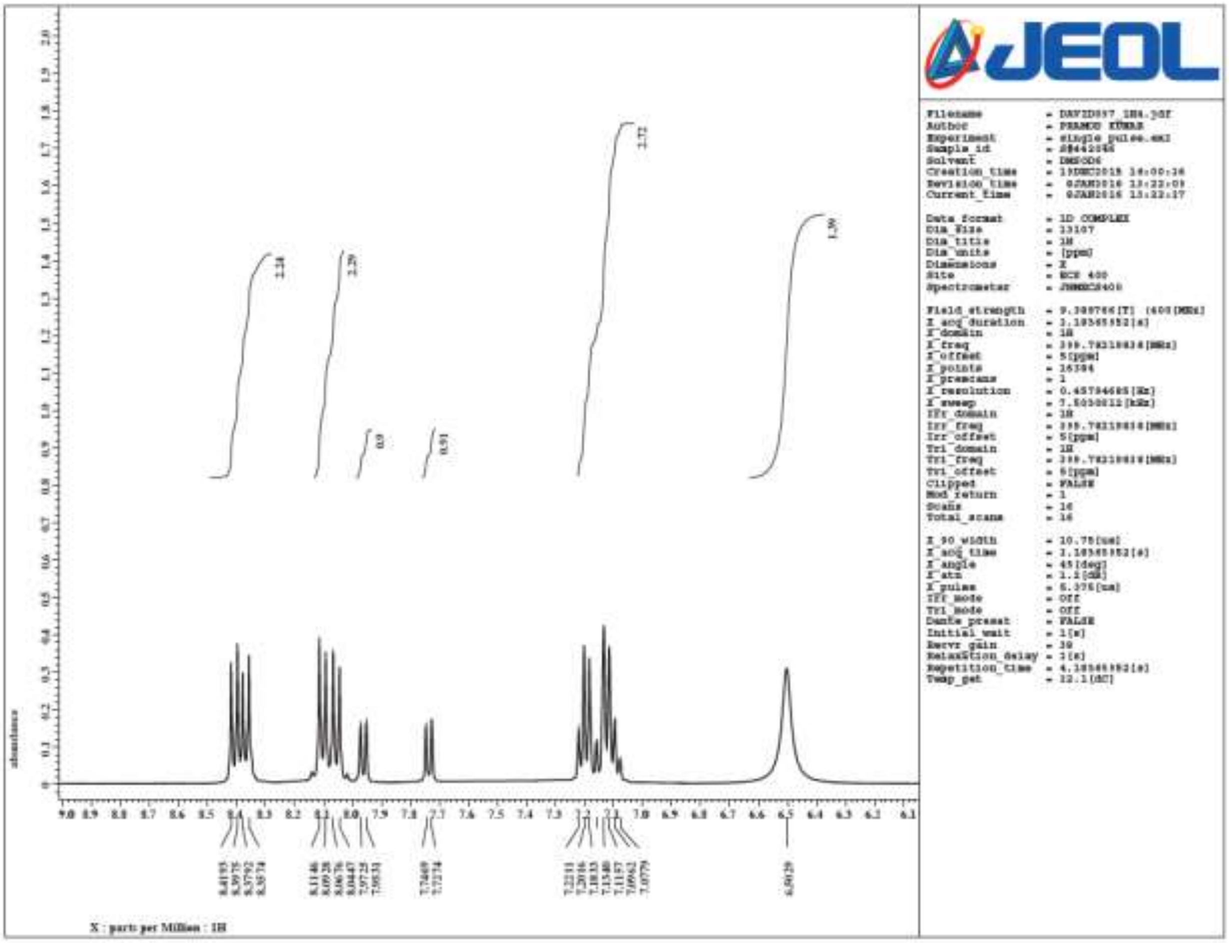

abundance
X : parts per Million : 1H
2.24
2.29
0.9
0.91
2.72
1.30
8.4193
8.3975
8.3792
8.3574
8.1146
8.0928
8.0676
8.0447
7.9725
7.9531
7.7469
7.7274
7.2211
7.2016
7.1833
7.1540
7.1157
7.0962
7.0779
6.4929
Filename = DAVID097_1H4.jdf
Author = PRAMOD KUMAR
Experiment = single_pulse.ex2
Solvent = DMSOD6
Data format = 1D COMPLEX
Dim_title = 1H
Dim_units = [ppm]
Dimensions = X
Site = ECS 400
Field_strength = 9.389766[T] (400[MHz])
X_domain = 1H
X_offset = 5[ppm]
X_points = 16384
X_prescans = 1
Irr_domain = 1H
Irr_offset = 5[ppm]
Tri_domain = 1H
Clipped = FALSE
Mod_return = 1
Scans = 16
Total_scans = 16
X_90_width = 10.75[us]
X_angle = 45[deg]
X_atn = 1.1[dB]
X_pulse = 5.375[us]
Irr_mode = Off
Tri_mode = Off
Dante_presat = FALSE
Initial_wait = 1[s]
Temp_get = 22.1[dC]

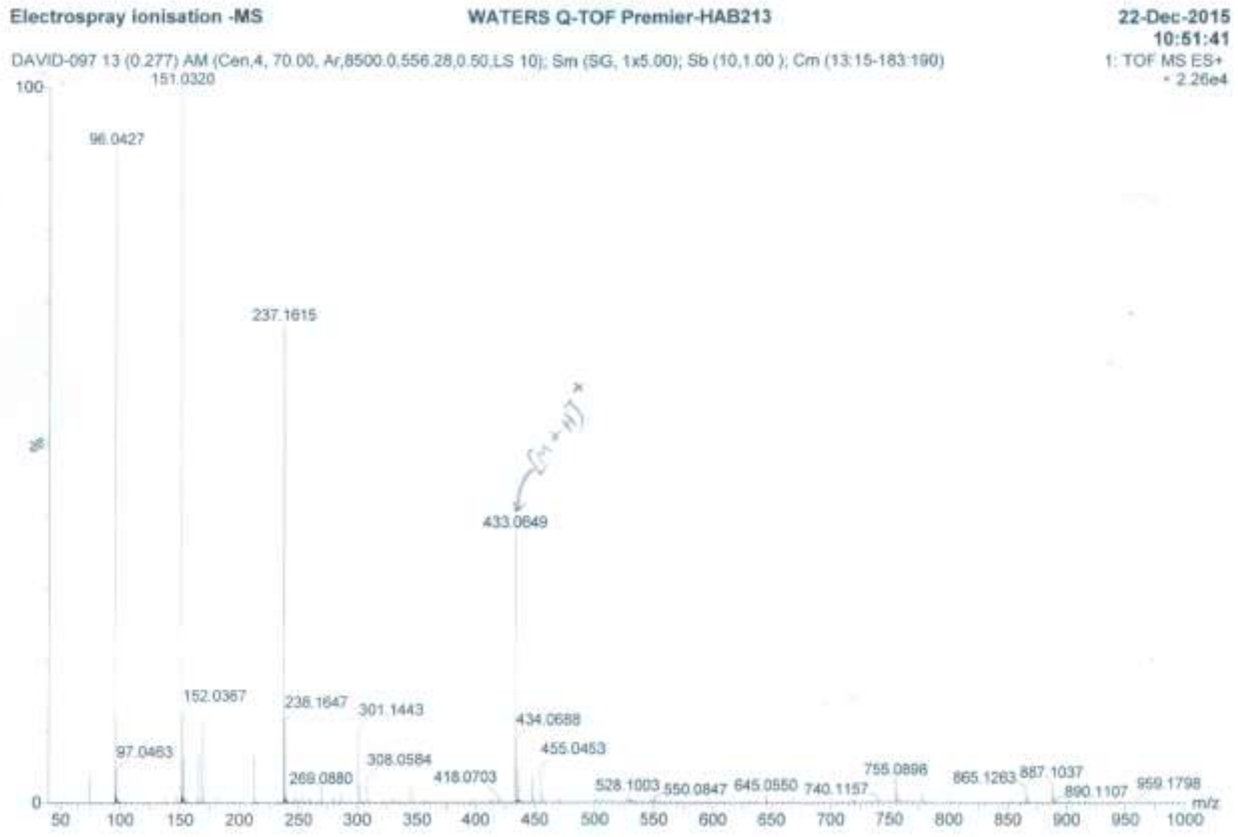

Electrospray ionisation -MS
WATERS Q-TOF Premier-HAB213
22-Dec-2015
10:51:41
DAVID-097 13 (0.277) AM (Cen,4, 70.00, Ar,8500.0,556.28,0.50,LS 10); Sm (SG, 1x5.00); Sb (10,1.00); Cm (13:15-183:190)
1: TOF MS ES+
2.26e4
151.0320
96.0427
237.1615
433.0649
152.0367
238.1647
301.1443
434.0688
97.0463
308.0584
455.0453
269.0880
418.0703
528.1003
550.0847
645.0550
740.1157
755.0896
865.1263
887.1037
890.1107
959.1798
m/z

17i

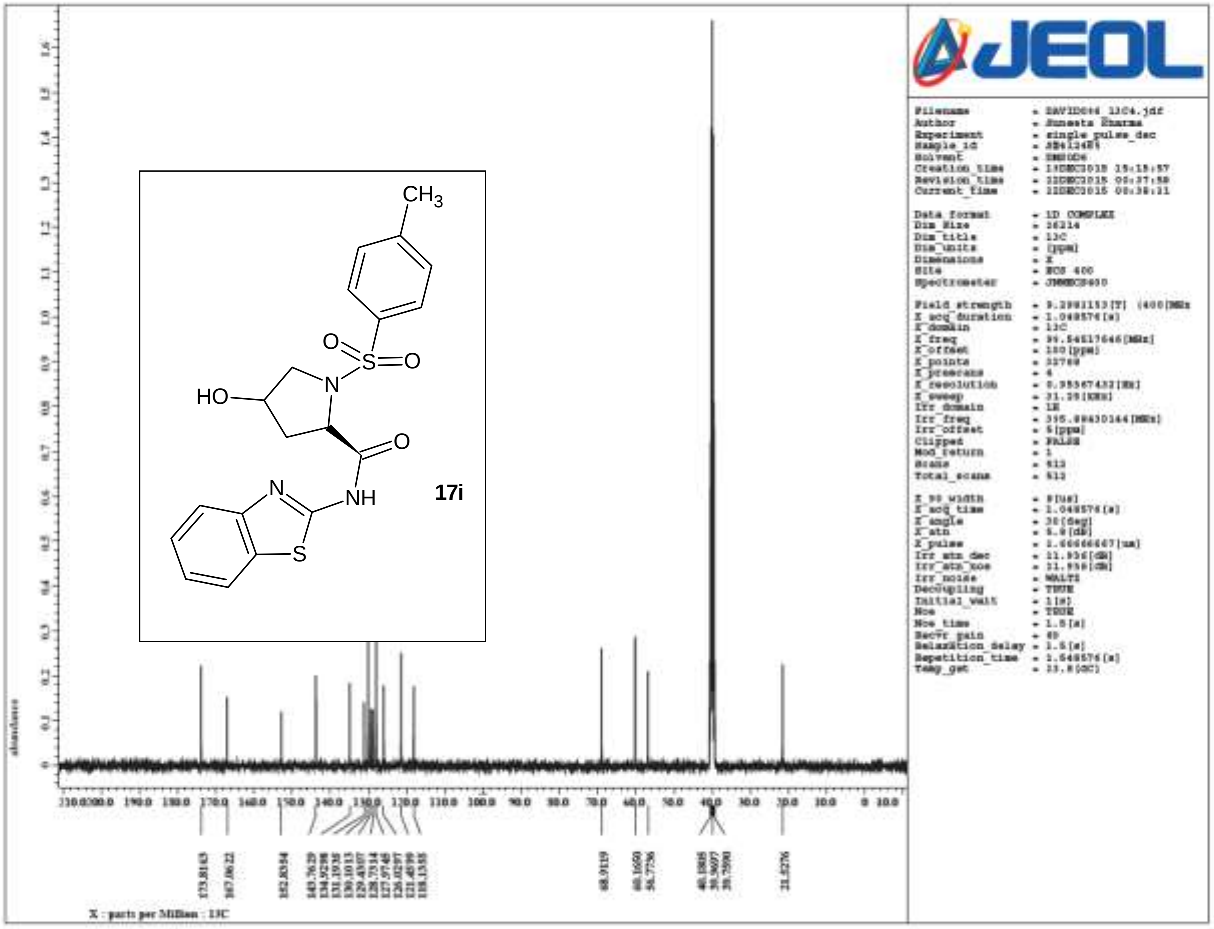

CH3
O
S
O
N
HO
O
NH
N
S
17i
JEOL
abundance
X : parts per Million : 13C
173.8143
167.0622
152.8354
143.7629
134.9298
131.1938
130.1013
129.4307
128.7314
127.9745
126.0297
121.4599
118.1353
68.9119
60.1650
56.7736
40.1805
39.9697
39.7590
21.8276

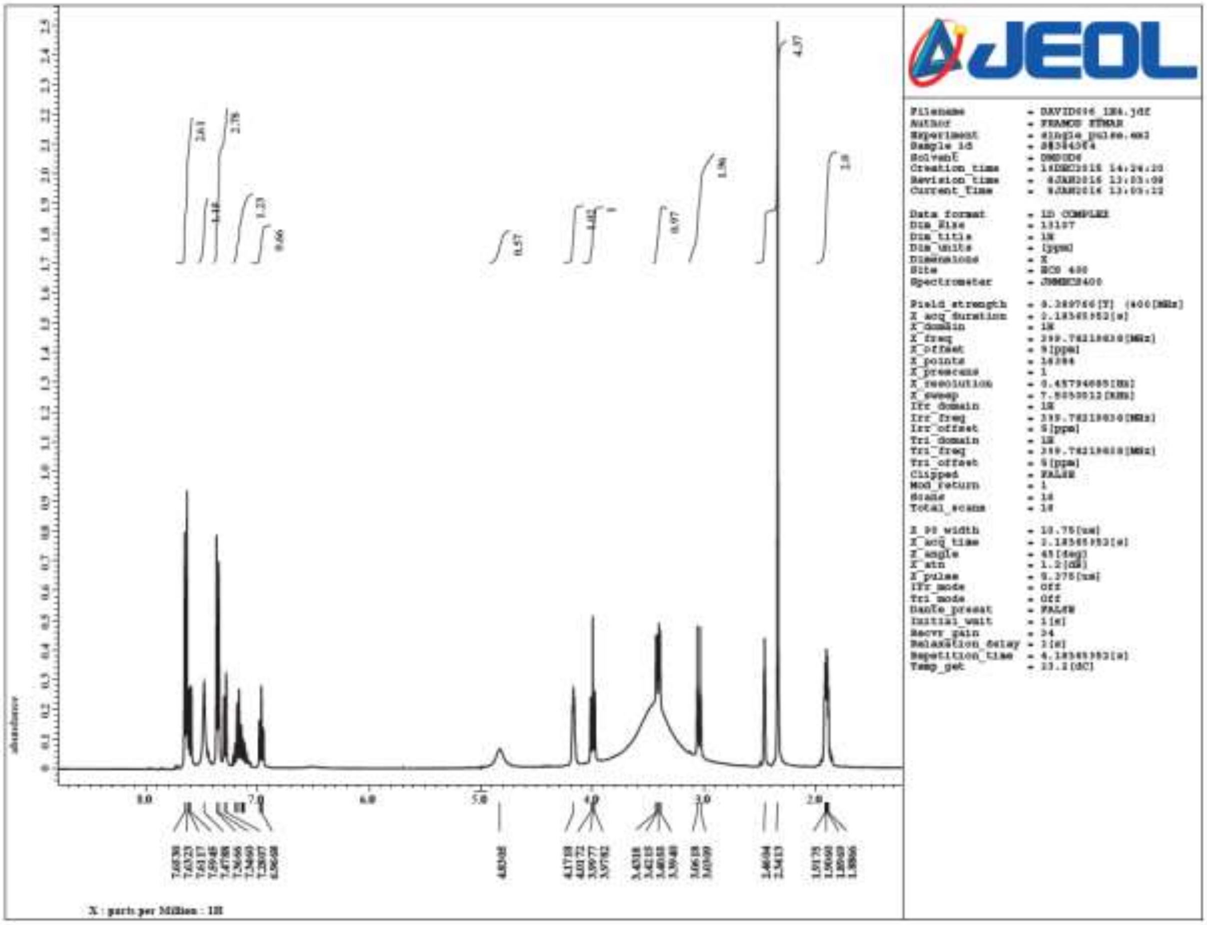

abundance
X : parts per Million : 1H
Filename = DAVID006_1H4.jdf
Experiment = single_pulse.ex2
Solvent = DMSO-D6
Data format = 1D COMPLEX
Dim_title = 1H
Dim_units = [ppm]
Spectrometer = JNMECS400
Field_strength = 9.389766[T] (400[MHz])
X_domain = 1H
X_freq = 399.78219838[MHz]
X_offset = 5[ppm]
X_points = 16384
X_prescans = 1
X_resolution = 0.45794685[Hz]
X_sweep = 7.5030012[kHz]
Irr_domain = 1H
Irr_offset = 5[ppm]
Tri_domain = 1H
Tri_offset = 5[ppm]
Clipped = FALSE
Mod_return = 1
Scans = 16
Total_scans = 16
X_90_width = 10.75[us]
X_angle = 45[deg]
X_atn = 1.2[dB]
X_pulse = 5.375[us]
Irr_mode = Off
Tri_mode = Off
Dante_presat = FALSE
Initial_wait = 1[s]
Recvr_gain = 34
Relaxation_delay = 2[s]
Temp_get = 23.2[dC]

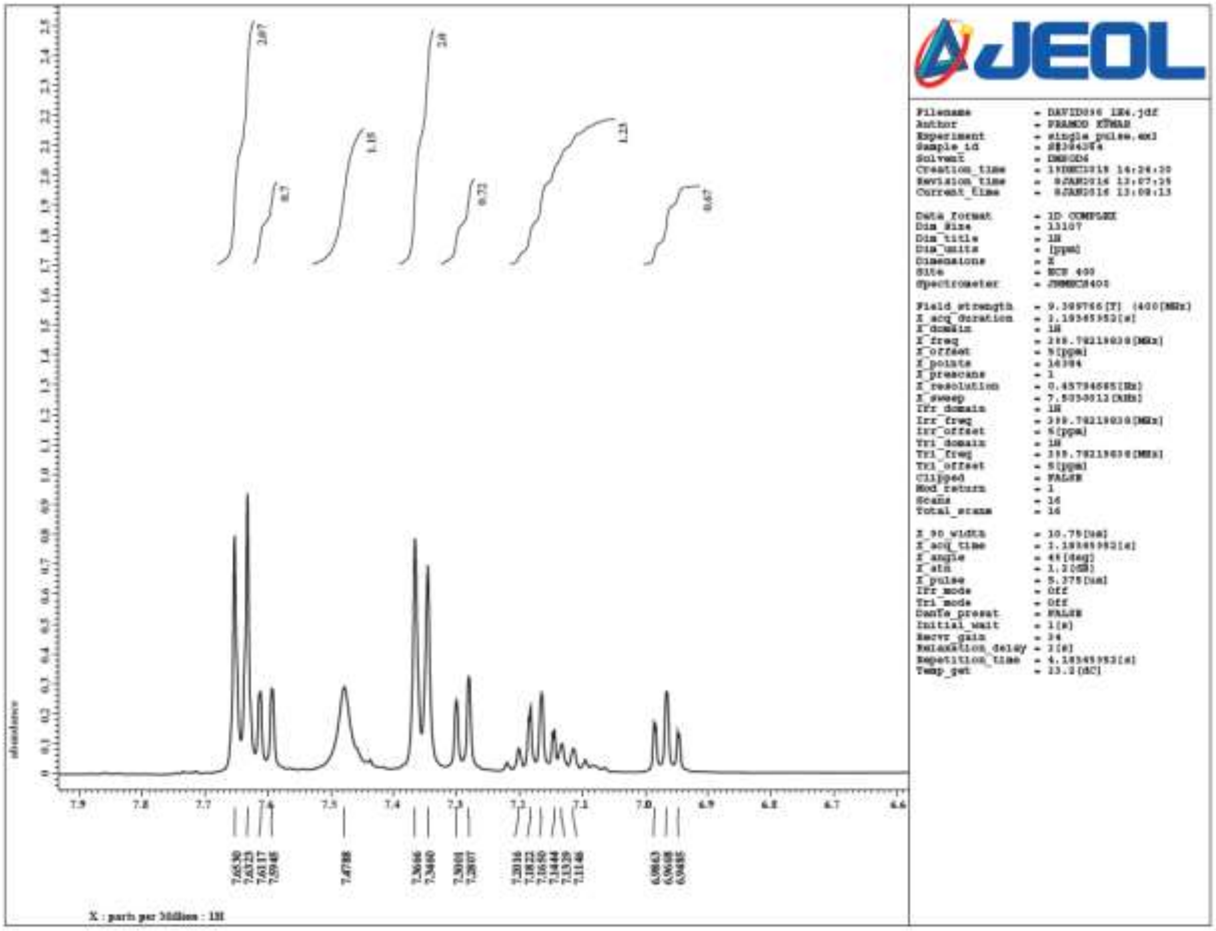

2.07
0.7
1.15
2.0
0.72
1.23
0.67
abundance
X : parts per Million : 1H
7.6530
7.6323
7.6117
7.5948
7.4788
7.3666
7.3480
7.3001
7.2807
7.2016
7.1822
7.1650
7.1444
7.1329
7.1146
6.9863
6.9668
6.9485
Filename = DAVID096 1H4.jdf
Experiment = single_pulse.ex2
Data format = 1D COMPLEX
Dim_size = 13107
Dim_title = 1H
Dim_units = [ppm]
Dimensions = X
Field_strength = 9.389766[T] (400[MHz])
X_domain = 1H
X_freq = 399.78219838[MHz]
X_offset = 5[ppm]
X_points = 16384
X_prescans = 1
X_resolution = 0.45794685[Hz]
X_sweep = 7.5030012[kHz]
Irr_domain = 1H
Irr_freq = 399.78219838[MHz]
Irr_offset = 5[ppm]
Tri_domain = 1H
Tri_freq = 399.78219838[MHz]
Tri_offset = 5[ppm]
Clipped = FALSE
Mod_return = 1
Scans = 16
Total_scans = 16
X_90_width = 10.75[us]
X_angle = 45[deg]
X_pulse = 5.375[us]
Irr_mode = Off
Tri_mode = Off
Dante_presat = FALSE
Initial_wait = 1[s]
Temp_get = 23.2[dC]

17j

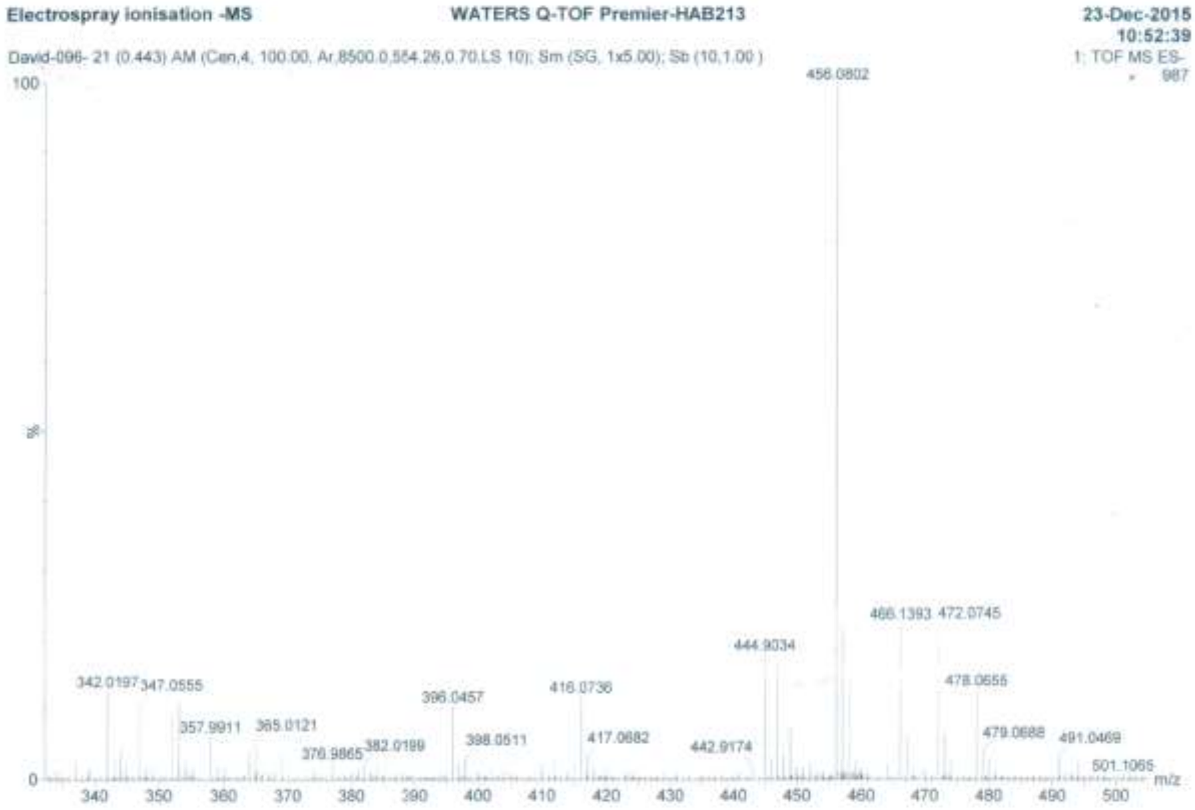

Electrospray ionisation -MS
WATERS Q-TOF Premier-HAB213
23-Dec-2015
10:52:39
David-096- 21 (0.443) AM (Cen,4, 100.00, Ar,8500.0,554.26,0.70,LS 10); Sm (SG, 1x5.00); Sb (10,1.00)
1: TOF MS ES-
987
456.0802
466.1393
472.0745
444.9034
478.0655
342.0197
347.0555
396.0457
416.0736
357.9911
365.0121
376.9865
382.0199
398.0511
417.0682
442.9174
479.0688
491.0469
501.1065
%
m/z

17k

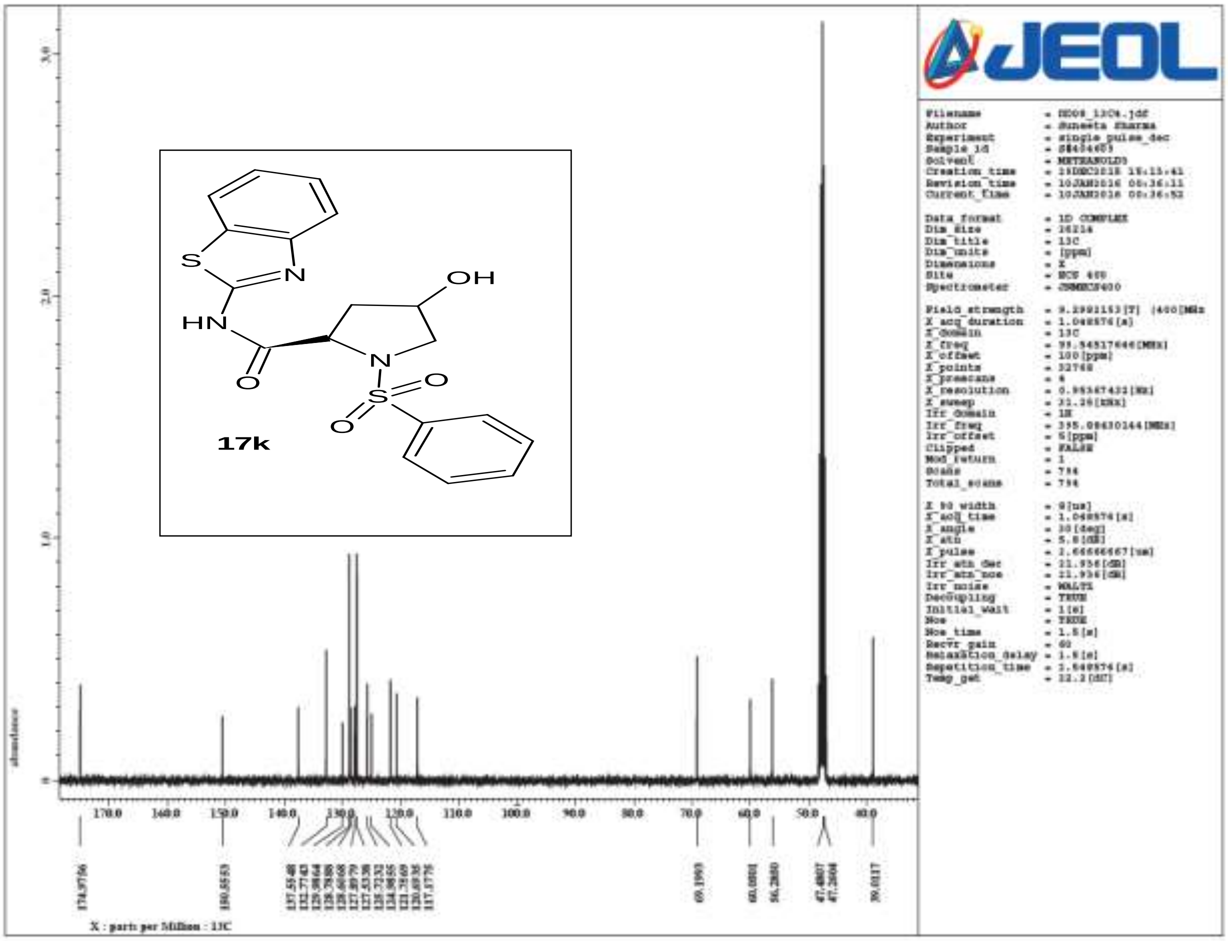

17k
X : parts per Million : 13C

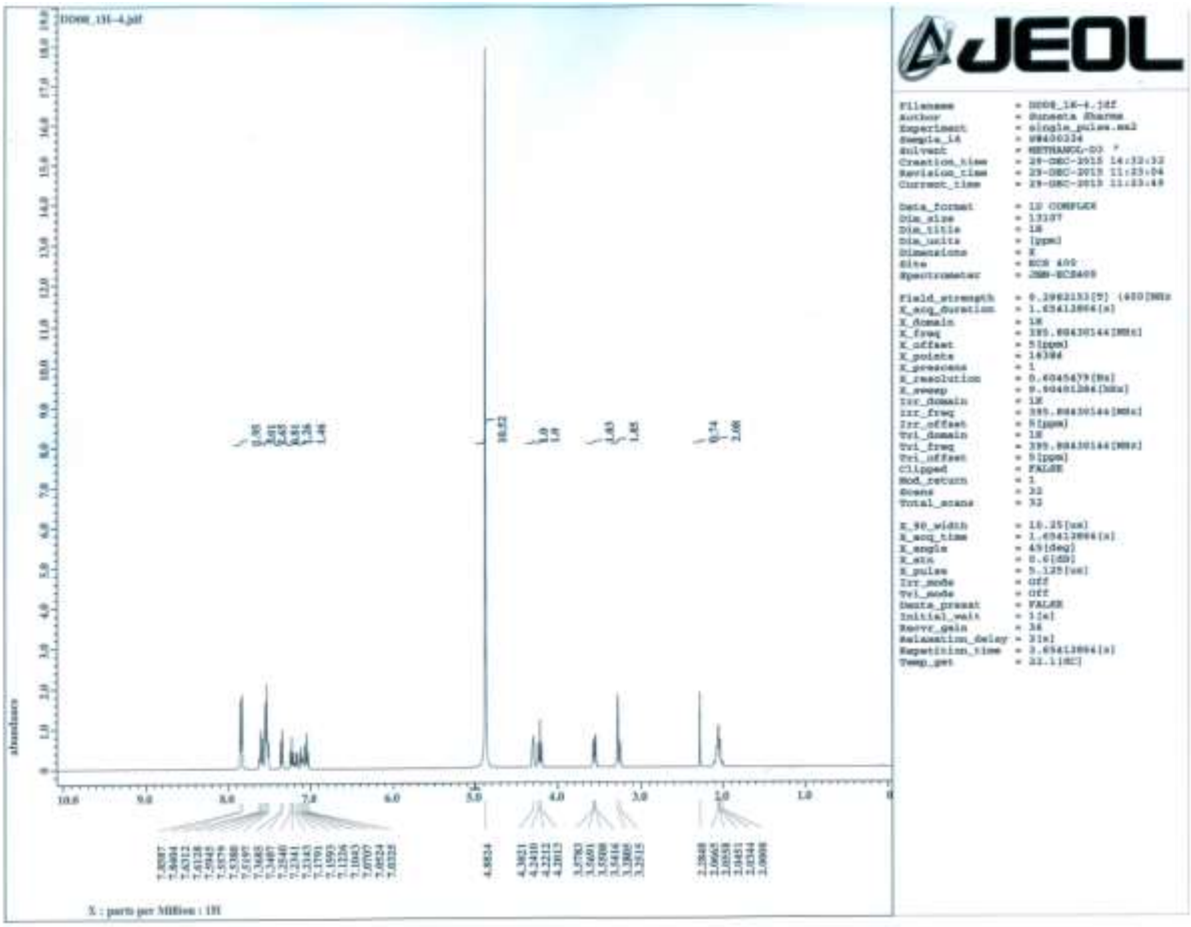
JEOL
abundance
X : parts per Million : 1H

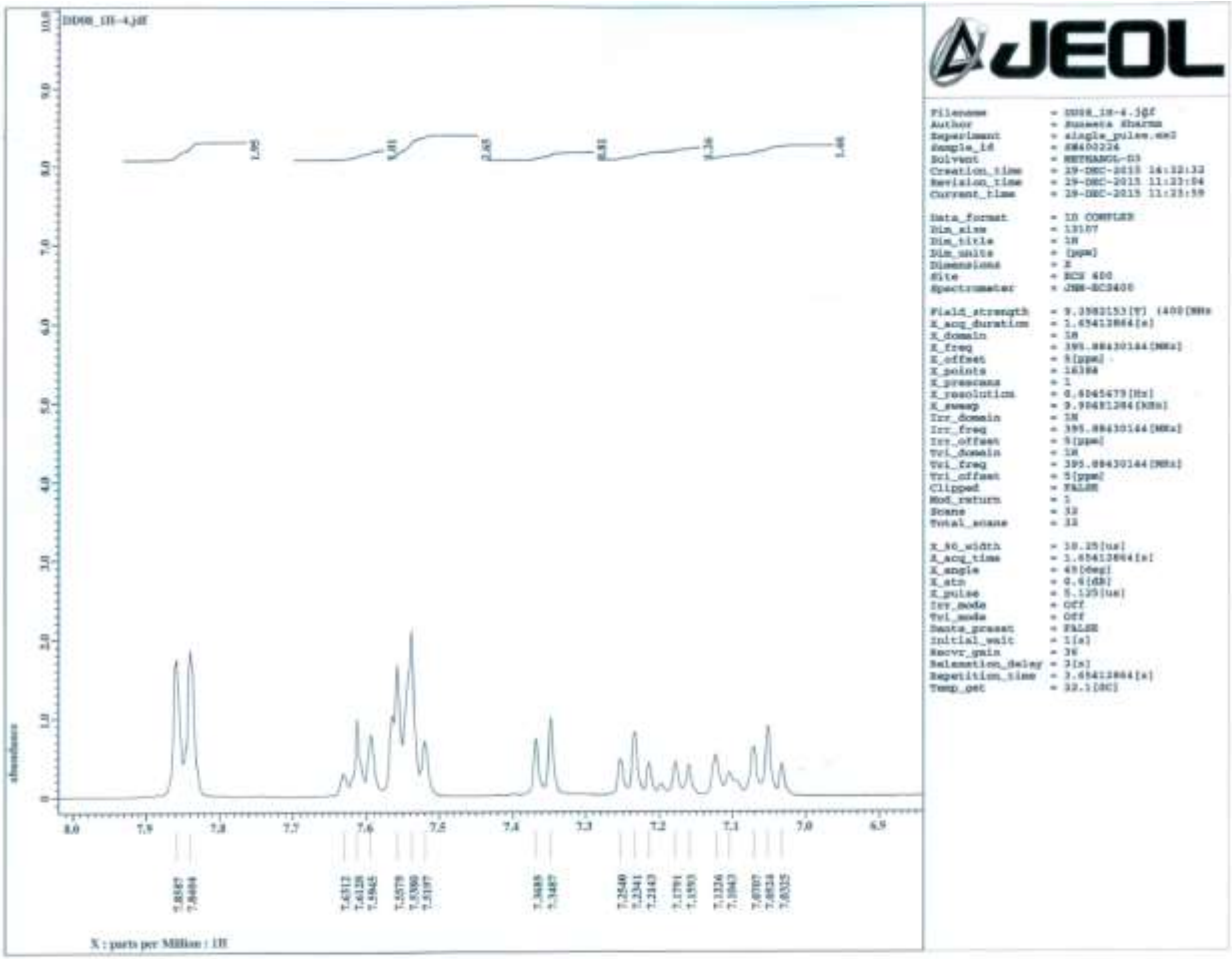
DD08_1H-4.jdf
X : parts per Million : 1H
7.8587
7.6312
7.6128
7.5945
7.5579
7.5380
7.5197
7.3688
7.3487
7.2540
7.2341
7.2143
7.1791
7.1593
7.1326
7.0707
7.0524
7.0325
Author = Suneeta Sharma
Experiment = single_pulse.ex2
Solvent = METHANOL-D3
Creation_time = 29-DEC-2015 14:32:32
Revision_time = 29-DEC-2015 11:23:04
Current_time = 29-DEC-2015 11:23:59
Data_format = 1D COMPLEX
Dim_size = 13107
Dim_title = 1H
Dim_units = [ppm]
Dimensions = X
Site = ECS 400
Spectrometer = JNM-ECS400
X_domain = 1H
X_freq = 395.88430144[MHz]
X_offset = 5[ppm]
X_points = 16384
X_prescans = 1
Irr_domain = 1H
Irr_freq = 395.88430144[MHz]
Irr_offset = 5[ppm]
Tri_domain = 1H
Tri_freq = 395.88430144[MHz]
Tri_offset = 5[ppm]
Clipped = FALSE
Mod_return = 1
Scans = 32
Total_scans = 32
X_angle = 45[deg]
X_pulse = 5.125[us]
Irr_mode = Off
Tri_mode = Off
Dante_presat = FALSE
Initial_wait = 1[s]
Recvr_gain = 36
Relaxation_delay = 2[s]

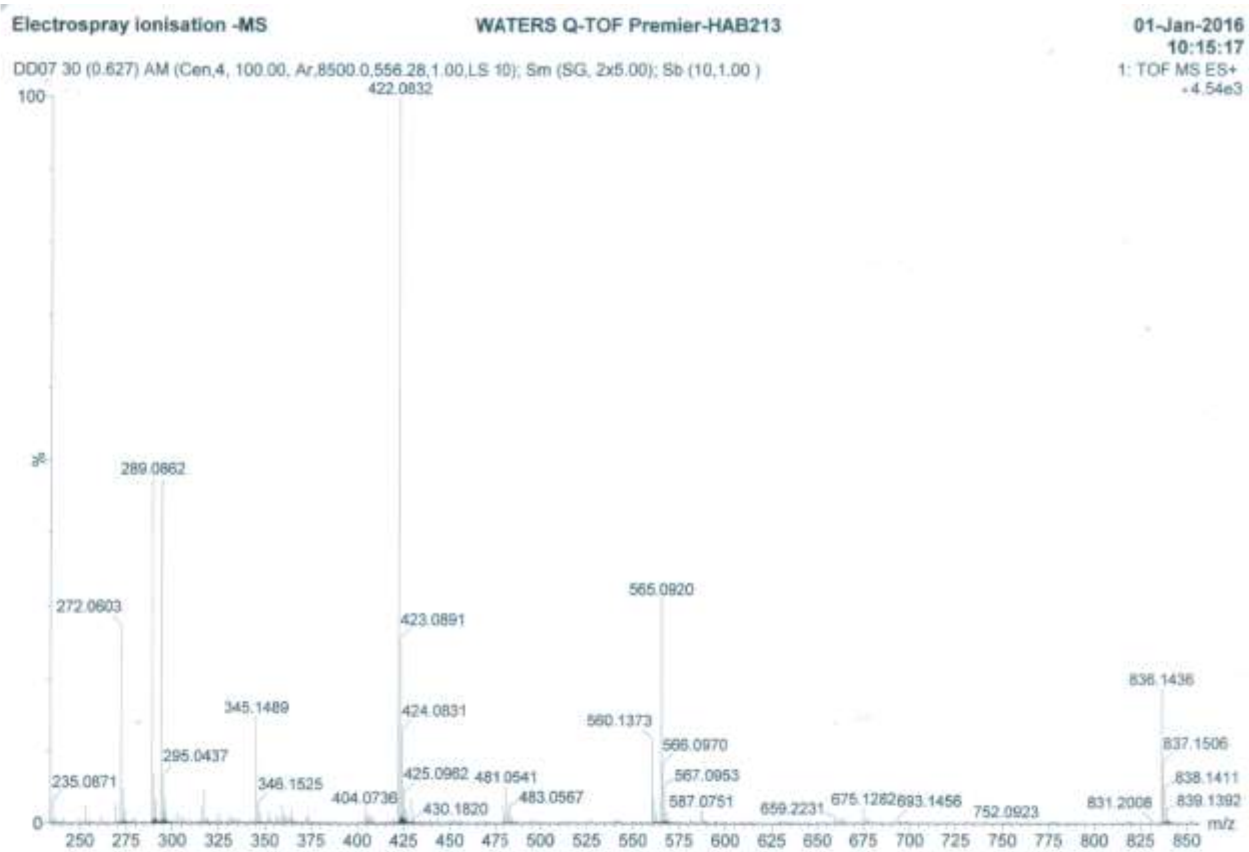

Electrospray ionisation -MS
WATERS Q-TOF Premier-HAB213
01-Jan-2016
10:15:17
DD07 30 (0.627) AM (Cen,4, 100.00, Ar,8500.0,556.28,1.00,LS 10); Sm (SG, 2x5.00); Sb (10,1.00)
1: TOF MS ES+
4.54e3
422.0832
289.0862
272.0603
565.0920
423.0891
836.1436
345.1489
424.0831
560.1373
566.0970
837.1506
295.0437
235.0871
346.1525
425.0962
481.0541
567.0953
838.1411
404.0736
483.0567
430.1820
587.0751
659.2231
675.1282
693.1456
752.0923
831.2008
839.1392
m/z

17l

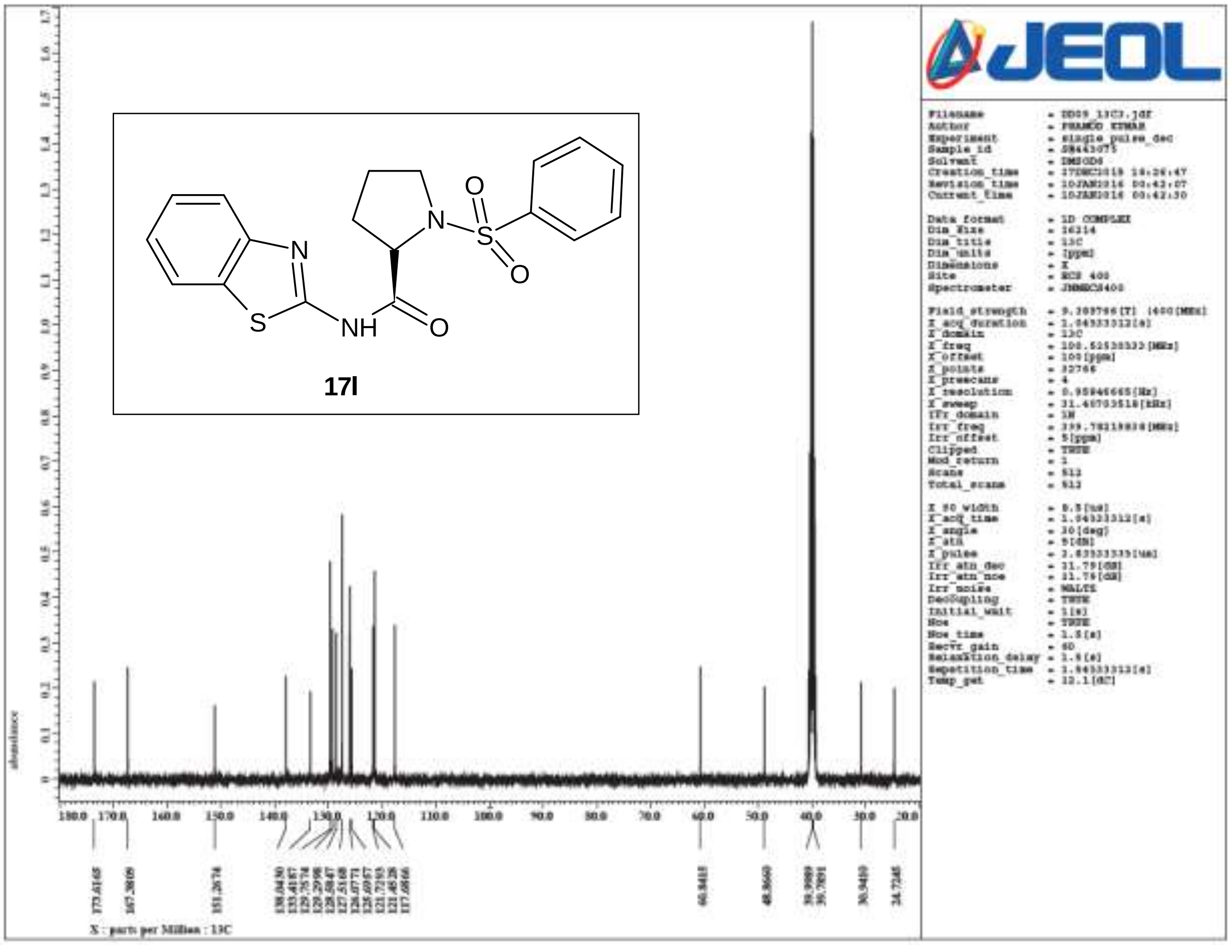
JEOL
17l
X : parts per Million : 13C

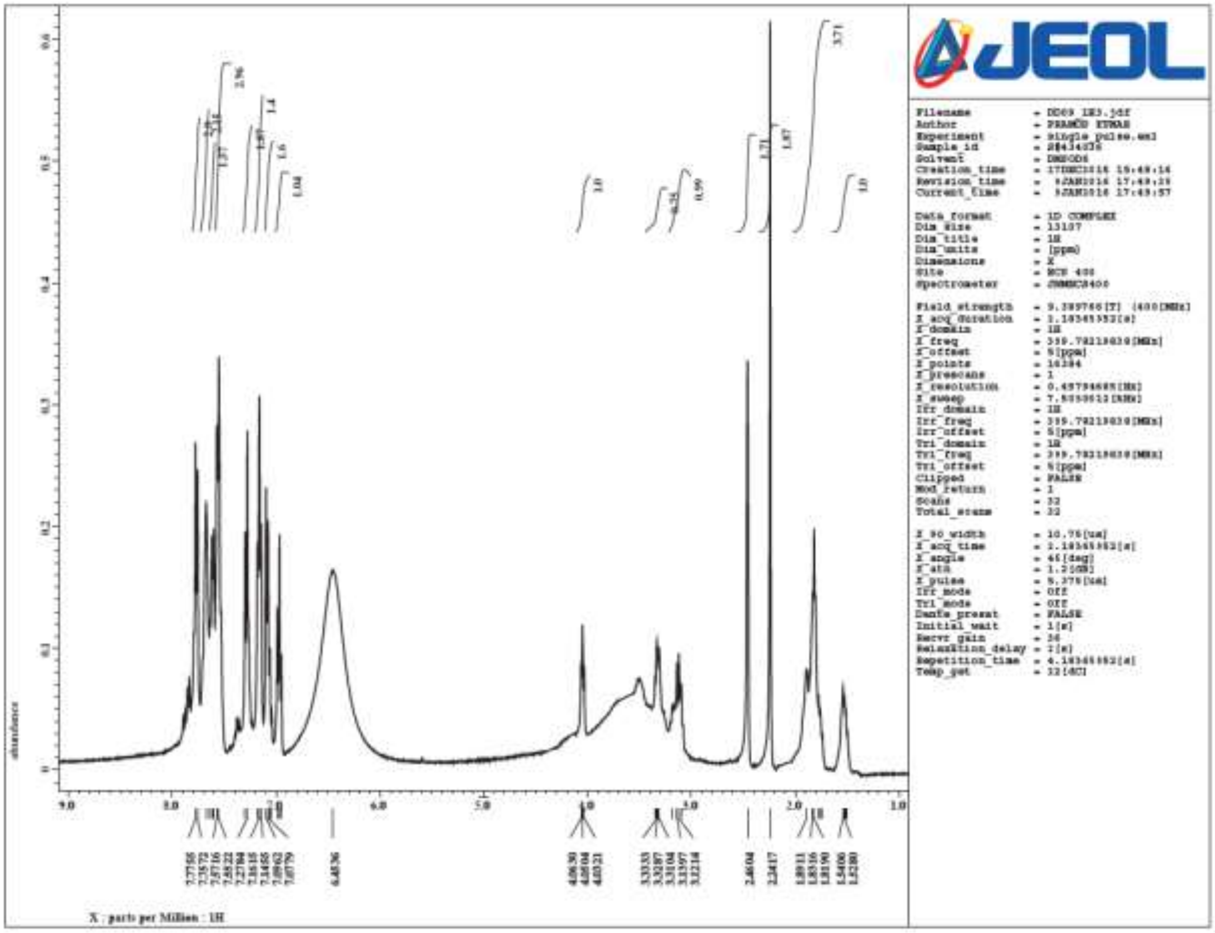

JEOL
Filename = DD09_1H3.jdf
Experiment = single_pulse.ex2
Solvent = DMSO-D6
Data_format = 1D COMPLEX
Dim_title = 1H
Dim_units = [ppm]
Dimensions = X
Site = ECS 400
Field_strength = 9.389766[T] (400[MHz])
X_domain = 1H
X_freq = 399.78219838[MHz]
X_offset = 5[ppm]
X_points = 16384
X_prescans = 1
Irr_domain = 1H
Irr_freq = 399.78219838[MHz]
Irr_offset = 5[ppm]
Tri_domain = 1H
Tri_freq = 399.78219838[MHz]
Tri_offset = 5[ppm]
Clipped = FALSE
Mod_return = 1
Scans = 32
Total_scans = 32
X_90_width = 10.75[us]
X_angle = 45[deg]
X_atn = 1.2[dB]
Irr_mode = Off
Tri_mode = Off
Dante_presat = FALSE
Initial_wait = 1[s]
Relaxation_delay = 2[s]
Temp_get = 22[dC]
7.7755
7.7572
7.6716
7.6522
7.2784
7.1615
7.1455
7.0962
7.0779
6.4836
4.0630
4.0504
4.0321
3.3333
3.3287
3.3104
3.1397
3.1214
2.4604
2.2417
1.8931
1.8516
1.8190
1.5406
1.5280
abundance
X : parts per Million : 1H

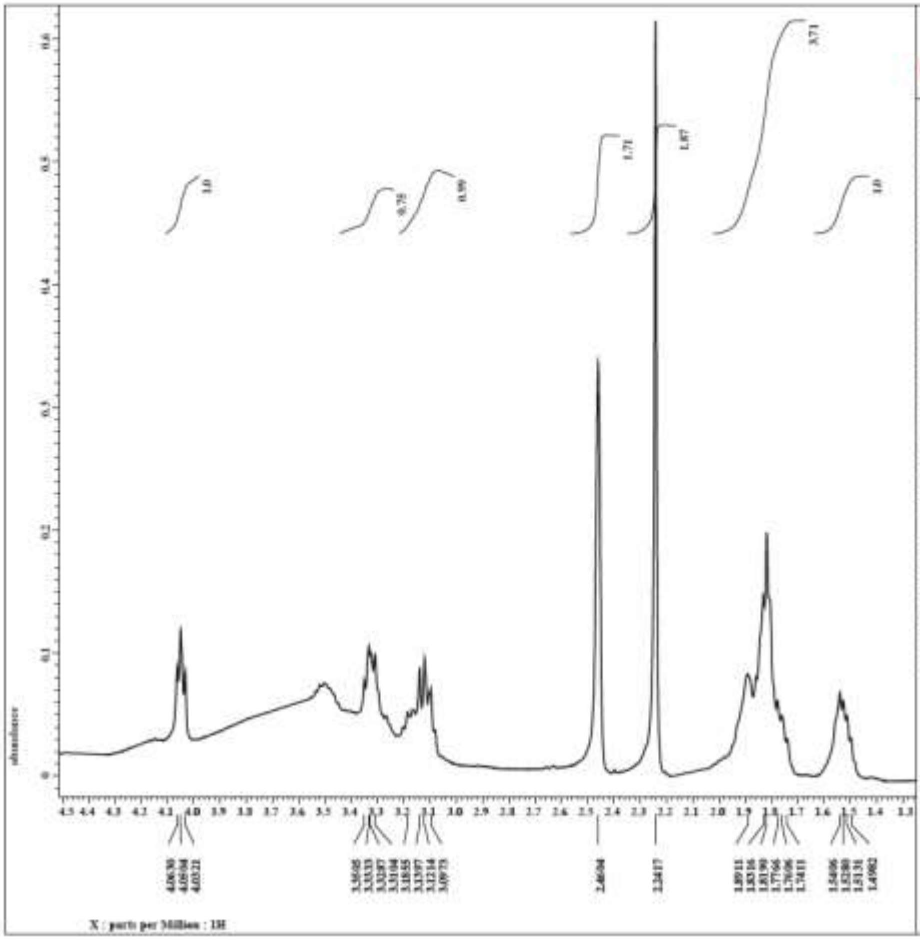


```
Filename          = DD09_1H3.jdf
Author            = FRANCO ETHAN
Experiment        = single_pulse.ex2
Sample_id         = PB434036
Solvent           = DMSOD6
Creation_time     = 17DEC2015 15:46:16
Revision_time     =  9JAN2016 17:46:08
Current_time      =  9JAN2016 17:46:17

Data_format       = 1D COMPLEX
Dim_size          = 13107
Dim_title         = 1H
Dim_units         = [ppm]
Dimensions        = X
Site              = ECS 400
Spectrometer      = JNMECS400

Field_strength    = 9.389766[T] (400[MHz])
X_acq_duration    = 2.18365952[s]
X_domain          = 1H
X_freq            = 399.78219838[MHz]
X_offset          = 5[ppm]
X_points          = 16384
X_prescans        = 1
X_resolution      = 0.45794685[Hz]
X_sweep           = 7.5030012[kHz]
Irr_domain        = 1H
Irr_freq          = 399.78219838[MHz]
Irr_offset        = 5[ppm]
Tri_domain        = 1H
Tri_freq          = 399.78219838[MHz]
Tri_offset        = 5[ppm]
Clipped           = FALSE
Mod_return        = 1
Scans             = 32
Total_scans       = 32

X_90_width        = 10.75[us]
X_acq_time        = 2.18365952[s]
X_angle           = 45[deg]
X_atn             = 1.2[dB]
X_pulse           = 5.375[us]
Irr_mode          = Off
Tri_mode          = Off
Dante_presat      = FALSE
Initial_wait      = 1[s]
Recvr_gain        = 36
Relaxation_delay  = 2[s]
Repetition_time   = 4.18365952[s]
Temp_get          = 22[dC]
```

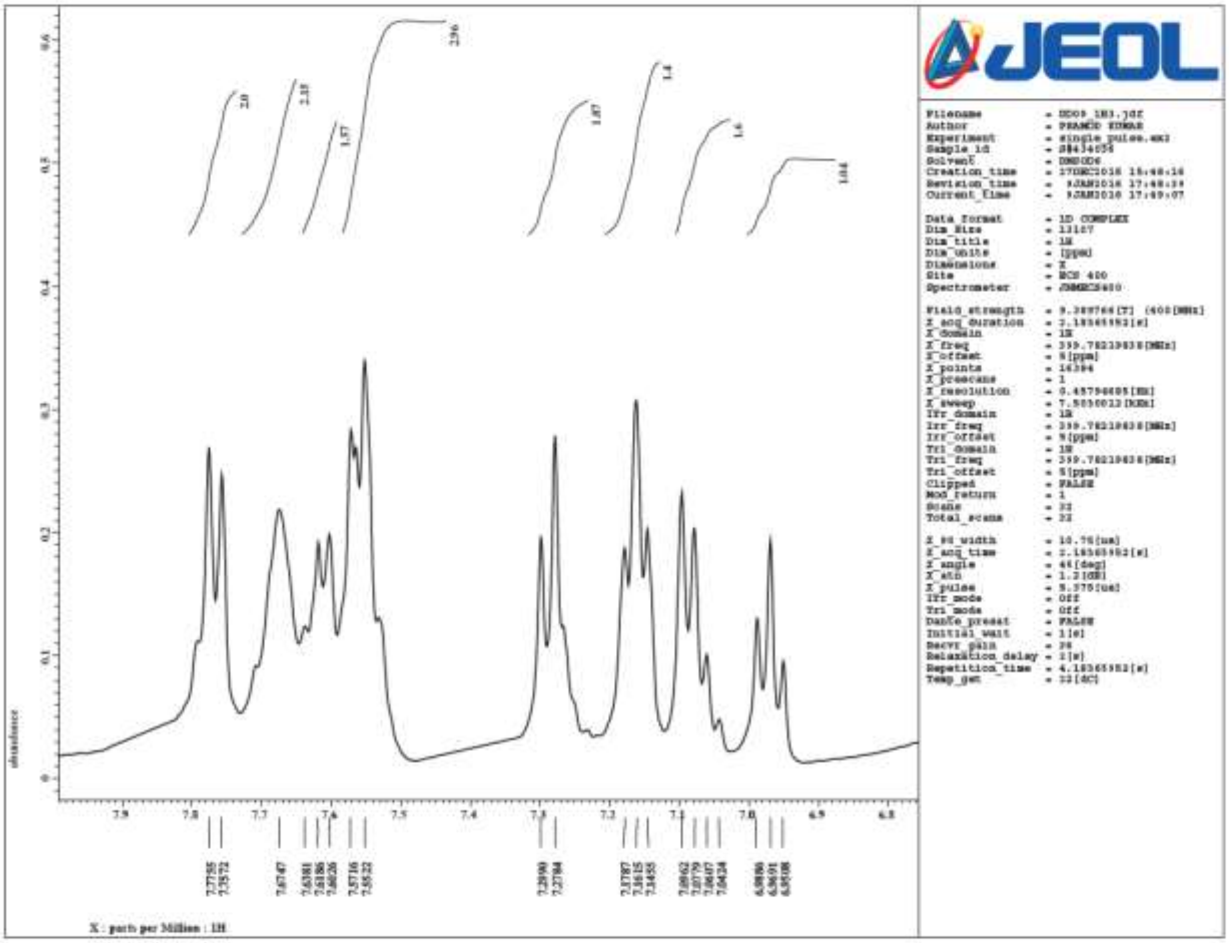

JEOL
X : parts per Million : 1H
abundance
Filename = DD09_1H3.jdf
Author = PRAMOD KUMAR
Experiment = single_pulse.ex2
Solvent = DMSO-D6
Data_format = 1D COMPLEX
Dim_title = 1H
Dim_units = [ppm]
Dimensions = X
Site = ECS 400
Field_strength = 9.389766[T] (400[MHz])
X_domain = 1H
X_offset = 5[ppm]
X_points = 16384
X_prescans = 1
Irr_domain = 1H
Irr_offset = 5[ppm]
Tri_domain = 1H
Tri_offset = 5[ppm]
Clipped = FALSE
Mod_return = 1
Scans = 32
Total_scans = 32
X_angle = 45[deg]
X_atn = 1.2[dB]
Irr_mode = Off
Tri_mode = Off
Dante_presat = FALSE
Initial_wait = 1[s]
Relaxation_delay = 2[s]
Temp_get = 22[dC]
7.7755
7.7572
7.6747
7.6381
7.6186
7.6026
7.5716
7.5532
7.2784
7.1787
7.1615
7.0962
7.0779
7.0607
7.0424
6.9886
6.9691
6.9508
2.0
2.15
1.57
2.96
1.87
1.4
1.6
1.04

Electrospray ionisation -MS | WATERS Q-TOF Premier-HAB213 | 29-Dec-2015 12:49:56

David_09+ 20 (0.424) AM (Cen,4, 100.00, Ar,8500.0,556.28,0.85,LS 10); Sm (SG, 2x5.00); Sb (10,1.00); Cm (20:24-155:163)

1: TOF MS ES+
4.21e3

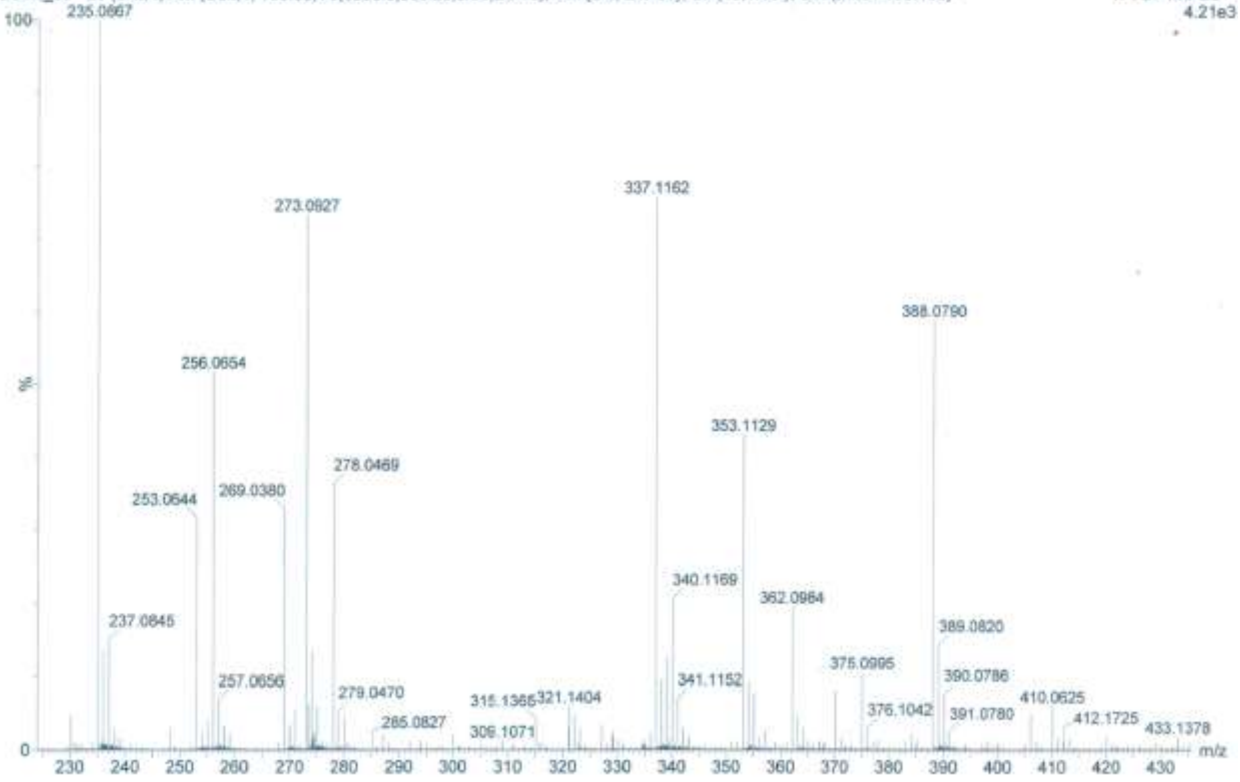

Supplement: IENZ_1426573_Supplementary_Material.pdf [file IENZ_A_1426573_SM8747.pdf]
